# Signpost Testing to Navigate the Parameter Space of the Gaussian Graphical Model With High‐Dimensional Data

**DOI:** 10.1002/bimj.70115

**Published:** 2026-02-12

**Authors:** Kai Ruan, Mark A. van de Wiel, Wessel N. van Wieringen

**Affiliations:** ^1^ Amsterdam UMC, location Vrije Universiteit Amsterdam Epidemiology and Data Science Amsterdam The Netherlands; ^2^ Amsterdam Public Health, Methodology Amsterdam The Netherlands; ^3^ Department of Mathematics Vrije Universiteit Amsterdam Amsterdam The Netherlands

**Keywords:** asymptotic distribution, bootstrap, directional hypothesis testing, *p*‐value, unbiasedness

## Abstract

We evaluate the relevance of external quantitative information on the parameter of a Gaussian graphical model from high‐dimensional data. This information comes in the form of a parameter value available from a related knowledge domain or population. We contrast the external information to ‘null’ information, i.e., an internally accepted parameter value. The direction from a null to this externally provided parameter value is dubbed the *signpost*. The signpost test evaluates whether to follow the signpost in the search of the true parameter value. We present various test statistics to measure the informativeness of the signpost and ways to obtain their distribution under the null hypothesis of non‐informativeness. By simulation, we investigate the power and other properties of the various signpost tests, and compare them to the likelihood ratio test. Finally, we employ the signpost test to illustrate how the learning of the Gaussian graphical model of a low‐prevalence breast cancer subtype benefits from external knowledge obtained from data of a more prevalent and related but fundamentally different subtype.

## 1 Introduction

Cellular regulatory pathways are often described stochastically by graphical models (Dobra et al. 2004). These models portray the pathway as a network. In this network, the nodes represent the entities comprising the pathway, and the edges connecting the nodes correspond to the molecular interactions among the nodes. The network topology is encoded in the graphical model's parameters. The model parameters and, subsequently, the network's topology are typically learned from scratch, often from high‐dimensional data. Apart from the challenges that this high‐dimensionality brings about, we need not necessarily start from scratch. Knowledge of the pathway's workings is often present from a related disease, context, or model organism. In this work, we present a test to assess whether that knowledge is relevant to the context at hand.

We consider data Y from the Gaussian graphical model, i.e., $Y∼\;N(0_{p},\Omega^{-1})$. The model parameter Ω is the precision matrix from the space of p×p‐dimensional, symmetric positive‐definite matrices $S_{p}^{++}$. The support of the precision matrix encodes the conditional (in)dependencies among the variates of this model. If ${\left(\Omega \right)}_{j,j^{\prime }}=0$ with $j,j^{\prime }\in \left\{1,\text{…},p\right\}$ and $j\neq j^{\prime }$, the variate pair $Y_{j}$ and $Y_{j^{\prime }}$ is independent, conditional on all other variates. Simultaneously, a nonzero off‐diagonal element of Ω indicates a conditional dependency between the corresponding variate pair. For more on graphical models, see Whittaker (1990) and Lauritzen (1996).

The learning of a Gaussian graphical model from high‐dimensional data is geared towards the reconstruction of the conditional (in)dependencies. This poses a challenging estimation and multiple testing problem. These may be overcome by regularization (e.g., Friedman et al. 2008) and multiple testing procedures (e.g., Benjamini and Hochberg 1995). The resulting inferred network topologies of these methods are unstable (Husmeier 2003; van Wieringen and Chen 2021). This is countered by procedures that assess the inferred solution's stability (Bodinier et al. 2021; Liu et al. 2010; Meinshausen and Bühlmann 2010; Müller et al. 2016).

Notwithstanding the sophistication of the aforementioned methodology to learn a Gaussian graphical model, it may be too much to ask to infer such details like a particular conditional independency from high‐dimensional data. High‐dimensional data are generated by high‐throughput techniques designed for automated screening purposes. Those techniques comprise many parallel gauges, usually of a lesser quality than the standard measurement, e.g., microarrays/sequencing versus polymerase chain reaction (PCR). The use of such preliminary gauges suffices to arrive at a first impression of the complex phenomenon under study. In line with the screening nature of the high‐throughput techniques, we do not aim to infer anything regarding a particular element of the precision matrix. Instead, we present a test that facilitates a more ‘global’ but still constructive type of inference regarding the Gaussian graphical model.

Our test assumes knowledge on the phenomenon under study to be available from related domains and evaluates this knowledge's informativeness to the current setting. The test thus assesses whether we can borrow, information or if we should start from a clean sheet. For instance, if we aim to reconstruct a pathway's regulatory network in a rare cancer subtype, knowledge from different and more prevalent and well‐studied subtypes of the same tissue may benefit our endeavor. We make this more precise and assume that two proposals, denoted as $T_{0},T_{a}\in S_{p}^{++}$, for the precision matrix Ω are available from related domains. The proposal $T_{0}$ represents the current belief in the precision matrix. This proposal may represent the absence of knowledge, e.g., through a diagonal matrix that harbors no conditional dependencies. The proposal $T_{a}$ is a competing one, e.g., derived from a different and more prevalent cancer subtype. This is illustrated in the application (Section 6), where we take $T_{a}$ to be the precision matrix estimate of a different subtype's gene–gene interaction network obtained from publicly available transcriptomic data. We dub the direction from $T_{0}$ to $T_{a}$ the *signpost*. Our test, accordingly called the *signpost test*, evaluates whether for the current setting the signpost points in the direction of the precision matrix's true value.

The paper is structured as follows. We first introduce the signpost test statistic, which is defined implicitly but can be evaluated numerically. We show that, under conditions, the signpost test statistic may be conceived as the step size on the line in the direction of the alternative precision matrix proposal. We describe how to generate the signpost test statistic's null distribution by a parametric bootstrap procedure. Alternatively, if bootstrapping is computationally demanding, we propose an approximate signpost test for which, under the null hypothesis, an asymptotic distribution is available. We investigate by simulation the type I error and power of the proposed (approximate) signpost test, under a variety of proposals, sample sizes, and dimensions. Moreover, we compare the signpost test with the likelihood ratio test. Finally, we consider the reconstruction of a pathway's gene–gene interactions in estrogen negative breast cancer samples. In this context, we decide by means of the signpost test on the informativeness of the estimated precision matrix from the more prevalent estrogen positive breast cancer samples. We substantiate the test's significance by showing *(i)* the empirical validity of the signpost test statistic as a metric of informativeness, *(ii)* a loss improvement in the direction of the estrogen positive target confirming the signpost test's significance, and *(iii)* how the signpost test's result may be exploited for network reconstruction.

## 2 Test Statistic

Suppose we have n independently and identically distributed draws $Y_{1},\text{…},Y_{n}\in R^{p}$ from the Gaussian graphical model $N(0_{p},\Omega^{-1})$ available. Let $T_{0}$ and $T_{a}$ be the null and alternative proposals for the precision matrix. We aim to test whether the data support the signpost, i.e., the direction from $T_{0}$ to $T_{a}$, to the true value of Ω. We assume that $\Omega =\left(1-\gamma \right)T_{0}+\gamma T_{a}$ with γ∈[0,1], a weighted average of two precision matrices. But, when rewritten as $\Omega =T_{0}+\gamma \left(T_{a}-T_{0}\right)$, it can be viewed as a point on the line segment that connects the precision matrices $T_{0}$ and $T_{a}$. In this view, γ quantifies how far Ω is from $T_{0}$ in the direction of the signpost. The signpost test evaluates the null hypothesis $H_{0}:\gamma =0$ against the alternative one $H_{a}:\gamma >0$. Rejection of $H_{0}$ does not imply that proposal $T_{a}$ is to be preferred over $T_{0}$, rather than a better value of the precision matrix can be found in the direction of $T_{a}$. Similarly, failure to reject does not imply that the precision matrix equals $T_{0}$, only that the data do not support the direction of $T_{a}$ in the search for a better parameter value.

Our test statistic development relies on the bi‐targeted ridge precision matrix estimator (van Wieringen et al. 2020). This estimator maximizes the loglikelihood augmented with a ridge penalty centered at the weighted average of two different locations, called targets or proposals and denoted $T_{0}$ and $T_{a}$, of the parameter space $S_{p}^{++}$. Formally, we define this estimator as

$$\begin{array}{ccc} \hat{\Omega }\left(\lambda ,\theta \right) & = & \arg\max_{\Omega \in S_{p}^{++}}\;\;\log\left(|\Omega |\right)-\text{tr}\left(S\Omega \right)\\ & & -\frac{1}{2}\lambda {∥\Omega -\left(1-\theta \right)T_{0}-\theta T_{a}∥}_{F}^{2}, \end{array}$$

with sample covariance matrix $S=n^{-1}\sum_{i=1}^{n}Y_{i}Y_{i}^{⊤}$, penalty parameter λ>0 and weight parameter θ∈[0,1]. The estimator has an analytic expression:

$$\begin{array}{ccc} \hat{\Omega }\left(\lambda ,\theta \right) & = & \left(\frac{1}{2}\left[S-\lambda \left(1-\theta \right)T_{0}-\lambda \theta T_{a}\right]\right.\\ & & +{\left.{\left\{\lambda I_{pp}+\frac{1}{4}{\left[S-\lambda \left(1-\theta \right)T_{0}-\lambda \theta T_{a}\right]}^{2}\right\}}^{1/2}\right)}^{-1}, \end{array}$$

see van Wieringen and Peeters (2016) and van Wieringen et al. (2020) for its derivation. Both λ and θ are tuning parameters, for instance chosen through cross‐validation. Simulations in van Wieringen et al. (2020), that take either of the two targets to equal the true Ω, indicate that the cross‐validated θ puts most weight on the true target. Hence, θ is potentially a good metric to decide between the two proposals.

We take the signpost test statistic to be the θ that yields the best fit as measured by the loglikelihood. Formally,

$$\begin{array}{ccc} \tilde{\theta }\left(\lambda \right) & = & \arg\max_{\theta \in [0,1]}\;\;\log[|\hat{\Omega }\left(\lambda ,\theta \right)\left|\right]-\text{tr}\left[S\hat{\Omega }\left(\lambda ,\theta \right)\right]. \end{array}$$

We use this test statistic to decide between $T_{0}$ and $T_{a}$. It involves the penalty parameter, and its influence on the test's power is investigated later (see Section 5).

We prove that $\hat{\theta }\left(\lambda \right)$ is in some settings uniquely defined (the proof is, as are all proofs, deferred to the Supporting information, henceforth abbreviated to SM). In simulations, we explored other settings that indicate that the function maximized by $\hat{\theta }\left(\lambda \right)$ is concave. A possible non‐uniqueness is circumvented by choosing the smallest maximizing $\hat{\theta }\left(\lambda \right)$, which may render the signpost test to be conservative. The test statistic's definition in the display above can be straightforwardly evaluated by standard root finding procedures, which we initiate by a value close to zero to arrive at the smallest maximizing $\hat{\theta }\left(\lambda \right)$. Numerical experiments indicate that only for small λ the test statistic may differ noticeably from ${\hat{\theta }}_{\infty }$ (see SM).

In the remainder, we focus mostly on the test statistic that corresponds to λ→∞‐limit and we denote by ${\hat{\theta }}_{\infty }$. This ${\hat{\theta }}_{\infty }$ is the limit of the test statistic definition, and the asymptote of $\hat{\theta }\left(\lambda \right)$ as λ tends to infinity. For finite but not too large λ, the difference between $\hat{\theta }\left(\lambda \right)$ and ${\hat{\theta }}_{\infty }$ is small and the latter is a good approximation of the former. Practically, the use of ${\hat{\theta }}_{\infty }$ avoids having to choose λ. Moreover, it makes the choice of the penalty, e.g., ridge or lasso, irrelevant. More importantly, within the context of ridge regression, we showed that this choice yields the most powerful test (van Wieringen 2024). This observation can be understood within the Bayesian framework. There, priors instead of penalties are used, but the Bayesian maximum a posteriori (MAP) estimator coincides with its penalized counterpart. This equivalence motivates the adoption of the Bayesian view to understand the power improvement of the signpost test with the ${\hat{\theta }}_{\infty }$ test statistic. To see this, we contrast vague to concentrated priors. The former, corresponding to a small λ, result in overlapping priors that are hard to distinguish with respect to the corresponding, almost identical MAP estimators. The adopted choice of a large λ, on the other hand, corresponds to concentrated priors that yield clearly different MAP estimators and facilitate discrimination between the underlying priors. To formally define our test statistic, note that in the regularization limit, $\lim_{\lambda \to \infty }\hat{\Omega }\left(\lambda \right)=\left(1-\theta \right)T_{0}+\theta T_{a}$. Substitute this in the loglikelihood and choose ${\hat{\theta }}_{\infty }$ as its maximizer:

$$\begin{array}{ccc} {\hat{\theta }}_{\infty }\; & = & \;\arg\max_{\theta \in [0,1]}\log[|T_{0}+\theta \left(T_{a}\;-T_{0}\right)\left|\right]-\text{tr}\left[T_{0}S+\theta \left(T_{a}-T_{0}\right)S\right]. \end{array}$$

The ${\hat{\theta }}_{\infty }$ is uniquely defined as it is the maximizer of a concave function, which follows from the negative sign of the function's second‐order derivative with respect to θ. To find the ${\hat{\theta }}_{\infty }$, take the derivative of the loss function w.r.t. θ and equate it to zero:

(1) $$\begin{array}{ccc} \text{tr}[\left(T_{a}-T_{0}\right)S] & = & \text{tr}\{\left(T_{a}-T_{0}\right){\left[T_{0}+\theta \left(T_{a}-T_{0}\right)\right]}^{-1}\}. \end{array}$$

The root of this equation is our test statistic ${\hat{\theta }}_{\infty }$.

### 2.1 Computationally Efficient Evaluation

In the absence of an analytic expression of the test statistic ${\hat{\theta }}_{\infty }$, we resort to numerical means for its evaluation. To that end it is computationally most efficient to reformulate Equation (1) to:

$$\begin{array}{ccc} p-\theta \sum_{j,j^{\prime }=1}^{p}{\left[\left(T_{a}-T_{0}\right)\circ S\right]}_{j,j^{\prime }} & = & \sum_{j=1}^{p}{\left[\left(1-\theta \right)+\theta d_{j,a/0}\right]}^{-1}, \end{array}$$

where ∘ is the Hadamard matrix product and $d_{j,a/0}$ is the jth eigenvalue of $T_{0}^{-1/2}T_{a}T_{0}^{-1/2}$ in which $A^{1/2}$ denotes the matrix square root. Then, to avoid any matrix operation inside the search procedure, evaluate $\sum_{j,j^{\prime }=1}^{p}{\left[\left(T_{a}-T_{0}\right)\circ S\right]}_{j,j^{\prime }}$ and the eigenvalues of $T_{0}^{-1/2}T_{a}T_{0}^{-1/2}$ prior to the initiation of the search. Note in our computationally efficient reformulation of the test statistic equation, we have introduced an additional root, θ=0, which is to be ignored, unless its multiplicity exceeds one.

The search procedure for the test statistic can be sped up if we provide a warm start and are able to bound the search space. The warm start is discussed in Section 4 and Proposition 2.1 provides the bounds.

Proposition 2.1 Let $d_{1,a/0}$ and $d_{p,a/0}$ denote the largest and smallest, respectively, eigenvalue of $T_{0}^{-1/2}T_{a}T_{0}^{-1/2}$. Assume θ∈[0,1], $\text{tr}[\left(T_{a}-T_{0}\right)S]\neq 0$, and $d_{1,a/0}\neq 1=d_{p,a/0}$. Then, the root of Equation (1) satisfies:

i)
$\theta \;\leq \;\left\{p-\text{tr}\left[T_{0}{\left(T_{0}+T_{a}\right)}^{-1}\right]\right\}/\text{tr}\left[\left(T_{a}-T_{0}\right)S\right]$,
ii)
$\theta \;\leq \;p/\text{tr}\left[\left(T_{a}-T_{0}\right)S\right]-{\left(d_{1,a/0}-1\right)}^{-1}$,
iii)
$\theta \;\geq \;p/\text{tr}\left[\left(T_{a}-T_{0}\right)S\right]-{\left(d_{p,a/0}-1\right)}^{-1}$,
iv)
$\theta \;<\;\left\{p-{\left[\text{tr}\left(T_{0}^{-1/2}T_{a}T_{0}^{-1/2}\right)\right]}^{-1}\right\}{\left\{p^{-1}-{\left[\text{tr}\left(T_{0}^{-1/2}T_{a}T_{0}^{-1/2}\right)\right]}^{-1}+\text{tr}\left[\left(T_{a}-T_{0}\right)S\right]\right\}}^{-1}$.

### 2.2 Unbiasedness

The construction of the null distribution of our test hinges upon the following proposition, which states that—under conditions—the test statistic ${\hat{\theta }}_{\infty }$ can be viewed as an approximate and asymptotically unbiased estimator of γ. Importantly, the test statistic can thereby inherits the interpretation of a step size, it measures how far to follow the direction of the signpost in the search for the true precision matrix.

Proposition 2.2 Assume $\Omega =\left(1-\gamma \right)T_{0}+\gamma T_{a}$ with γ∈[0,1]. Then,

i)
$E({\hat{\theta }}_{\infty })=\gamma$ if γ=0.
ii)
${\hat{\theta }}_{\infty }$ is an asymptotically unbiased estimator of γ∈[0,1].
iii)
if in addition $1-\delta {\left(1+\delta \gamma \right)}^{-1}<d_{a/0,j}<1+\delta {\left(1-\delta \gamma \right)}^{-1}$ for j=1,…,p and $\delta =O(p^{-1/3})$, ${\hat{\theta }}_{\infty }$ is unbiased for γ∈[0,1] in the high‐dimensional limit p→∞.

The proof of Proposition 2.2 also reveals that, when approximated by a Taylor series, ${\hat{\theta }}_{\infty }$ is unbiased for γ up to second and higher order terms. The latter terms vanish asymptotically and, under conditions, as the number of dimensions grows large. Together, these findings suggest that ${\hat{\theta }}_{\infty }$ is close to unbiased for γ on the whole unit interval for finite sample sizes and dimensions.

**FIGURE 1:**
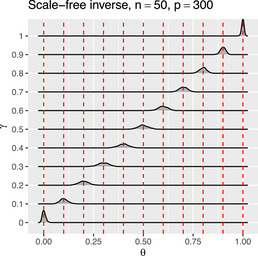
Kernel density estimated of estimated ${\hat{\theta }}_{\infty }$ against γ.

Simulation evidence indicates that ${\hat{\theta }}_{\infty }$ is also a virtually unbiased estimator of step size γ for finite sample sizes and dimensions (see SM). This is illustrated by Figure 1, which results from a small simulation study. It comprises drawing from $Y∼N\{0_{p},{\left[\left(1-\gamma \right)T_{0}+\gamma T_{a}\right]}^{-1}\}$ with n=50, p=300, γ∈{0,0.1,0.2,…,1}, and targets $T_{0}=I_{pp}$ and $T_{a}$ a scale‐free inverse (details in the SM), respectively. From each draw, we evaluate ${\hat{\theta }}_{\infty }$. For each choice of γ and $T_{a}$, this is repeated a thousand times. The resulting ${\hat{\theta }}_{\infty }$ are plotted against γ (see Figure 1 and the SM). The plot reveals that the ${\hat{\theta }}_{\infty }$’s are distributed along the x=y‐line with their mode at the corresponding γ value. Hence, also for finite sample sizes, the test statistic ${\hat{\theta }}_{\infty }$ is indicative of the true precision matrix's position on the line between the targets.

The assumption of Proposition 2.2 that the true parameter Ω is on the line between the null and alternative target is stringent, but this assumption is exactly what underpins the signpost test. We will therefore investigate the signpost test's robustness against violations of this assumption (see Section 5).

## 3 Signpost Testing

The signpost test evaluates whether there is relevant information in the direction from the null to the alternative target with the parameter of the Gaussian graphical model. This direction is parameterized by the line segment $\left(1-\gamma \right)T_{0}+\gamma T_{a}$ for γ∈[0,1]. The signpost test assesses whether the model parameter is on this line. It does so by testing $H_{0}:\gamma =0$ vs. $H_{a}:\gamma >0$. Rejection of $H_{0}$ indicates that there is relevant information with respect to the true model parameter to be found in the direction from the null to the alternative target. Failure to reject the null hypothesis does not imply that $T_{0}$ is the location of the model parameter, for that may well be found in alternative directions than that of $T_{a}$.

The signpost test of the Gaussian graphical model uses ${\hat{\theta }}_{\infty }$, or $\hat{\theta }\left(\lambda \right)$, as test statistic. It is, under the $\Omega =\left(1-\gamma \right)T_{0}+\gamma T_{a}$ assumption and by Proposition 2.2, a good metric to quantify the amount of relevant information regarding the location of the true parameter. The signpost test compares the observed test statistic to its null distribution generated by the parametric bootstrap. Formally, the outline of the signpost test procedure is:

*Signpost test of the Gaussian graphical model*. To test $H_{0}:\gamma =\gamma_{0}$ vs. $H_{a}:\gamma \neq \gamma_{0}$:

1)
Evaluate the test statistic from observed data and
  denote it ${\hat{\theta }}_{\infty }^{H_{0},\left(0\right)}$ and, for later use, ${\hat{\theta }}_{\infty }^{\text{obs}}$.
2)
To generate the null distribution, iterate the following steps K times:
i)
Sample null data $Y_{1}^{H_{0},\left(k\right)},\text{…},Y_{n}^{H_{0},\left(k\right)}∼N\left(0_{p},T_{0}^{-1}\right)$ for
    the k‐th iteration with k=1,…,K.
ii)
Evaluate ${\hat{\theta }}_{\infty }$ using the null data. Denote this result by ${\hat{\theta }}_{\infty }^{H_{0},\left(k\right)}$
    for the k‐th iteration with k=1,…,K.
3)
Calculate the p‐value by
$$\begin{array}{ccc} & & 2\min\{{\left(K+1\right)}^{-1}\left[1+\sum_{k=0}^{K}1_{\left\{{\hat{\theta }}_{\infty }^{H_{0},\left(k\right)}\geq {\hat{\theta }}_{\infty }^{H_{0},\left(0\right)}\right\}}\right],\\ & & 2\min\{{\left(K+1\right)}^{-1}\left[1+\sum_{k=0}^{K}1_{\left\{{\hat{\theta }}_{\infty }^{H_{0},\left(k\right)}\leq {\hat{\theta }}_{\infty }^{H_{0},\left(0\right)}\right\}}\right]\}, \end{array}$$

The p‐value of the signpost test may be accompanied by a more subtle uncertainty quantification through the construction of an approximate confidence interval. This interval is obtained by similar means as the signpost test's null distribution. In the second step of the signpost test procedure, we sample from $N\{0_{p},{\left[\left(1-{\hat{\theta }}_{\infty }^{\text{obs}}\right)T_{0}+{\hat{\theta }}_{\infty }^{\text{obs}}T_{a}\right]}^{-1}\}$. Then, by virtue of the approximately unbiasedness of ${\hat{\theta }}_{\infty }$ for γ (Proposition 2.2), for α∈(0,1) the $\frac{1}{2}\alpha$ and $1-\frac{1}{2}\alpha$ quantiles of the resulting distribution form an approximate 100(1−α) confidence interval.

## 4 An Approximate Signpost Test

Here, we present an alternative test based on an approximate signpost test statistic for which the asymptotic distribution is known. The corresponding exceedance probability can then directly be calculated. This circumvents the potentially computationally demanding generation of the test statistic's null distribution of the original signpost test introduced in Section 2.

We approximate the test statistic of the signpost test. To this end, we linearize the right‐hand side of the test statistic equation (1):

$$\begin{array}{ccc} \text{tr}[\left(T_{a}-T_{0}\right)S] & \approx & \text{tr}\left(T_{0}^{-1/2}T_{a}T_{0}^{-1/2}\right)-p-\theta {∥T_{0}^{-1/2}T_{a}T_{0}^{-1/2}-I_{pp}∥}_{F}^{2}. \end{array}$$

The root of this resulting equation,

$$\begin{array}{ccc} {\tilde{\theta }}_{\infty } & = & \{\text{tr}\left(T_{0}^{-1/2}T_{a}T_{0}^{-1/2}\right)-p\\ & & -\text{tr}\left[\left(T_{a}-T_{0}\right)S\right]\}\;∥T_{0}^{-1/2}T_{a}T_{0}^{-1/2}-I_{pp}{∥}_{F}^{-2}, \end{array}$$

serves as an approximate test statistic.

The approximate test statistic ${\tilde{\theta }}_{\infty }$ also uses evidence present in the data to measure how far the true precision matrix is from the null target matrix, in the direction of the alternative target. This is expressed by the following proposition.

Proposition 4.1 If $\Omega =\left(1-\gamma \right)T_{0}+\gamma T_{a}$, then

$$\begin{array}{ccc} \gamma^{2}\left(1-d_{a/0,1}\right){\left(1-\gamma +\gamma d_{a/0,1}\right)}^{-1} & \leq & E\left({\tilde{\theta }}_{\infty }\right)-\gamma \leq \gamma^{2}\left(1-d_{a/0,p}\right)\\ & & \times \;{\left(1-\gamma +\gamma d_{a/0,p}\right)}^{-1}. \end{array}$$

The interval bounding the bias of the approximate test statistic as an estimator of γ provided by Proposition 4.1 contains, under the mild assumption of $d_{a/0,1}>1$ and $d_{a/0,p}<1$, zero. This interval grows for larger γ. But for γ close to zero, the approximate test statistic is almost unbiased. In particular, if γ=0, the approximate test statistic is an unbiased estimator of γ. This suggests that the approximate test statistic ${\tilde{\theta }}_{\infty }$ is sensitive enough to evaluate $H_{0}:\gamma =0$ versus $H_{a}:\gamma >0$.

The SM provides an impression of the quality of ${\tilde{\theta }}_{\infty }$ as an approximation of ${\hat{\theta }}_{\infty }$. This impression recycles the simulation described in Section 2.2 but now calculates the original and approximate test statistics for each iteration. These are plotted against each other in a *qq*‐plot (see the SM). It reveals, as suggested by Proposition 4.1, that the approximation is good for small γ close to zero but becomes poorer for larger γ. Nonetheless, this shows the suitability of the approximate test statistic for testing $H_{0}:\gamma =0$ versus $H_{a}:\gamma >0$.

The null distribution of the approximate signpost test statistic is known asymptotically (cf. Corollary 4.2) as *(i)* ${\tilde{\theta }}_{\infty }\propto \text{tr}\left[\left(T_{a}-T_{0}\right)S\right]$ and *(ii)* the asymptotic distribution of this trace has been characterized (Fujikoshi 1970).

Corollary 4.2 Let nS follow the central Wishart distribution $W_{p}\left(n,\Sigma \right)$ and $T_{a}-T_{0}$ be nonsingular. The asymptotic distribution of ${\tilde{\theta }}_{\infty }$ is

$$\begin{array}{ccc} & & P[\sqrt{n}\sigma^{-1}\left({\tilde{\theta }}_{\infty }-\theta_{\infty }\right)<z]\\ & & \;=\Phi \left(z\right)-\frac{4}{3\sqrt{n}\sigma^{3}}\text{tr}\left[{\left(R^{-1}\Sigma \right)}^{3}\right]\Phi^{\left(3\right)}\left(z\right)\\ & & \;+\;\frac{2}{n\sigma^{4}}\{\text{tr}\left[{\left(R^{-1}\Sigma \right)}^{4}\right]\Phi^{\left(4\right)}\left(z\right)+\frac{4}{9\sigma^{2}}{\left\{\text{tr}\left[{\left(R^{-1}\Sigma \right)}^{3}\right]\right\}}^{2}\Phi^{\left(6\right)}\left(z\right)\}\\ & & \;\;-\;\frac{8}{n\sqrt{n}\sigma^{5}}\{\frac{2}{5}\text{tr}\left[{\left(R^{-1}\Sigma \right)}^{5}\right]\Phi^{\left(5\right)}\left(z\right)+\frac{1}{3\sigma^{3}}\\ & & \;\times \;\text{tr}\left[{\left(R^{-1}\Sigma \right)}^{3}\right]\text{tr}\left[{\left(R^{-1}\Sigma \right)}^{4}\right]\Phi^{\left(7\right)}\left(z\right)\\ & & \;+\;\frac{4}{81\sigma^{4}}{\left\{\text{tr}\left[{\left(R^{-1}\Sigma \right)}^{3}\right]\right\}}^{3}\Phi^{\left(9\right)}\left(z\right)\}+O\left(n^{-2}\right), \end{array}$$

where $\sigma =\sqrt{2\text{tr}[{\left(R^{-1}\Sigma \right)}^{2}]}$, $b=∥T_{0}^{-1/2}T_{a}T_{0}^{-1/2}-I_{pp}{∥}_{F}^{2}$, $R^{-1}=b^{-1}\left(T_{0}-T_{a}\right)$, $\theta_{\infty }$ defined as ${\tilde{\theta }}_{\infty }$ with S replaced by Σ, and $\Phi^{\left(r\right)}\left(z\right)$ denotes the rth derivative of the standard normal distribution function Φ(z).

This is a direct consequence of Theorem 11.2 of Fujikoshi (1970), which for completeness is quoted in the SM.

Knowledge of the approximate test statistic's asymptotic distribution facilitates a parametric test. In low dimensions, under the null hypothesis $H_{0}:\gamma =0$, and with a reasonable sample size, the asymptotic distribution of Corollary 4.2 is expected to be a good approximation to the null distribution. Simulation evidence presented in Section 5 indicates that even high‐dimensionally this parametric test has comparable power (see SM) to the test presented in Section 3.

## 5 Test Performance

We evaluate the power of the proposed signpost tests with test statistics $\hat{\theta }\left(\lambda \right)$, ${\hat{\theta }}_{\infty }$ and ${\tilde{\theta }}_{\infty }$ in simulation. We do so under various choices of sample sizes, dimensions, effect sizes and conditional dependence graphs. Moreover, we assess the signpost tests' robustness against violation of the key assumption that the true parameter is a weighted average of the null and alternative proposals.

We first specify the simulations details. We draw data $Y_{1},\text{…},Y_{n}$ from the Gaussian graphical model $N(0_{p},\Omega^{-1})$. The sample size n and dimension p is chosen from the set {10,20,…,100} and {50,150}, respectively. The precision matrix Ω is a weighted average of the null and alternative proposal, i.e., $\Omega =\left(1-\gamma \right)T_{0}+\gamma T_{a}$. The weight parameter γ equals a value from {0,0.1,0.2,…,1}. Throughout we set $T_{0}=I_{pp}$ and employ various $T_{a}$. All employed $T_{a}$ have a unit diagonal and differ in their off‐diagonal structure. These structures represent the topology of the conditional independence graphs: uniform (saturated), banded, stripe (two hubs), and scale‐free (see the SM for details). With generated data, we use the signpost test with a thousand parametric bootstraps to evaluate the null hypothesis $H_{0}:\gamma =0$ vs. the alternative $H_{a}:\gamma >0$. The signpost test is declared significant if the p‐value exceeds the significance level α=0.05. We repeat the above a thousand times for all $\left(n,p,\gamma ,T_{a}\right)$‐combinations. Per such combination, we calculate the proportion of rejected null hypothesis.

Before we report the signpost test's power, we note that the type I error is well‐controlled by construction. For, under the null hypothesis, both the observed and bootstrapped test statistics are obtained from data drawn from the same distribution. Superfluously, we confirmed in simulation that the histogram (not shown) of the signpost test's p‐values is indeed uniform under $H_{0}$.

The power curves (see SM) of the signpost test equipped with the ${\hat{\theta }}_{\infty }$ test statistic show an improvement in the signpost test's power with an increase in either sample size and/or effect size. Both are properties of a useful test, as larger sample sizes enable a better inference and a larger effect is easier to detect.

The ${\hat{\theta }}_{\infty }$ test statistic equipped signpost test's power profits from a larger dimension of the precision matrix. The gain in power is consistent over sample sizes, effect sizes, and topologies. This gain can intuitively be understood in two ways.

i)
The number of elements that differ between the null and alternative targets increases with dimension. More differing elements are more locations that exhibit the effect of a nonzero γ. These enhance the signpost test's capacity to detect a nonzero γ. This is supported by the differences of the power curves among the simulations with the various employed topologies of the alternative target. For instance, compare the situations where $T_{a}$ has a chain or banded conditional independence graph. The case involving the latter has two extra off‐diagonals with nonzero elements and its power curves are systematically above the case with the former.
ii)
The (absolute) size of the differences among elements of the null and alternative target affects the gain in power. This is due to the fact that the size and γ enter the target multiplicatively. Hence, a larger difference among the elements of $T_{0}$ and $T_{a}$ amplifies the effect of γ, making it easier for the signpost test to pick it up.

Practically, the power curves enable us to deduce a rough sample size indication for the study. For instance, would we want to detect a step size γ=0.20 to distinguish between two 150×150‐dimensional precision matrices with an empty and scale‐free topology, we require at least n=50 samples to do so with 90% power. This sample size is heavily dependent on the assumed topologies of the precision matrices' conditional independence graphs and the difference between the precision matrices' elements. Irrespective of the exact sample size, it tells us that a limited sample size is sufficient to detect the relevance of a proposed direction in a high‐dimensional parameter space.

The power curves of signpost test equipped with the approximate test statistic ${\tilde{\theta }}_{\infty }$ using its asymptotic distribution as an approximation to its null distribution to calculate the p‐values are depicted in the SM. This version of the signpost test exhibits comparable—if not equal—power to the one equipped with the ${\hat{\theta }}_{\infty }$ test statistic and the parametric bootstrap generated null distribution. This is understandable as the approximate test statistic ${\tilde{\theta }}_{\infty }$ is an unbiased estimator of γ=0 (Proposition 4.1) and, thereby, an appropriate summary of the evidence against $H_{0}:\gamma =0$.

We now study the power of the signpost test with the test statistic $\hat{\theta }\left(\lambda \right)$ as defined in Display (1). We adopt the same simulation set‐up as described above and use the modified test statistic with λ=10,100,1000. The resulting power curves for a scale‐free alternative target (with power curves representative for all alternative target types) are shown in the SM. These curves reveal that the signpost test equipped with the finite λ test statistic $\hat{\theta }\left(\lambda \right)$ has slightly less power than its counterpart with the ${\hat{\theta }}_{\infty }$ test statistic. Hence, the signpost test with the ${\hat{\theta }}_{\infty }$ test statistic is to be preferred as it yields a better power. We believe this to be rooted in the minimum variance of the bi‐targeted ridge precision estimator at λ=∞. Irrespectively, it conveniently avoids the issue of making an informed choice on λ.

So far we have investigated the signpost test's behavior under the assumption that the precision matrix Ω is on the line between the two proposals $T_{0}$ and $T_{a}$. This assumption is rather stringent. We therefore provide some insight on the signpost test's behavior under violation of this assumption. We take Ω as a perturbed version of $T_{a}$ (or vice versa). The perturbation amounts to a rotation of (part of) the eigenspace but leaves the eigenvalues unaffected. Technical details of the perturbation are deferred to the SM. The rotation adheres to the signpost test's motivation as the test evaluates a direction. Moreover, the rotation ensures the positive‐definite perturbed target/precision matrix. We then repeat the power simulations with identical settings as before but with a precision matrix that does not adhere to the key assumption. In these simulations, we vary the severity of the violation of the assumption through different ‘angles’ of the rotation.

The plots showing the signpost test's power under the aforementioned misspecification of its underpinning assumption $\Omega =\left(1-\gamma \right)T_{0}+\gamma T_{a}$ can be found in the SM. Their main takeaway is that the signpost test still has considerable power under (mild) misspecification of the alternative proposal. Put differently, the signpost test can still detect (with some power) whether it is worthwhile to pursue the direction of its signpost, even though the true parameter is not on the line from the null to the alternative target.

### 5.1 Comparison

We compare the signpost test with the likelihood ratio test. The latter is a natural competitor of the signpost test as the null hypothesis (γ=0) is nested in the alternative (γ∈(0,1]). For λ=∞, the statistics of these tests are one‐to‐one related, as that of the likelihood ratio test is $\max_{\theta \in [0,1]}2\left\{L\left[S,\left(1-\theta \right)T_{0}+\theta \right)T_{a}\right]-L\left(S,T_{0}\right)\}$. A different test statistic brings about a different null distribution, i.e., a mixture of a point mass at zero and $\chi_{1}^{2}$ distribution asymptotically for the likelihood ratio test statistic (Self and Liang 1987). This may affect the p‐value calculation and inference. Both test statistics extend naturally into the finite λ domain, but there the quality of the asymptotic approximation of the likelihood ratio test's null distribution is unknown. The comparison between the two tests adopts the same set‐up as that for the type I error and power evaluation of Section 5. The resulting power curves can be found in the SM. They convey the following messages. The type I error of the likelihood ratio test is inflated. This questions the suitability of the asymptotic $\chi_{1}^{2}$ distribution as null distribution. Furthermore, the likelihood ratio test is on a par with the signpost test in terms of power.

An additional comparison involves the signpost test that employs the approximated signpost test statistic and its asymptotic distribution. Its type I error is well‐controlled, while its power curves overlay with that of the signpost test with a bootstrapped null distribution.

## 6 Application

We show how to employ the signpost test in practice through a re‐analysis of six data sets from transcriptomic breast cancer studies. These datasets have been curated and formatted in identical fashion and are publicly available from the Bioconductor platform (https://bioconductor.org/) through the breastCancer*** packages (Schroeder et al. 2022). Each data set includes the expression levels of the same 22283 transcripts. These transcripts are subsetted to 120 transcripts that map to the ‘breast cancer ‐ homo sapiens (human)’ pathway as defined by the KEGG‐repository (Ogata et al. 1999) with KEGG‐ID: hsa05224. To adhere to the distributional assumptions of the Gaussian graphical model, the data are Gaussianized marginally, which does not affect the underlying conditional independence graph (Liu et al. 2009). Furthermore, each dataset comprises samples with estrogen receptor negative and positive status, denoted ER− and ER+, indicating the absence and presence, respectively, of the estrogen hormone, which is a pivotal driver in breast cancer (Foulkes et al. 2010). The ER− samples are less prevalent (see Table 1) and will be the focus of our re‐analysis. The re‐analysis of these data by means of the signpost test's methodology aims to illustrate how the information of the ER+ samples can be utilized in the learning of the pathway's regulatory network (as operationalized by the conditional independence graph of the Gaussian graphical model) from the ER− samples.

**TABLE 1:** A short label for each dataset derived from the R‐package name with the corresponding data, its number of ER− and ER+ samples, and results (test statistic and p‐value) of the signpost test of the breast cancer pathway in the ER‐ samples. The p‐values are evaluated from a thousand bootstraps.

Dataset	#ER−	#ER+	${\hat{\theta }}_{\infty }$	p‐Value		Dataset	#ER−	#ER+	${\hat{\theta }}_{\infty }$	p‐Value
MAINZ	38	162	0.300	≤0.001		UNT	40	86	0.656	≤0.001
NKI	51	187	0.487	≤0.001		UPP	34	213	0.564	≤0.001
TRANSBIG	64	134	0.201	≤0.001		VDX	135	209	0.609	≤0.001

### 6.1 Empirical Validity

First, we assess, as shown in simulation and by mathematical analysis in preceding sections, the empirical substantiation of the test statistic as a metric for detection of the relevance of a signpost. This requires knowledge of the truth, which is typically absent for the data at hand. We may nonetheless construct a situation that reveals the validity of the proposed metric of the signpost's relevance through its behavior.

We require informative targets for the ER− and ER+ groups that are independent of the data used to evaluate the informativeness of the signpost. This ensures that the data are not used twice. To this end, we sacrifice (say) the bth breast cancer data set. This dataset is only used for target construction. The resulting targets are then employed in the signpost test with another datasets. As such, the hypothesis, which involves the constructed targets, and the data used for its evaluation are independent. From the data of the bth dataset, we form the ER− and ER+ targets through ridge penalized precision matrix estimation with a leave‐one‐out cross‐validated penalty parameter (van Wieringen and Peeters 2016). We denote these targets $T_{\text{er-},b}$ and $T_{\text{er+},b}$ for b=1,…,B. In the SM, we discuss other methods of target construction and investigate their effect.

To assess the signpost test statistic's validity, we assume the ER− and ER+ targets are formed from the $b^{\prime }$th breast cancer dataset and consider the bth breast cancer dataset with $b\neq b^{\prime }$ to construct a sequence of $n_{\text{er-},b}+1$ datasets of $n_{\text{er-},b}$ samples. The first constructed dataset comprises only the $n_{\text{er-},b}$ ER− samples of this dataset. Each subsequent dataset is formed (details in the SM) by randomly replacing an ER− sample by a randomly selected ER+ sample from the same dataset. At the beginning and end of this sequence of datasets, we expect, respectively, $T_{\text{er-},b^{\prime }}$ and $T_{\text{er+},b^{\prime }}$ to be more informative. In particular, if we run through this sequence from the first to the last dataset, any valid metric of signpost's relevance would increase monotonically thereby expressing an increased preference for the ER+ target.

We evaluate the signpost test statistic ${\hat{\theta }}_{\infty }$ for the afore‐described sequence of datasets with an increasing ratio of ER+ to ER− samples. This is done for all possible $\left(b,b^{\prime }\right)$ dataset pairs, with $b^{\prime }$ used for target formation and b for test statistic evaluation. We repeat this exercise 50 times to reduce the randomness in the construction of the dataset sequences as the ER− samples are replaced randomly by ER+ samples. Furthermore, to investigate the dependence of the target formation on the datasets' particulars, we also consider targets sampled from the convex hull of the, e.g., ${\left\{T_{\text{er+},b}\right\}}_{b=1}^{B}$. This gives ER+ targets $T_{\text{er+,all}}=\sum_{b=1}^{B}w_{b}T_{\text{er+},b}$ with the $w_{b}>0$ randomly sampled from the B‐simplex to ensure that $\sum_{k=1}^{K}w_{b}=1$.

**FIGURE 2:**
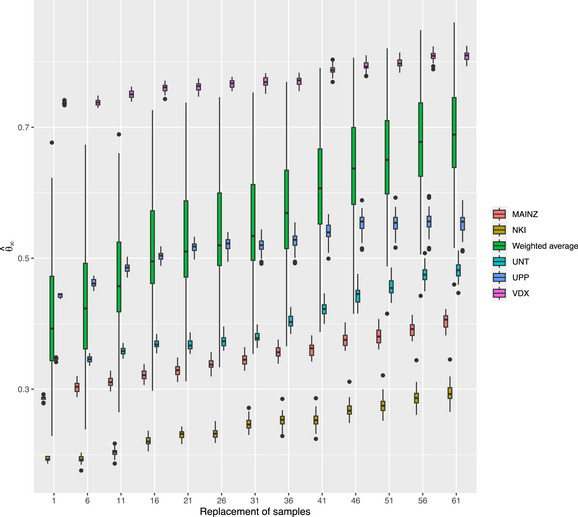
Boxplots of the ${\hat{\theta }}_{\infty }$ test statistic vs. the sequence of the datasets. The color of the boxplots refers to the dataset involved in the targets' formation. The labels of the boxplots in the legend refer to the names of the breast cancer datasets.

The estimated signpost test statistics of all aforementioned settings are plotted against the dataset order (Figure 2). The estimated signpost test statistics exhibit positive concordance with the order of the dataset sequence. This behavior is systematic over all considered settings, although concordance strengths vary, which—apart from inherent particulars of the lab and population—stem from difference in the sample size and ER+/ER− prevalence. The signpost test statistic is thus a valid metric of a signpost's relevance.

One may object to the concluded validity as there is no setting where the signpost test statistic runs from ${\hat{\theta }}_{\infty }=0$ to ${\hat{\theta }}_{\infty }=1$ over the order of the dataset sequence. This was not expected as the validity exercise hinges upon the strong assumption that the results from one breast cancer study are informative for the analysis of another. This informativeness is obscured by the inherent differences in their sampled population, lab protocols, measurement devices, and so on. Other reasons may explain the moderate differences of the ${\hat{\theta }}_{\infty }$’s at both ends of the dataset sequences. First, high‐throughput platforms are notoriously noisy and this noise may obscure the detection of the signpost's relevance. Second, the ER− and ER+'s Gaussian graphical models are not too different after all. Or, either the ER− or the ER+ group is heterogeneous, which too hampers the detection of the signpost's relevance. Hence, our focus on the concordance to empirically substantiate the signpost test statistic's validity.

### 6.2 Test and Fit

Within the context of the breast cancer studies, we now apply the signpost test to the archetypical case we encounter in practice. In that case, we have no knowledge on the Gaussian graphical model from the ER− samples. This is represented by the null target $T_{0}=I_{pp}$, which corresponds to an empty conditional independence graph and its unit diagonal matches with the variates' marginal variances after the Gaussianization. The alternative target is the Gaussian graphical model's precision matrix from a related population, the ER+ samples, which has been estimated by ridge penalized likelihood estimation with a cross‐validated penalty parameter and has been standardized to have a unit diagonal. We then apply the signpost test to each study's ER− samples for which we assess the relevance of an ER+ target.

The results of the signpost test, both the found test statistic ${\tilde{\theta }}_{\infty }$ and the p‐value (based on K=1000 redraws), are displayed in Table 1. In all datasets, the signpost test is significant at the α=0.05 level. We thus conclude that (temporarily) following the direction of the alternative target derived from the ER+ samples brings us closer to the value of the parameter of the ER− samples' Gaussian graphical model. The test statistic indicates the step size to be taken in that direction, which is substantial as all are ≥0.2. The results of the signpost test with the approximate test statistic ${\tilde{\theta }}_{\infty }$, provided in the SM, confirm the conclusion drawn from Table 1.

**FIGURE 3:**
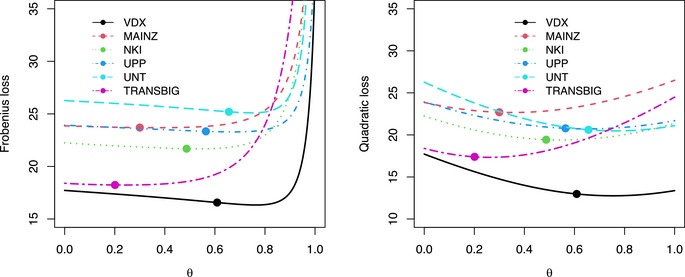
Diagnostic plots of the ‘line'‐assumption $\Omega =\left(1-\gamma \right)T_{0}+\gamma T_{a}$. Left panel: $T_{0}=I_{pp}$, $T_{a}={\hat{\Omega }}_{\text{er+, nki}}$, and Ω is replaced by the ridge precision estimate ${\hat{\Omega }}_{\text{er-, nki}}$. Right panel: $T_{0}={\hat{\Omega }}_{\text{er-,vdx}}$ and $T_{a}={\hat{\Omega }}_{\text{er+,vdx}}$ and $\Omega ={\hat{\Omega }}_{\text{er-,upp}}$.

We diagnose the validity of the principal assumption, i.e., the precision matrix' parameterization $\Omega =T_{0}+\theta \left(T_{a}-T_{0}\right)$, underlying the test for the breast cancer data. This assumption implies a linear relationship between the non‐redundant elements of $\Omega -T_{0}$ and those of $T_{a}-T_{0}$. We assess this linearity visually, with the unknown Ω replaced by an estimate. The use of a (regularized) estimate may partially obscure the linearity. We deem the principal assumption tenable if the plot shows a concordant relation that can roughly be approximated by a linear one. We generated this diagnostic plot for each performed signpost test. Two representative ones are shown in Figure 3. In one plot, the principal assumption is clearly unproblematic, while in the other the assumption is not invalid but certainly very noisy.

**FIGURE 4:**
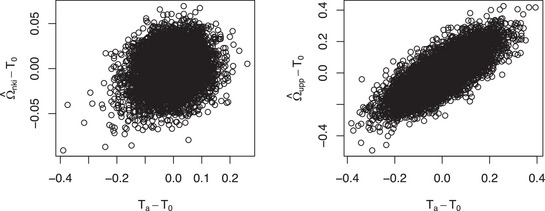
The Frobenius (left) and quadratic (right) loss of the bi‐targeted ridge precision matrix estimator against θ. The loss is plotted for all six breast cancer studies. The points on the curves correspond to the loss at the value of the test statistic ${\hat{\theta }}_{\infty }$.

A natural follow‐up question is to ask whether the model fit also improves in the proposed direction? To assess this, we exploit the interpretation of the test statistic as the position of the true precision matrix on the line segment connecting the targets. We evaluate whether the corresponding precision matrix, i.e., the average of the targets weighted by the test statistic, yields an improved fit. We therefore study the Frobenius and quadratic loss:

$$\begin{array}{ccc} L_{F}\left(\theta \right) & = & ∥S_{\text{er-}}-{\left[\left(1-\theta \right)I_{pp}+\theta T_{\text{er+}}\right]}^{-1}{∥}_{F},\\ L_{Q}\left(\theta \right) & = & ∥S_{\text{er-}}{\left[\left(1-\theta \right)I_{pp}+\theta T_{\text{er+}}\right]}^{-1}-I_{pp}{∥}_{F}, \end{array}$$

respectively. For each dataset, both losses are plotted against θ (see Figure 4). The curves are compared to the loss of the evaluated test statistic. A first takeaway from Figure 4 is that all curves decrease on the interval θ∈[0,a) for some a>0 clearly away from zero. This interval always includes $\theta ={\hat{\theta }}_{\infty }$. Moreover, the loss is generally minimized not at but certainly close to $\theta ={\hat{\theta }}_{\infty }$. This suggests that the test statistics, when interpreted as the informativeness of the direction of the alternative target, are conservative. In all, these findings corroborate the signpost test's result that the data support the proposed direction from $T_{0}$ to $T_{\text{er+}}$ to find the parameter of the ER− samples’ Gaussian graphical model.

### 6.3 Further Analyses

The SM contains additional analyses of the breast cancer data. These

i)
provide further down‐stream analyses of the pathway's reconstructed conditional independence graph.
ii)
employ differently constructed targets to investigate their effect on the signpost test's inference.

## 7 Conclusion

We presented a signpost test to detect whether valuable information regarding the Gaussian graphical model parameter is to be found in the direction of a suggested value. The test has good power to do so with limited sample size, and even benefits from larger dimensions. Moreover, the signpost test still has reasonable power if the true direction deviated from the suggested one. We illustrated the signpost test's practical use in a breast cancer application, where the reconstruction of the gene–gene interaction network of a lesser prevalent subgroup benefited from an estimate of the more prevalent one.

We envision that the signpost test will also find its use in federated learning. For privacy reasons, the EU's General Data Protection Regulation (GDPR) limits the exchange of data between medical institutions. But these medical institutions are allowed to exchange summary statistics, e.g., parameter estimates, of the data. These summary statistics serve as the external knowledge evaluated in the signpost test. For instance, this external knowledge may originate from a different country with a different population. Or, summary statistics may be exchanged between a general hospital and a cancer clinic. Both medical institutions treat cancer patients, but the latter typically receives the more severe cases. In either case, the signpost test assesses the informativeness of the external knowledge for the in‐house learning problem. This we illustrate in the SM.

Envisioned future work would extend this work in two directions. A first extension allows for the incorporation of more than one alternative target/proposal. The signpost test then evaluates whether the precision matrix lies in a hyperplane spanned by parameter values suggested by multiple external knowledge domains. Another line of future research would venture deeper into the realm of graphical models. For instance, vector auto‐regressive models do more justice to the dynamics of the cellular regulatory system.

## Conflicts of Interest

The authors declare no conflict of interest.

## Open Research Badges

This article has earned an Open Data badge for making publicly available the digitally‐shareable data necessary to reproduce the reported results. The data is available in the Supporting Information section.

This article has earned an open data badge “**Reproducible Research**” for making publicly available the code necessary to reproduce the reported results. “The results reported in this article could fully be reproduced.”

## Supporting information


**Supporting File:** bimj70115‐sup‐0001‐Datacode.zip.

## Data Availability

The data that supports the findings of this study are available in the supplementary material of this article.

## 1

This work is accompanied by online Supporting information, available from the journal's website, that contains proofs, simulations, results from additional data analyses, and *.RmD‐files for reproducibility of all results.
