# Supplementary material for: Signpost Testing to Navigate the Parameter Space of the Gaussian Graphical Model With High‐Dimensional Data
Source: Biom J. 2026 Feb 12;68(1):e70115. doi: 10.1002/bimj.70115 (PMC12895234; doi:10.1002/bimj.70115)
Supplement: Supplementary file 1 — Supporting File: bimj70115‐sup‐0001‐Datacode.zip. [file BIMJ-68-e70115-s001.zip › code and data/plot/mispowerplot_SFinv_p50_ManyHandle.pdf]

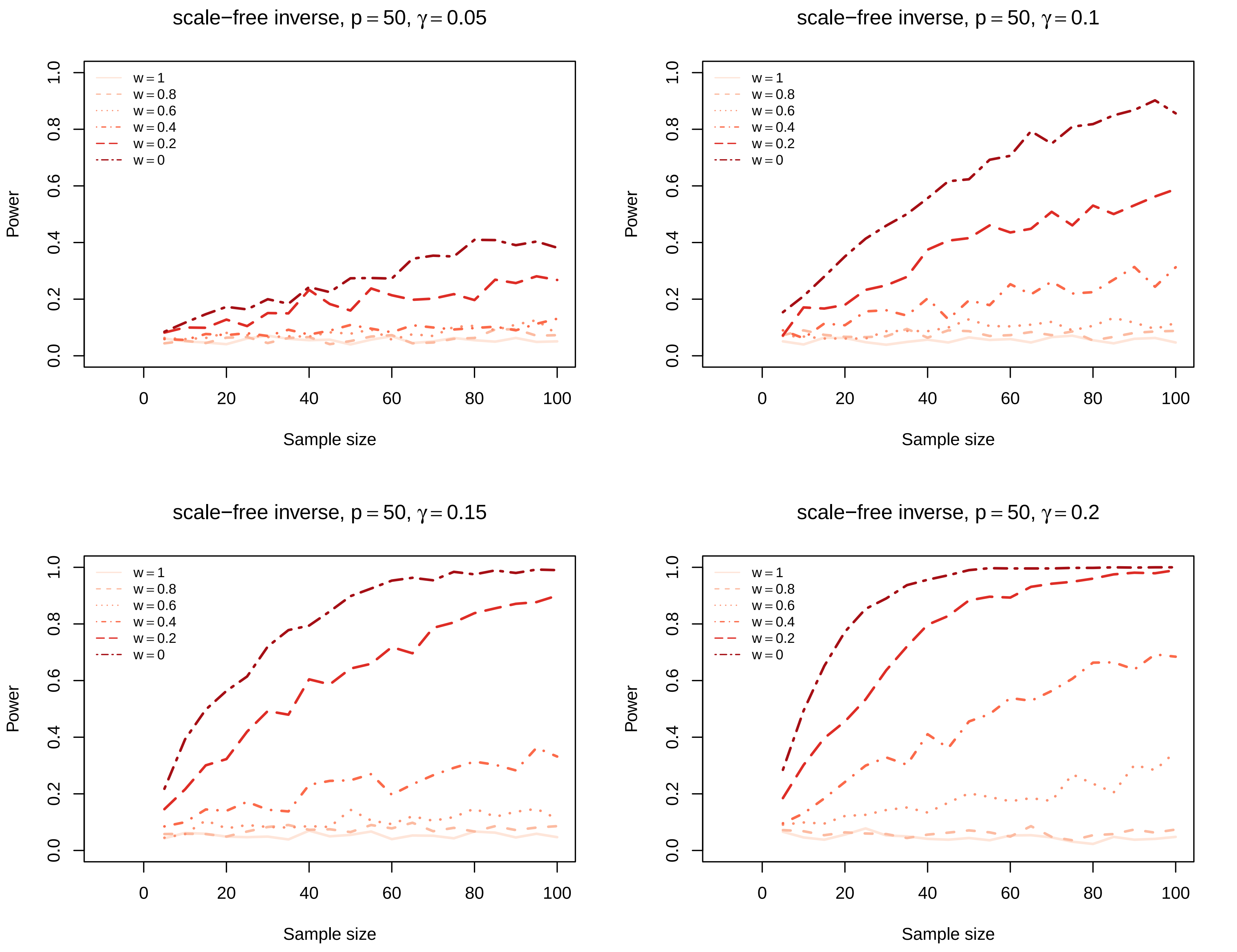
scale−free inverse, p = 50, γ = 0.05
scale−free inverse, p = 50, γ = 0.1
scale−free inverse, p = 50, γ = 0.15
scale−free inverse, p = 50, γ = 0.2
w = 1
w = 0.8
w = 0.6
w = 0.4
w = 0.2
w = 0
Power
Sample size
0.0
0.2
0.4
0.6
0.8
1.0
0
20
40
60
80
100
