# Supplementary material for: Signpost Testing to Navigate the Parameter Space of the Gaussian Graphical Model With High‐Dimensional Data
Source: Biom J. 2026 Feb 12;68(1):e70115. doi: 10.1002/bimj.70115 (PMC12895234; doi:10.1002/bimj.70115)
Supplement: Supplementary file 1 — Supporting File: bimj70115‐sup‐0001‐Datacode.zip. [file BIMJ-68-e70115-s001.zip › code and data/Script_Data_application.html]

Supplementary material to Signpost testing to navigate the parameter space of the Gaussian graphical model with high-dimensional data


# Supplementary material to Signpost testing to navigate the parameter space of the Gaussian graphical model with high-dimensional data

#### Kai Ruan, Mark A. van de Wiel, Wessel N. van Wieringen

#### 2025-10

## Load packages

```
# library(readr)
library(tidyverse)
```

```
## ── Attaching core tidyverse packages ──────────────────────── tidyverse 2.0.0 ──
## ✔ dplyr     1.1.1     ✔ readr     2.1.4
## ✔ forcats   1.0.0     ✔ stringr   1.5.0
## ✔ ggplot2   3.4.1     ✔ tibble    3.2.1
## ✔ lubridate 1.9.2     ✔ tidyr     1.3.0
## ✔ purrr     1.0.1     
## ── Conflicts ────────────────────────────────────────── tidyverse_conflicts() ──
## ✖ dplyr::filter() masks stats::filter()
## ✖ dplyr::lag()    masks stats::lag()
## ℹ Use the ]8;;http://conflicted.r-lib.org/conflicted package]8;; to force all conflicts to become errors
```

```
# library("GGMselect")
library(mvtnorm)
library(rags2ridges)
library(ggplot2)
# library(huge)
library(MVN)
library(LaplacesDemon)
```

```
## 
## Attaching package: 'LaplacesDemon'
## 
## The following objects are masked from 'package:mvtnorm':
## 
##     dmvt, rmvt
## 
## The following objects are masked from 'package:lubridate':
## 
##     dst, interval
## 
## The following object is masked from 'package:purrr':
## 
##     partial
```

```
library(tidyr)
library(reshape2) # function: melt
```

```
## 
## Attaching package: 'reshape2'
## 
## The following object is masked from 'package:tidyr':
## 
##     smiths
```

```
library(ggpubr)
library(MASS) # use generalized inverse function: ginv()
```

```
## 
## Attaching package: 'MASS'
## 
## The following object is masked from 'package:dplyr':
## 
##     select
```

```
library(CVglasso) # cross validation of Gaussian LASSO
```

```
## Loading required package: doParallel
## Loading required package: foreach
## 
## Attaching package: 'foreach'
## 
## The following objects are masked from 'package:purrr':
## 
##     accumulate, when
## 
## Loading required package: iterators
## Loading required package: parallel
```

```
dir.create("./result")
dir.create("./plot")
```

## Load function

```
# For symmetric p.s.d T0, Ta,
# gamma_lower = min {theta: T0 + theta*( Ta - T0 ) is p.s.d.}
# gamma_upper = max {theta: T0 + theta*( Ta - T0 ) is p.s.d.}
# input: T0,Ta
# output: c(gamma_lower,gamma_upper)

range_theta <- function(T0,Ta){
  gamma_calculate_upper=seq(1,10,by=0.001)
  for( i in 1: length(gamma_calculate_upper)){
    Omega_upper = T0 + gamma_calculate_upper[i]*(Ta-T0)
    eigenvalue = eigen(Omega_upper,symmetric = T)$values
    if (min(eigenvalue)<0){
      gamma_upper=gamma_calculate_upper[i-1]
      break
    }
  }
  gamma_calculate_lower=seq(0,-10,by=-0.001)
  for( i in 1: length(gamma_calculate_lower)){
    Omega_lower = T0 + gamma_calculate_lower[i]*(Ta-T0)
    eigenvalue = eigen(Omega_lower,symmetric = T)$values
    if (min(eigenvalue)<0){
      gamma_lower=gamma_calculate_lower[i-1]
      break
    }
  }
  return(c(gamma_lower,gamma_upper))
}

# check function description in the simulation file
theta_estimating_equation_origin<-function(T1,T2,S,theta){ 
  T_tilde<-(1-theta)*T1 + theta*T2
  out=sum(diag( (solve(T_tilde)-S)%*%(-T1+T2) ))
  return(out)
}

# check function description in the simulation file
theta_estimating_equation<-function(T1,T2,S,theta){ 
  p=ncol(S)
  temp1 <- p - theta* sum((T2-T1)*S)
  temp2_1 <- eigen(T1,symmetric = T)
  temp2_2 <- temp2_1$vectors %*% diag(sqrt(temp2_1$values)) %*% t(temp2_1$vectors)
  temp2_3 <- solve(temp2_2) %*% T2 %*% solve(temp2_2)
  temp3 <- sum(1/(1-theta + theta* eigen(temp2_3,symmetric = T)$value))
  out = temp1 - temp3
  return(out)
}

# compute theta_infty in [0,1]
# function depends on ``theta_estimating_equation'' and ``theta_estimating_equation_origin''
compute_thetainf_01 <- function(S,T0,Ta){
  if(theta_estimating_equation(T1=T0,T2=Ta,S=S,theta=0.0001)*theta_estimating_equation(T1=T0,T2=Ta,S=S,theta=1)<0){
      theta_hat=uniroot(theta_estimating_equation,c(0.0001,1),tol=1e-5,T1=T0,T2=Ta,S=S_hat)$root
    }else if( abs(theta_estimating_equation_origin(T0,Ta,S=S_hat,theta=0))<=
              abs(theta_estimating_equation_origin(T0,Ta,S=S_hat,theta=1))){
      theta_hat=0
    }else if( abs(theta_estimating_equation_origin(T0,Ta,S=S_hat,theta=0))>
              abs(theta_estimating_equation_origin(T0,Ta,S=S_hat,theta=1))){ 
      theta_hat=1
    }
  return(theta_hat)
}

# check function description in the simulation file
compute_pval<-function(T1,T2,theta,theta_null,mu,n,B=1000){ 
  # theta represents the estimate of gamma irrespective of the resampling procedure.
  p=ncol(T1)
  
  theta_res<-numeric(B) #generate a vector of length B containing estimated theta of the resampling procedure
  T_tilde_null= (1-theta_null)*T1 + theta_null*T2 # weighted average of the target precision matrix under H0
  
  # terms shared in the resampling procedure
  temp2_1 <- eigen(T1,symmetric = T)
  temp2_2 <- temp2_1$vectors %*% diag(sqrt(temp2_1$values)) %*% t(temp2_1$vectors)
  temp2_3 <- solve(temp2_2) %*% T2 %*% solve(temp2_2)
  temp2_4_eigenvalue <- eigen(temp2_3,symmetric = T)$value
  mat_dif<-T2-T1
  
  # function for efficient computation
  fun <- function(theta,temp1,temp2_4_eigenvalue){
    left= p - theta* temp1
    right= sum(1/(1-theta + theta* temp2_4_eigenvalue))
    output=left-right
    return(output)
  }
  
  for(b in 1:B){
    data_resample = rmvnormal(n=n, mu=mu, sigma=solve(T_tilde_null)) # generate dataset with parametric bootstrap
    S_hat_resample = t(data_resample) %*% data_resample/n # compute the sample covariance matrix
    
    p=ncol(S_hat_resample)
    temp1 <- sum(mat_dif*S_hat_resample)
    
    #estimate theta
    if(fun(theta=0.0001,temp1=temp1,temp2_4_eigenvalue=temp2_4_eigenvalue)*fun(theta=1,temp1=temp1,temp2_4_eigenvalue=temp2_4_eigenvalue) < 0 ){
      root=uniroot(fun,c(0.0001,1),tol=1e-5,temp1=temp1,temp2_4_eigenvalue=temp2_4_eigenvalue)$root
    }
    else if( abs(theta_estimating_equation_origin(T1,T2,S=S_hat_resample,theta=0))<=
             abs(theta_estimating_equation_origin(T1,T2,S=S_hat_resample,theta=1))){
      root=0
    }
    else if( abs(theta_estimating_equation_origin(T1,T2,S=S_hat_resample,theta=0))>
             abs(theta_estimating_equation_origin(T1,T2,S=S_hat_resample,theta=1))){ 
      root=1
    }
    theta_res[b]=root
  }
  p_val = mean(c(theta_res,theta) >= theta)
  list_out<-list(p_val=p_val,theta_resampling=theta_res)
  return(list_out)
}


# gaussianize:
# input data matrix, row: samples; column: variables
# map entries of each column to a standard normal distribution
# return a matrix that all columns are transformed
gaussianize<-function(X){
  name_variate=colnames(X)
  X=as.matrix(X)
  
  # map entries of each column to a standard normal distribution
  temp_lst=apply(X , MARGIN = 2 , FUN=function(Y){
    # 1. take the empirical distribution for entries of each column 
    # 2. map their empirical quantiles to the quantile of standard normal distribution
    temp=qnorm(ecdf(Y)(Y))
    temp[temp==Inf] <- max(temp[temp!=Inf])+1
    return(temp)
  },simplify = F)
  
  # insert the transformed entries back to each column of a matrix
  mat=matrix(NA,nrow = nrow(X), ncol = ncol(X))
  for (n_col in 1: ncol(X)){
    mat[,n_col]<- temp_lst[[n_col]]
  }
  colnames(mat)=name_variate
  return(mat)
}

# random partition of interval [0,1]
create_alphas<-function(len){
  temp=rdirichlet(n=1, alpha=rep(1,times=len))
  return(as.numeric(temp))
}


## stage 3 
# input: ER- and ER+ dataset
# output: penalized ridge precision matrix estimates using 3 targets: 
#         T0, Ta, and T0+ theta_hat*(Ta-T0), 
#         where theta_hat originates from the setup: (S_neg, T0=I_p, Ta=T_pos)
stage3 <- function(er_neg,er_pos){
  OPT_er_pos <- optPenalty.kCV(Y = as.matrix(er_pos), lambdaMin = 0.001, lambdaMax = 20, step = 100,output = "light",verbose = F)
  Target_er_pos= OPT_er_pos$optPrec
  Target_er_pos= cov2cor(Target_er_pos)
  
  S_hat_neg=t(er_neg) %*% er_neg/nrow(er_neg) # estimate sample covariance matrix
  
  T0=diag(nrow(Target_er_pos))
  Ta=Target_er_pos
  p=ncol(T0)
  
  gamma_calculate_upper=seq(0.1,10,by=0.001)
  for( i in 1: length(gamma_calculate_upper)){
    Omega_upper = T0 + gamma_calculate_upper[i]*(Ta-T0)
    eigenvalue = eigen(Omega_upper,symmetric = T)$values
    if (min(eigenvalue)<0){
      gamma_upper=gamma_calculate_upper[i-1]
      break
    }
  }
  
  gamma_calculate_lower=seq(0,-10,by=-0.001)
  for( i in 1: length(gamma_calculate_lower)){
    Omega_lower = T0 + gamma_calculate_lower[i]*(Ta-T0)
    eigenvalue = eigen(Omega_lower,symmetric = T)$values
    if (min(eigenvalue)<0){
      gamma_lower=gamma_calculate_lower[i-1]
      break
    }
  }
  
  {
    S_hat=S_hat_neg
    if(theta_estimating_equation(T1=T0,T2=Ta,S=S_hat,theta=gamma_lower)*theta_estimating_equation(T1=T0,T2=Ta,S=S_hat,theta=-0.0001)<0){
      theta=uniroot(theta_estimating_equation,c(gamma_lower,-0.0001),tol=1e-5,T1=T0,T2=Ta,S=S_hat)$root
    }else if(theta_estimating_equation(T1=T0,T2=Ta,S=S_hat,theta=0.0001)*theta_estimating_equation(T1=T0,T2=Ta,S=S_hat,theta=gamma_upper)<0){
      theta=uniroot(theta_estimating_equation,c(0.0001,gamma_upper),tol=1e-5,T1=T0,T2=Ta,S=S_hat)$root
    }
    theta
  }
  
  Target_ridgeP=T0+theta*(Ta-T0)
  # T0=optPenalty.kCV(Y = as.matrix(subset2_er_pos), lambdaMin = 0.01, lambdaMax = 10, step = 100,fold=10,
  #                type = "Alt", target=Target_ridgeP)
  
  OPT_er_neg_TargetOmegahat <- optPenalty.kCV(Y = as.matrix(er_neg), lambdaMin = 0.001, lambdaMax = 20, step = 100,
                                              type = "Alt", target=Target_ridgeP,output = "light",verbose = F)
  OPT_er_neg_TargetT0 <- optPenalty.kCV(Y = as.matrix(er_neg), lambdaMin = 0.001, lambdaMax = 20, step = 100,
                                        type = "Alt", target=T0,output = "light",verbose = F)
  OPT_er_neg_TargetTa <- optPenalty.kCV(Y = as.matrix(er_neg), lambdaMin = 0.001, lambdaMax = 20, step = 100,
                                        type = "Alt", target=Ta,output = "light",verbose = F)

  list_targets_ridgeP=list(OPT_er_neg_TargetOmegahat=OPT_er_neg_TargetOmegahat,
                           OPT_er_neg_TargetT0=OPT_er_neg_TargetT0,
                           OPT_er_neg_TargetTa=OPT_er_neg_TargetTa)
  
  target_performance = c(OPT_er_neg_TargetOmegahat $optLambda,
                         OPT_er_neg_TargetT0$optLambda,
                         OPT_er_neg_TargetTa $optLambda)
  names(target_performance)=c("Omega(theta_hat)","T0","Ta")
  list_output<-list(theta=theta,
                    Target_ridgeP=Target_ridgeP,
                    targets_performance=target_performance,
                    list_targets_ridgeP=list_targets_ridgeP)
  return(list_output)
}

## top x edges and probability mass

# remark:
# target=1 Omega(thetahat)
# target=2 T0
# target=3 Ta

probmass_sparsified<-function(topedges,list_target_performance,target){
  p=nrow(list_target_performance[[1]][[1]]$optPrec)
  list_target_performance_sparsify=lapply(list_target_performance,FUN = function(x){
    p = nrow(x[[target]]$optPrec)
    temp = sparsify(x[[target]]$optPrec,threshold = "top",top=topedges)$sparsePrecision
    return(temp )
  })
  
  list_target_performance_sparsify_partial=lapply(list_target_performance,FUN = function(x){
    p = nrow(x[[target]]$optPrec)
    temp = sparsify(x[[target]]$optPrec,threshold = "top",top=topedges)$sparsePrecision
    temp2=cov2cor(temp)
    return(temp2 )
  })
  
  
  list_sparsify_adj_target = lapply(list_target_performance_sparsify,FUN = function(x){
    p = nrow(x)
    adj_mat = matrix(data = as.numeric(as.logical(x)),nrow=p,ncol=p)
    return(adj_mat )
  })
  
  adj_targetsum = matrix(0, ncol = p, nrow = p)
  for(j in 1:6){
    adj_targetsum = adj_targetsum + list_sparsify_adj_target[[j]]
  }
  adj_targetsum_offdiag = adj_targetsum-diag(6,nrow = p)
  
  # table edge-overlap counts from adjacant matrix
  vector_adjmat_offdiag=numeric(7)
  names(vector_adjmat_offdiag) = as.character(0:6)
  
  vector_adjmat_offdiag[1] = (sum(as.vector(adj_targetsum_offdiag) == 0)-p)/2 # remove elements on the diagonal
  for(j in 2:7){
    vector_adjmat_offdiag[j] = sum(as.vector(adj_targetsum_offdiag) == j-1)/2
  }
  
  list_out=list(edges_overlap_count=vector_adjmat_offdiag,
                mat_adj_overlap=adj_targetsum_offdiag,
                sparsified_mat=list_target_performance_sparsify,
                sparsify_mat_partial=list_target_performance_sparsify_partial)
  return(list_out)
}


## Hellinger distance

Hellinger_distance =  function(p_mass,q_mass){
  if (length(p_mass)!=length(q_mass))
    return("p_mass, q_mass have different length")

  dif = sqrt(sum((sqrt(p_mass) - sqrt(q_mass))^2))/sqrt(2)
  return(dif)
}


#compute sparsified adjacent matrices and its sum over the data sets
probmass_sparsified_3datasets<-function(topedges,list_target_performance,target){
  p=nrow(list_target_performance[[1]][[1]]$optPrec)
  list_target_performance_sparsify=lapply(list_target_performance,FUN = function(x){
    p = nrow(x[[target]]$optPrec)
    temp = sparsify(x[[target]]$optPrec,threshold = "top",top=topedges)$sparsePrecision
    return(temp )
  })
  
  list_target_performance_sparsify_partial=lapply(list_target_performance,FUN = function(x){
    p = nrow(x[[target]]$optPrec)
    temp = sparsify(x[[target]]$optPrec,threshold = "top",top=topedges)$sparsePrecision
    temp2=cov2cor(temp)
    return(temp2 )
  })
  
  # transform sparsified precision matrices into sparsified adjacent matrices
  list_sparsify_adj_target = lapply(list_target_performance_sparsify,FUN = function(x){
    p = nrow(x)
    adj_mat = matrix(data = as.numeric(as.logical(x)),nrow=p,ncol=p)
    return(adj_mat )
  })
  
  adj_targetsum = matrix(0, ncol = p, nrow = p)
  for(j in 1:length(list_target_performance)){
    adj_targetsum = adj_targetsum + list_sparsify_adj_target[[j]]
  }
  adj_targetsum_offdiag = adj_targetsum-diag(length(list_target_performance),nrow = p)
  
  # table edge-overlap counts from adjacant matrix
  vector_adjmat_offdiag=numeric(1+length(list_target_performance))
  names(vector_adjmat_offdiag) = as.character(0:length(list_target_performance))
  
  vector_adjmat_offdiag[1] = (sum(as.vector(adj_targetsum_offdiag) == 0)-p)/2 # remove elements on the diagonal
  for(j in 2:(length(list_target_performance)+1)){
    vector_adjmat_offdiag[j] = sum(as.vector(adj_targetsum_offdiag) == j-1)/2
  }
  
  list_out=list(edges_overlap_count=vector_adjmat_offdiag,
                mat_adj_overlap=adj_targetsum_offdiag,
                sparsified_mat=list_target_performance_sparsify,
                sparsify_mat_partial=list_target_performance_sparsify_partial)
  return(list_out)
}


##compute Hellinger_distance between two groups of  
table_topedges_VS_HellingerDist_3datasets<-function(top,list_1,list_2){
  p=nrow(list_target_performance[[1]][[1]]$optPrec)
  mat_prob_Omegatheta_list_1=probmass_sparsified_3datasets(topedges=top,list_target_performance=list_1,
                                         target=1)$mat_adj_overlap/length(list_1)
  mat_prob_T0_list_1=probmass_sparsified_3datasets(topedges=top,list_target_performance=list_1,
                                 target=2)$mat_adj_overlap/length(list_1)
  mat_prob_Ta_list_1=probmass_sparsified_3datasets(topedges=top,list_target_performance=list_1,
                                 target=3)$mat_adj_overlap/length(list_1)
  mat_prob_Omegatheta_list_2=probmass_sparsified_3datasets(topedges=top,list_target_performance=list_2,
                                                target=1)$mat_adj_overlap/length(list_2)
  mat_prob_T0_list_2=probmass_sparsified_3datasets(topedges=top,list_target_performance=list_2,
                                        target=2)$mat_adj_overlap/length(list_2)
  mat_prob_Ta_list_2=probmass_sparsified_3datasets(topedges=top,list_target_performance=list_2,
                                        target=3)$mat_adj_overlap/length(list_2)
  
  distance_Omegatheta=Hellinger_distance(p_mass=as.vector(mat_prob_Omegatheta_list_1),
                                         q_mass=as.vector(mat_prob_Omegatheta_list_2))/top

  distance_T0=Hellinger_distance(p_mass=as.vector(mat_prob_T0_list_1),
                                 q_mass=as.vector(mat_prob_T0_list_2))/top
  
  distance_Ta=Hellinger_distance(p_mass=as.vector(mat_prob_Ta_list_1),
                                 q_mass=as.vector(mat_prob_Ta_list_2))/top
 
  out= c(distance_Omegatheta=distance_Omegatheta,
         distance_T0=distance_T0,
         distance_Ta=distance_Ta)
  return(out)
}

# Hellinger distance for two subsets of targets
distance_vs_topedges_3datasets=function(top,list_1,list_2,list_target_performance){
  datavec <- 1:6
  comb <- as.data.frame(t(combn(datavec, 3))) # choose 3 from all 6 datasets
  distance_mat=matrix(nrow=nrow(comb),ncol=3)
  colnames(distance_mat)=c("Omegatheta  ","T0" ,  "Ta ")
  for (i in 1:nrow(comb)){
    # in each round, split 6 data sets into two groups
    set1=unlist(comb[i,])
    set2= datavec[!(datavec %in% set1)]
    list_1=list(list_target_performance[[set1[1]]],
                list_target_performance[[set1[2]]],
                list_target_performance[[set1[3]]])
    list_2=list(list_target_performance[[set2[1]]],
                list_target_performance[[set2[2]]],
                list_target_performance[[set2[3]]])
    distance_mat[i,]=table_topedges_VS_HellingerDist_3datasets(top,list_1,list_2)
  } 
  return(distance_mat)
}

#input: output from function
#output: Spearman correlations of off-diagonal entries in sparsified precision matrices
correlation_6datasets<-function(list_target_performance,top){
  overlap_Omegatheta=probmass_sparsified(topedges=top,list_target_performance=list_target_performance,
                                         target=1)$sparsify_mat_partial
  overlap_T0=probmass_sparsified(topedges=top,list_target_performance=list_target_performance,
                                 target=2)$sparsify_mat_partial
  overlap_Ta=probmass_sparsified(topedges=top,list_target_performance=list_target_performance,
                                 target=3)$sparsify_mat_partial
  
  list_targets_partial=list(overlap_Omegatheta=overlap_Omegatheta,
                    overlap_T0=overlap_T0,
                    overlap_Ta=overlap_Ta )
  
  # 4 targets each resulting in a correlation matrix
  cor_datasets=lapply(list_targets_partial,FUN=function(x){
    
    # 6 matrices each explicitly converted to a vector
    list_as_vec=lapply(x,FUN=function(x1){
      temp=x1[upper.tri(x1,diag = F)]
      temp2=temp[which(temp!=0)]
      return(temp2)
    })
    
    cor_mat=matrix(NA,ncol=6,nrow=6)
    colnames(cor_mat)=c("vdx","mainz","nki","upp","unt","transbig")
    row.names(cor_mat)=c("vdx","mainz","nki","upp","unt","transbig")
    for(i in 1:6){
      for (j in 1:6){
        cor_mat[i,j]=cor(list_as_vec[[i]],list_as_vec[[j]],method = "spearman")
        }
      }
    return(cor_mat)
    })
  
  return(cor_datasets)
}

# input: gamma; T0; Ta; range: lower and upper bound of gamma; B: number of bootstraps
# output: #B bootstrapping theta_hat's at combindation (T0,Ta,gamma)
frequency_density_ParallelSim <- function (gamma,n,T1,T2,range,B=1000){
  
  p=nrow(T1)
  Omega= T1*(1-gamma) + T2*gamma  
  mu = numeric(p)
  sample_size=n
  theta_hat=numeric(B)
  
  # efficient computation
  temp2_1 <- eigen(T1,symmetric = T)
  temp2_2 <- temp2_1$vectors %*% diag(sqrt(temp2_1$values)) %*% t(temp2_1$vectors)
  temp2_3 <- solve(temp2_2) %*% T2 %*% solve(temp2_2)
  temp2_4_eigenvalue <- eigen(temp2_3,symmetric = T)$value
  mat_dif<-T2-T1
  ####################################### efficient computation
  
  # function for efficient computation
  fun <- function(theta,temp1,temp2_4_eigenvalue){
    left= p - theta* temp1
    right= sum(1/(1-theta + theta* temp2_4_eigenvalue))
    output=left-right
    return(output)
  }
  
  ####################################### function for efficient computation
  
  for(b in 1:B){
    #generate data matrix
    data_mat = rmvnormal(n=sample_size, mu=mu, sigma=solve(Omega)) # data matrix
    S_hat = t(data_mat) %*% data_mat/sample_size # estimated covariance matrix
    
    
    temp1_theta <- sum(mat_dif*S_hat)
    # compute observed theta
    {
      if(fun(theta=range[1],temp1=temp1_theta,temp2_4_eigenvalue=temp2_4_eigenvalue)*fun(theta=-0.0001,temp1=temp1_theta,temp2_4_eigenvalue=temp2_4_eigenvalue) < 0 ){
        root=uniroot(fun,c(gamma_lower,-0.0001),tol=1e-5,temp1=temp1_theta,temp2_4_eigenvalue=temp2_4_eigenvalue)$root
      }
      else if(fun(theta=0.0001,temp1=temp1_theta,temp2_4_eigenvalue=temp2_4_eigenvalue)*fun(theta=range[2],temp1=temp1_theta,temp2_4_eigenvalue=temp2_4_eigenvalue) < 0 ){
        root=uniroot(fun,c(0.0001,gamma_upper),tol=1e-5,temp1=temp1_theta,temp2_4_eigenvalue=temp2_4_eigenvalue)$root
      }
      theta_hat[b]<-root
    }
  }
  return(list(theta=theta_hat))
}
```

## Empirical validty of the \(\theta\) metric

Boxplot: empirical validity

```
df_all=readRDS(file="result/df_all_validity.rds")
df_all_gap5=df_all[df_all$replacement %in% seq(1,max(as.numeric(df_all$replacement)),by=5),]

pdf(file="plot/empirical_validity.pdf",width=9,height = 8)
ggplot(df_all_gap5, aes(x=fct_inorder(replacement), y=theta)) + 
  geom_boxplot(aes(fill=factor(polygon_end)))+
  labs(y= expression(hat(theta)[infinity]), x = "Replacement of samples")+
  scale_fill_discrete(name = "", labels = c("MAINZ", "NKI", "Weighted average","UNT","UPP","VDX"))

dev.off()
```

```
## png 
##   2
```

## Test and fit

\(\hat{\theta}\) and p-values, Table
1, main text

```
set.seed(200)
### compute optimal theta for 6 datasets with T0=identity Ta= ER+
T0=diag(nrow(Target_er_pos_transbig))
p=ncol(T0)
for ( j in 1: length(list_S_neg)){
  Ta=list_Ta_pos[[j]]
  S_hat=list_S_neg[[j]]
    
  list_theta_alldatasets[[j]] = compute_thetainf_01(S = S_hat, T0 = T0, Ta =Ta)
  p_val_alldatasets[[j]]<-compute_pval(T1=T0,T2=Ta,theta=list_theta_alldatasets[[j]],theta_null=0,mu=numeric(p),
                                         n=samplesize_alldatasets_neg[[j]],B=1000)$p_val
  
}
saveRDS(list_theta_alldatasets,"result/list_theta_alldatasets_table1_Section6.2.rds")

table1_maintext= data.frame(name_dat,unlist(samplesize_alldatasets_neg),
                      unlist(samplesize_alldatasets_pos),round(unlist(list_theta_alldatasets),digits = 3),
                      unlist(p_val_alldatasets))
colnames(table1_maintext)=c("dataset","#ER-","#ER+","theta_inf","p-value")
row.names(table1_maintext)=c()

saveRDS(table1_maintext,"result/table1_maintext.rds")
write.csv(x = table1_maintext,file = "result/table1_Section6.2_maintext.csv")
```

Losses against \(\theta\), Figure 3,
main text

```
set.seed(200)
########################### compute quadratic loss and Frobenius loss
list_Floss_alldatasets<-vector("list", length = length(list_S_neg))
list_l2loss_alldatasets<-vector("list", length = length(list_S_neg))

theta_grid<-seq(0,1,0.001)

for(i in 1:6){
  Floss_temp=lapply(theta_grid,
         FUN = function(x,T0,Ta,S){
           Omega_theta=T0+x*(Ta-T0)
           F_loss= sqrt(sum((solve(Omega_theta)-S)^2))
           },
         T0=diag(p),Ta=list_Ta_pos[[i]],S=list_S_neg[[i]])
  list_Floss_alldatasets[[i]]=unlist(Floss_temp)
  l2loss_temp=lapply(theta_grid,
                    FUN = function(x,T0,Ta,S){
                      Omega_theta=T0+x*(Ta-T0)
                      l2_loss= sqrt(sum((Omega_theta %*% S-diag(p))^2))
                    },
                    T0=diag(p),Ta=list_Ta_pos[[i]],S=list_S_neg[[i]])
  list_l2loss_alldatasets[[i]]=unlist(l2loss_temp)
} 
saveRDS(object = list_Floss_alldatasets, "result/list_Floss_alldatasets_testandfit.rds")
saveRDS(object = list_l2loss_alldatasets, "result/list_l2loss_alldatasets_testandfit.rds")

list_Floss_alldatasets = readRDS(file= "result/list_Floss_alldatasets_testandfit.rds")
list_l2loss_alldatasets = readRDS(file = "result/list_l2loss_alldatasets_testandfit.rds")
list_theta_alldatasets <- readRDS("result/list_theta_alldatasets_table1_Section6.2.rds")
theta_grid<-seq(0,1,0.001)

pdf("plot/losses_6.2.pdf",width=10,height=5)
par(mfrow=c(1,2))
plot(0,type="n",xlim=c(0,1),ylim=c(15,35),xlab=expression(theta),ylab="Frobenius loss")
for (i in 1:6){
  lines(x=theta_grid,y=list_Floss_alldatasets[[i]],col=i,lty=i,lwd=2)
  temp=which.min(abs(list_theta_alldatasets[[i]]-theta_grid))
  points(x=theta_grid[temp],y=list_Floss_alldatasets[[i]][temp],pch=16,col=i,cex=1.5)
}
legend(list(x = 0.10,y = 35),legend = c("VDX","MAINZ","NKI","UPP","UNT","TRANSBIG"),
       col=c(1,2,3,4,5,6),
       bty = "n", # Removes the legend box
       lty=1:6,
       pch=16,
       cex = 0.95)

plot(0,type="n",xlim=c(0,1),ylim=c(10,35),xlab=expression(theta),ylab="Quadratic loss")
for (i in 1:6){
  lines(x=theta_grid,y=list_l2loss_alldatasets[[i]],col=i,lty=i,lwd=2)
  temp=which.min(abs(list_theta_alldatasets[[i]]-theta_grid))
  points(x=theta_grid[temp],y=list_l2loss_alldatasets[[i]][temp],pch=16,col=i,cex=1.5)
}
legend(list(x = 0.35,y = 35),legend = c("VDX","MAINZ","NKI","UPP","UNT","TRANSBIG"),
       col=c(1,2,3,4,5,6),
       bty = "n", # Removes the legend box
       lty=1:6,
       pch=16,
       cex = 0.95)
par(mfrow=c(1,1))
dev.off()
```

```
## png 
##   2
```

## Towards a conditional independence graph

Check targets’ performance: Table 1, supplementary material

```
set.seed(200)
vdx_result = stage3(er_neg = er_negative_vdx,er_pos = er_positive_vdx)
```

```
mainz_result = stage3(er_neg = er_negative_mainz,er_pos = er_positive_mainz)
```

```
nki_result = stage3(er_neg = er_negative_nki,er_pos = er_positive_nki)
```

```
upp_result = stage3(er_neg = er_negative_upp,er_pos = er_positive_upp)
```

```
unt_result = stage3(er_neg = er_negative_unt, er_pos = er_positive_unt)
```

```
transbig_result = stage3(er_neg = er_negative_transbig,er_pos = er_positive_transbig)
```

```
table1_SM=as.data.frame(cbind(round(vdx_result$targets_performance,digits = 4),
                                          round(mainz_result$targets_performance,digits = 4),
                                          round(nki_result$targets_performance,digits = 4),
                                          round(upp_result$targets_performance,digits = 4),
                                          round(unt_result$targets_performance,digits = 4),
                                          round(transbig_result$targets_performance,digits = 4)))
colnames(table1_SM)<-name_dat
saveRDS(table1_SM,"result/table1_SM.rds")
write.csv(x = table1_SM,file = "result/table1_SM.csv")
```

Hellinger distance:

plot Hellinger distance: figure 19, supplementary materials

```
topvec=seq(10,500,20) # take top 500 edges (in absolute sense)
result_distance_3datasets=readRDS(file="result/result_distance_3datasets.RData")

mean_distance=lapply(result_distance_3datasets,colMeans)
min_distance_mat = matrix(nrow=length(result_distance_3datasets),ncol=3)

for(i in 1:length(result_distance_3datasets)){
  min_distance_mat[i,]=mean_distance[[i]]
}

pdf("plot/distance_500.pdf",width=6,height=6)

plot(0,type="n",xlim=c(0,500),ylim=c(0,0.45),xlab="Number of Top Edges",ylab="Hellinger Distance")
lines(x=topvec,y=min_distance_mat[,1],col=1,lty=1,lwd=2)
lines(x=topvec,y=min_distance_mat[,2],col=2,lty=2,lwd=2)
lines(x=topvec,y=min_distance_mat[,3],col=3,lty=3,lwd=2)

legend(#list(x=200,y=0.2),
       "topright",
       legend = c(expression(paste((1-hat(theta)[infinity]),T[0]+hat(theta)[infinity],T[a])),expression(T[0]),expression(T[a])),
       col=c(1,2,3),
       bty = "n", # Removes the legend box
       lty=1:3,
       cex =1,
       lwd=2)
dev.off()
```

```
## png 
##   2
```

Plot spearman correlations for the sparsified partial correlation
matrices, Figure 18, supplementary material

```
# first form 6 vectors each containing partial correlations 
# second compute correlation matrix among these 6 vectors
top= c(20,50,100,200)
for (t in top){
  cor_results<-correlation_6datasets(list_target_performance = list_target_performance,top=t)
  saveRDS(object = cor_results, paste("result/spearman_cor_results_top",t,".rds",sep = ""))
}
```

```
## - Retained elements:  20 
## - Corresponding to 0.28 % of possible edges 
##  
## - Retained elements:  20 
## - Corresponding to 0.28 % of possible edges 
##  
## - Retained elements:  20 
## - Corresponding to 0.28 % of possible edges 
##  
## - Retained elements:  20 
## - Corresponding to 0.28 % of possible edges 
##  
## - Retained elements:  20 
## - Corresponding to 0.28 % of possible edges 
##  
## - Retained elements:  20 
## - Corresponding to 0.28 % of possible edges 
##  
## - Retained elements:  20 
## - Corresponding to 0.28 % of possible edges 
##  
## - Retained elements:  20 
## - Corresponding to 0.28 % of possible edges 
##  
## - Retained elements:  20 
## - Corresponding to 0.28 % of possible edges 
##  
## - Retained elements:  20 
## - Corresponding to 0.28 % of possible edges 
##  
## - Retained elements:  20 
## - Corresponding to 0.28 % of possible edges 
##  
## - Retained elements:  20 
## - Corresponding to 0.28 % of possible edges 
##  
## - Retained elements:  20 
## - Corresponding to 0.28 % of possible edges 
##  
## - Retained elements:  20 
## - Corresponding to 0.28 % of possible edges 
##  
## - Retained elements:  20 
## - Corresponding to 0.28 % of possible edges 
##  
## - Retained elements:  20 
## - Corresponding to 0.28 % of possible edges 
##  
## - Retained elements:  20 
## - Corresponding to 0.28 % of possible edges 
##  
## - Retained elements:  20 
## - Corresponding to 0.28 % of possible edges 
##  
## - Retained elements:  20 
## - Corresponding to 0.28 % of possible edges 
##  
## - Retained elements:  20 
## - Corresponding to 0.28 % of possible edges 
##  
## - Retained elements:  20 
## - Corresponding to 0.28 % of possible edges 
##  
## - Retained elements:  20 
## - Corresponding to 0.28 % of possible edges 
##  
## - Retained elements:  20 
## - Corresponding to 0.28 % of possible edges 
##  
## - Retained elements:  20 
## - Corresponding to 0.28 % of possible edges 
##  
## - Retained elements:  20 
## - Corresponding to 0.28 % of possible edges 
##  
## - Retained elements:  20 
## - Corresponding to 0.28 % of possible edges 
##  
## - Retained elements:  20 
## - Corresponding to 0.28 % of possible edges 
##  
## - Retained elements:  20 
## - Corresponding to 0.28 % of possible edges 
##  
## - Retained elements:  20 
## - Corresponding to 0.28 % of possible edges 
##  
## - Retained elements:  20 
## - Corresponding to 0.28 % of possible edges 
##  
## - Retained elements:  20 
## - Corresponding to 0.28 % of possible edges 
##  
## - Retained elements:  20 
## - Corresponding to 0.28 % of possible edges 
##  
## - Retained elements:  20 
## - Corresponding to 0.28 % of possible edges 
##  
## - Retained elements:  20 
## - Corresponding to 0.28 % of possible edges 
##  
## - Retained elements:  20 
## - Corresponding to 0.28 % of possible edges 
##  
## - Retained elements:  20 
## - Corresponding to 0.28 % of possible edges 
##  
## - Retained elements:  50 
## - Corresponding to 0.7 % of possible edges 
##  
## - Retained elements:  50 
## - Corresponding to 0.7 % of possible edges 
##  
## - Retained elements:  50 
## - Corresponding to 0.7 % of possible edges 
##  
## - Retained elements:  50 
## - Corresponding to 0.7 % of possible edges 
##  
## - Retained elements:  50 
## - Corresponding to 0.7 % of possible edges 
##  
## - Retained elements:  50 
## - Corresponding to 0.7 % of possible edges 
##  
## - Retained elements:  50 
## - Corresponding to 0.7 % of possible edges 
##  
## - Retained elements:  50 
## - Corresponding to 0.7 % of possible edges 
##  
## - Retained elements:  50 
## - Corresponding to 0.7 % of possible edges 
##  
## - Retained elements:  50 
## - Corresponding to 0.7 % of possible edges 
##  
## - Retained elements:  50 
## - Corresponding to 0.7 % of possible edges 
##  
## - Retained elements:  50 
## - Corresponding to 0.7 % of possible edges 
##  
## - Retained elements:  50 
## - Corresponding to 0.7 % of possible edges 
##  
## - Retained elements:  50 
## - Corresponding to 0.7 % of possible edges 
##  
## - Retained elements:  50 
## - Corresponding to 0.7 % of possible edges 
##  
## - Retained elements:  50 
## - Corresponding to 0.7 % of possible edges 
##  
## - Retained elements:  50 
## - Corresponding to 0.7 % of possible edges 
##  
## - Retained elements:  50 
## - Corresponding to 0.7 % of possible edges 
##  
## - Retained elements:  50 
## - Corresponding to 0.7 % of possible edges 
##  
## - Retained elements:  50 
## - Corresponding to 0.7 % of possible edges 
##  
## - Retained elements:  50 
## - Corresponding to 0.7 % of possible edges 
##  
## - Retained elements:  50 
## - Corresponding to 0.7 % of possible edges 
##  
## - Retained elements:  50 
## - Corresponding to 0.7 % of possible edges 
##  
## - Retained elements:  50 
## - Corresponding to 0.7 % of possible edges 
##  
## - Retained elements:  50 
## - Corresponding to 0.7 % of possible edges 
##  
## - Retained elements:  50 
## - Corresponding to 0.7 % of possible edges 
##  
## - Retained elements:  50 
## - Corresponding to 0.7 % of possible edges 
##  
## - Retained elements:  50 
## - Corresponding to 0.7 % of possible edges 
##  
## - Retained elements:  50 
## - Corresponding to 0.7 % of possible edges 
##  
## - Retained elements:  50 
## - Corresponding to 0.7 % of possible edges 
##  
## - Retained elements:  50 
## - Corresponding to 0.7 % of possible edges 
##  
## - Retained elements:  50 
## - Corresponding to 0.7 % of possible edges 
##  
## - Retained elements:  50 
## - Corresponding to 0.7 % of possible edges 
##  
## - Retained elements:  50 
## - Corresponding to 0.7 % of possible edges 
##  
## - Retained elements:  50 
## - Corresponding to 0.7 % of possible edges 
##  
## - Retained elements:  50 
## - Corresponding to 0.7 % of possible edges 
##  
## - Retained elements:  100 
## - Corresponding to 1.4 % of possible edges 
##  
## - Retained elements:  100 
## - Corresponding to 1.4 % of possible edges 
##  
## - Retained elements:  100 
## - Corresponding to 1.4 % of possible edges 
##  
## - Retained elements:  100 
## - Corresponding to 1.4 % of possible edges 
##  
## - Retained elements:  100 
## - Corresponding to 1.4 % of possible edges 
##  
## - Retained elements:  100 
## - Corresponding to 1.4 % of possible edges 
##  
## - Retained elements:  100 
## - Corresponding to 1.4 % of possible edges 
##  
## - Retained elements:  100 
## - Corresponding to 1.4 % of possible edges 
##  
## - Retained elements:  100 
## - Corresponding to 1.4 % of possible edges 
##  
## - Retained elements:  100 
## - Corresponding to 1.4 % of possible edges 
##  
## - Retained elements:  100 
## - Corresponding to 1.4 % of possible edges 
##  
## - Retained elements:  100 
## - Corresponding to 1.4 % of possible edges 
##  
## - Retained elements:  100 
## - Corresponding to 1.4 % of possible edges 
##  
## - Retained elements:  100 
## - Corresponding to 1.4 % of possible edges 
##  
## - Retained elements:  100 
## - Corresponding to 1.4 % of possible edges 
##  
## - Retained elements:  100 
## - Corresponding to 1.4 % of possible edges 
##  
## - Retained elements:  100 
## - Corresponding to 1.4 % of possible edges 
##  
## - Retained elements:  100 
## - Corresponding to 1.4 % of possible edges 
##  
## - Retained elements:  100 
## - Corresponding to 1.4 % of possible edges 
##  
## - Retained elements:  100 
## - Corresponding to 1.4 % of possible edges 
##  
## - Retained elements:  100 
## - Corresponding to 1.4 % of possible edges 
##  
## - Retained elements:  100 
## - Corresponding to 1.4 % of possible edges 
##  
## - Retained elements:  100 
## - Corresponding to 1.4 % of possible edges 
##  
## - Retained elements:  100 
## - Corresponding to 1.4 % of possible edges 
##  
## - Retained elements:  100 
## - Corresponding to 1.4 % of possible edges 
##  
## - Retained elements:  100 
## - Corresponding to 1.4 % of possible edges 
##  
## - Retained elements:  100 
## - Corresponding to 1.4 % of possible edges 
##  
## - Retained elements:  100 
## - Corresponding to 1.4 % of possible edges 
##  
## - Retained elements:  100 
## - Corresponding to 1.4 % of possible edges 
##  
## - Retained elements:  100 
## - Corresponding to 1.4 % of possible edges 
##  
## - Retained elements:  100 
## - Corresponding to 1.4 % of possible edges 
##  
## - Retained elements:  100 
## - Corresponding to 1.4 % of possible edges 
##  
## - Retained elements:  100 
## - Corresponding to 1.4 % of possible edges 
##  
## - Retained elements:  100 
## - Corresponding to 1.4 % of possible edges 
##  
## - Retained elements:  100 
## - Corresponding to 1.4 % of possible edges 
##  
## - Retained elements:  100 
## - Corresponding to 1.4 % of possible edges 
##  
## - Retained elements:  200 
## - Corresponding to 2.8 % of possible edges 
##  
## - Retained elements:  200 
## - Corresponding to 2.8 % of possible edges 
##  
## - Retained elements:  200 
## - Corresponding to 2.8 % of possible edges 
##  
## - Retained elements:  200 
## - Corresponding to 2.8 % of possible edges 
##  
## - Retained elements:  200 
## - Corresponding to 2.8 % of possible edges 
##  
## - Retained elements:  200 
## - Corresponding to 2.8 % of possible edges 
##  
## - Retained elements:  200 
## - Corresponding to 2.8 % of possible edges 
##  
## - Retained elements:  200 
## - Corresponding to 2.8 % of possible edges 
##  
## - Retained elements:  200 
## - Corresponding to 2.8 % of possible edges 
##  
## - Retained elements:  200 
## - Corresponding to 2.8 % of possible edges 
##  
## - Retained elements:  200 
## - Corresponding to 2.8 % of possible edges 
##  
## - Retained elements:  200 
## - Corresponding to 2.8 % of possible edges 
##  
## - Retained elements:  200 
## - Corresponding to 2.8 % of possible edges 
##  
## - Retained elements:  200 
## - Corresponding to 2.8 % of possible edges 
##  
## - Retained elements:  200 
## - Corresponding to 2.8 % of possible edges 
##  
## - Retained elements:  200 
## - Corresponding to 2.8 % of possible edges 
##  
## - Retained elements:  200 
## - Corresponding to 2.8 % of possible edges 
##  
## - Retained elements:  200 
## - Corresponding to 2.8 % of possible edges 
##  
## - Retained elements:  200 
## - Corresponding to 2.8 % of possible edges 
##  
## - Retained elements:  200 
## - Corresponding to 2.8 % of possible edges 
##  
## - Retained elements:  200 
## - Corresponding to 2.8 % of possible edges 
##  
## - Retained elements:  200 
## - Corresponding to 2.8 % of possible edges 
##  
## - Retained elements:  200 
## - Corresponding to 2.8 % of possible edges 
##  
## - Retained elements:  200 
## - Corresponding to 2.8 % of possible edges 
##  
## - Retained elements:  200 
## - Corresponding to 2.8 % of possible edges 
##  
## - Retained elements:  200 
## - Corresponding to 2.8 % of possible edges 
##  
## - Retained elements:  200 
## - Corresponding to 2.8 % of possible edges 
##  
## - Retained elements:  200 
## - Corresponding to 2.8 % of possible edges 
##  
## - Retained elements:  200 
## - Corresponding to 2.8 % of possible edges 
##  
## - Retained elements:  200 
## - Corresponding to 2.8 % of possible edges 
##  
## - Retained elements:  200 
## - Corresponding to 2.8 % of possible edges 
##  
## - Retained elements:  200 
## - Corresponding to 2.8 % of possible edges 
##  
## - Retained elements:  200 
## - Corresponding to 2.8 % of possible edges 
##  
## - Retained elements:  200 
## - Corresponding to 2.8 % of possible edges 
##  
## - Retained elements:  200 
## - Corresponding to 2.8 % of possible edges 
##  
## - Retained elements:  200 
## - Corresponding to 2.8 % of possible edges 
##
```

```
cor_results_top20=readRDS("result/spearman_cor_results_top20.rds")
cor_results_top50=readRDS("result/spearman_cor_results_top50.rds")
cor_results_top100=readRDS("result/spearman_cor_results_top100.rds")
cor_results_top200=readRDS("result/spearman_cor_results_top200.rds")


par(mfrow=c(1,1))
{
  melted_cormat <- melt(cor_results_top20[[1]], na.rm = TRUE)
  # Heatmap
  p1=ggplot(data = melted_cormat, aes(Var2, Var1, fill = value))+
    geom_tile(color = "white")+
    ggtitle(expression(paste((1-hat(theta)[infinity]),T[0]+hat(theta)[infinity],T[a])))+
    scale_fill_gradient2(low = "blue", high = "red", mid = "white", 
                         midpoint = 0, limit = c(-1,1), space = "Lab", 
                         name="Correlation") +
    theme_minimal()+ 
    theme(axis.text.x = element_text(angle = 45, vjust = 1, 
                                     size = 12, hjust = 1))+
    coord_fixed()
}

{
  melted_cormat <- melt(cor_results_top20[[2]], na.rm = TRUE)
  # Heatmap
  p2=ggplot(data = melted_cormat, aes(Var2, Var1, fill = value))+
    ggtitle(expression(T[0]))+
    geom_tile(color = "white")+
    scale_fill_gradient2(low = "blue", high = "red", mid = "white", 
                         midpoint = 0, limit = c(-1,1), space = "Lab", 
                         name="Correlation") +
    theme_minimal()+ 
    theme(axis.text.x = element_text(angle = 45, vjust = 1, 
                                     size = 12, hjust = 1))+
    coord_fixed()
}

{
  melted_cormat <- melt(cor_results_top20[[3]], na.rm = TRUE)
  # Heatmap
  p3=ggplot(data = melted_cormat, aes(Var2, Var1, fill = value))+
    ggtitle(expression(T[a]))+
    geom_tile(color = "white")+
    scale_fill_gradient2(low = "blue", high = "red", mid = "white", 
                         midpoint = 0, limit = c(-1,1), space = "Lab", 
                         name="Correlation") +
    theme_minimal()+ 
    theme(axis.text.x = element_text(angle = 45, vjust = 1, 
                                     size = 12, hjust = 1))+
    coord_fixed()
}

pdf("plot/cor_top20_spearman.pdf",width=12,height=4)
ggarrange(p1,p2,p3,nrow = 1, ncol = 3) 
dev.off()
```

```
## png 
##   2
```

```
par(mfrow=c(1,1))
{
  melted_cormat <- melt(cor_results_top50[[1]], na.rm = TRUE)
  # Heatmap
  p1=ggplot(data = melted_cormat, aes(Var2, Var1, fill = value))+
    geom_tile(color = "white")+
    ggtitle(expression(paste((1-hat(theta)[infinity]),T[0]+hat(theta)[infinity],T[a])))+
    scale_fill_gradient2(low = "blue", high = "red", mid = "white", 
                         midpoint = 0, limit = c(-1,1), space = "Lab", 
                         name="Correlation") +
    theme_minimal()+ 
    theme(axis.text.x = element_text(angle = 45, vjust = 1, 
                                     size = 12, hjust = 1))+
    coord_fixed()
}

{
  melted_cormat <- melt(cor_results_top50[[2]], na.rm = TRUE)
  # Heatmap
  p2=ggplot(data = melted_cormat, aes(Var2, Var1, fill = value))+
    ggtitle(expression(T[0]))+
    geom_tile(color = "white")+
    scale_fill_gradient2(low = "blue", high = "red", mid = "white", 
                         midpoint = 0, limit = c(-1,1), space = "Lab", 
                         name="Correlation") +
    theme_minimal()+ 
    theme(axis.text.x = element_text(angle = 45, vjust = 1, 
                                     size = 12, hjust = 1))+
    coord_fixed()
}

{
  melted_cormat <- melt(cor_results_top50[[3]], na.rm = TRUE)
  # Heatmap
  p3=ggplot(data = melted_cormat, aes(Var2, Var1, fill = value))+
    ggtitle(expression(T[a]))+
    geom_tile(color = "white")+
    scale_fill_gradient2(low = "blue", high = "red", mid = "white", 
                         midpoint = 0, limit = c(-1,1), space = "Lab", 
                         name="Correlation") +
    theme_minimal()+ 
    theme(axis.text.x = element_text(angle = 45, vjust = 1, 
                                     size = 12, hjust = 1))+
    coord_fixed()
}

pdf("plot/cor_top50_spearman.pdf",width=12,height=4)
ggarrange(p1,p2,p3,nrow = 1, ncol = 3) 
dev.off()
```

```
## png 
##   2
```

```
par(mfrow=c(1,1))
{
  melted_cormat <- melt(cor_results_top100[[1]], na.rm = TRUE)
  # Heatmap
  p1=ggplot(data = melted_cormat, aes(Var2, Var1, fill = value))+
    geom_tile(color = "white")+
    ggtitle(expression(paste((1-hat(theta)[infinity]),T[0]+hat(theta)[infinity],T[a])))+
    scale_fill_gradient2(low = "blue", high = "red", mid = "white", 
                         midpoint = 0, limit = c(-1,1), space = "Lab", 
                         name="Correlation") +
    theme_minimal()+ 
    theme(axis.text.x = element_text(angle = 45, vjust = 1, 
                                     size = 12, hjust = 1))+
    coord_fixed()
}

{
  melted_cormat <- melt(cor_results_top100[[2]], na.rm = TRUE)
  # Heatmap
  p2=ggplot(data = melted_cormat, aes(Var2, Var1, fill = value))+
    ggtitle(expression(T[0]))+
    geom_tile(color = "white")+
    scale_fill_gradient2(low = "blue", high = "red", mid = "white", 
                         midpoint = 0, limit = c(-1,1), space = "Lab", 
                         name="Correlation") +
    theme_minimal()+ 
    theme(axis.text.x = element_text(angle = 45, vjust = 1, 
                                     size = 12, hjust = 1))+
    coord_fixed()
}

{
  melted_cormat <- melt(cor_results_top100[[3]], na.rm = TRUE)
  # Heatmap
  p3=ggplot(data = melted_cormat, aes(Var2, Var1, fill = value))+
    ggtitle(expression(T[a]))+
    geom_tile(color = "white")+
    scale_fill_gradient2(low = "blue", high = "red", mid = "white", 
                         midpoint = 0, limit = c(-1,1), space = "Lab", 
                         name="Correlation") +
    theme_minimal()+ 
    theme(axis.text.x = element_text(angle = 45, vjust = 1, 
                                     size = 12, hjust = 1))+
    coord_fixed()
}

pdf("plot/cor_top100_spearman.pdf",width=12,height=4)
ggarrange(p1,p2,p3,nrow = 1, ncol = 3) 
dev.off()
```

```
## png 
##   2
```

```
par(mfrow=c(1,1))
{
  melted_cormat <- melt(cor_results_top200[[1]], na.rm = TRUE)
  # Heatmap
  p1=ggplot(data = melted_cormat, aes(Var2, Var1, fill = value))+
    geom_tile(color = "white")+
    ggtitle(expression(paste((1-hat(theta)[infinity]),T[0]+hat(theta)[infinity],T[a])))+
    scale_fill_gradient2(low = "blue", high = "red", mid = "white", 
                         midpoint = 0, limit = c(-1,1), space = "Lab", 
                         name="Correlation") +
    theme_minimal()+ 
    theme(axis.text.x = element_text(angle = 45, vjust = 1, 
                                     size = 12, hjust = 1))+
    coord_fixed()
}

{
  melted_cormat <- melt(cor_results_top200[[2]], na.rm = TRUE)
  # Heatmap
  p2=ggplot(data = melted_cormat, aes(Var2, Var1, fill = value))+
    ggtitle(expression(T[0]))+
    geom_tile(color = "white")+
    scale_fill_gradient2(low = "blue", high = "red", mid = "white", 
                         midpoint = 0, limit = c(-1,1), space = "Lab", 
                         name="Correlation") +
    theme_minimal()+ 
    theme(axis.text.x = element_text(angle = 45, vjust = 1, 
                                     size = 12, hjust = 1))+
    coord_fixed()
}

{
  melted_cormat <- melt(cor_results_top200[[3]], na.rm = TRUE)
  # Heatmap
  p3=ggplot(data = melted_cormat, aes(Var2, Var1, fill = value))+
    ggtitle(expression(T[a]))+
    geom_tile(color = "white")+
    scale_fill_gradient2(low = "blue", high = "red", mid = "white", 
                         midpoint = 0, limit = c(-1,1), space = "Lab", 
                         name="Correlation") +
    theme_minimal()+ 
    theme(axis.text.x = element_text(angle = 45, vjust = 1, 
                                     size = 12, hjust = 1))+
    coord_fixed()
}

pdf("plot/cor_top200_spearman.pdf",width=12,height=4)
ggarrange(p1,p2,p3,nrow = 1, ncol = 3) 
dev.off()
```

```
## png 
##   2
```

Reconstruct gene-gene interaction network with top 20 partial
correlations (in absolute sense)

```
set.seed(200)
top=20
Omegatheta_6datasets<-probmass_sparsified(topedges =top,list_target_performance,target=1)
```

```
## - Retained elements:  20 
## - Corresponding to 0.28 % of possible edges 
##  
## - Retained elements:  20 
## - Corresponding to 0.28 % of possible edges 
##  
## - Retained elements:  20 
## - Corresponding to 0.28 % of possible edges 
##  
## - Retained elements:  20 
## - Corresponding to 0.28 % of possible edges 
##  
## - Retained elements:  20 
## - Corresponding to 0.28 % of possible edges 
##  
## - Retained elements:  20 
## - Corresponding to 0.28 % of possible edges 
##  
## - Retained elements:  20 
## - Corresponding to 0.28 % of possible edges 
##  
## - Retained elements:  20 
## - Corresponding to 0.28 % of possible edges 
##  
## - Retained elements:  20 
## - Corresponding to 0.28 % of possible edges 
##  
## - Retained elements:  20 
## - Corresponding to 0.28 % of possible edges 
##  
## - Retained elements:  20 
## - Corresponding to 0.28 % of possible edges 
##  
## - Retained elements:  20 
## - Corresponding to 0.28 % of possible edges 
##
```

```
T0_6datasets<-probmass_sparsified(topedges =top,list_target_performance,target=2)
```

```
## - Retained elements:  20 
## - Corresponding to 0.28 % of possible edges 
##  
## - Retained elements:  20 
## - Corresponding to 0.28 % of possible edges 
##  
## - Retained elements:  20 
## - Corresponding to 0.28 % of possible edges 
##  
## - Retained elements:  20 
## - Corresponding to 0.28 % of possible edges 
##  
## - Retained elements:  20 
## - Corresponding to 0.28 % of possible edges 
##  
## - Retained elements:  20 
## - Corresponding to 0.28 % of possible edges 
##  
## - Retained elements:  20 
## - Corresponding to 0.28 % of possible edges 
##  
## - Retained elements:  20 
## - Corresponding to 0.28 % of possible edges 
##  
## - Retained elements:  20 
## - Corresponding to 0.28 % of possible edges 
##  
## - Retained elements:  20 
## - Corresponding to 0.28 % of possible edges 
##  
## - Retained elements:  20 
## - Corresponding to 0.28 % of possible edges 
##  
## - Retained elements:  20 
## - Corresponding to 0.28 % of possible edges 
##
```

```
Ta_6datasets<-probmass_sparsified(topedges =top,list_target_performance,target=3)
```

```
## - Retained elements:  20 
## - Corresponding to 0.28 % of possible edges 
##  
## - Retained elements:  20 
## - Corresponding to 0.28 % of possible edges 
##  
## - Retained elements:  20 
## - Corresponding to 0.28 % of possible edges 
##  
## - Retained elements:  20 
## - Corresponding to 0.28 % of possible edges 
##  
## - Retained elements:  20 
## - Corresponding to 0.28 % of possible edges 
##  
## - Retained elements:  20 
## - Corresponding to 0.28 % of possible edges 
##  
## - Retained elements:  20 
## - Corresponding to 0.28 % of possible edges 
##  
## - Retained elements:  20 
## - Corresponding to 0.28 % of possible edges 
##  
## - Retained elements:  20 
## - Corresponding to 0.28 % of possible edges 
##  
## - Retained elements:  20 
## - Corresponding to 0.28 % of possible edges 
##  
## - Retained elements:  20 
## - Corresponding to 0.28 % of possible edges 
##  
## - Retained elements:  20 
## - Corresponding to 0.28 % of possible edges 
##
```

```
saveRDS(Omegatheta_6datasets,"result/reconst_Omegatheta_6datasets.rds")
saveRDS(T0_6datasets,"result/reconst_T0_6datasets.rds")
saveRDS(Ta_6datasets,"result/reconst_Ta_6datasets.rds")
```

Figure 20, supplementary material

```
library(rags2ridges)
Omegatheta_6datasets=readRDS("result/reconst_Omegatheta_6datasets.rds")
T0_6datasets=readRDS("result/reconst_T0_6datasets.rds")
Ta_6datasets=readRDS("result/reconst_Ta_6datasets.rds")

pdf("plot/cond_ind_graphsOmega.pdf",width = 5,height = 5)
set.seed(200)
Ugraph(Omegatheta_6datasets$mat_adj_overlap/6, type = "weighted", cut = 0.05, Vsize = 10, Vcex = 0.5,
       lay = "layout_with_fr",prune = T)
```

```
## Warning in type.convert.default(X[[i]], ...): 'as.is' should be specified by
## the caller; using TRUE

## Warning in type.convert.default(X[[i]], ...): 'as.is' should be specified by
## the caller; using TRUE
```

```
##             [,1]       [,2]
##  [1,] -3.5337577  6.9279280
##  [2,]  2.4155684 -6.1229713
##  [3,] -1.7830388 -0.9124285
##  [4,] -4.5719012  1.5981362
##  [5,]  3.2978908 -7.8725012
##  [6,]  4.9649017  3.9569143
##  [7,] -3.7217251  3.2847252
##  [8,] -4.8952409  3.8855868
##  [9,] -8.9157521 -0.7695176
## [10,]  1.7744764  1.6934359
## [11,]  7.2587996 -0.4050316
## [12,] -4.3295099  5.0633720
## [13,] -3.3818404 -0.7604829
## [14,]  2.8473070 -8.5682970
## [15,] -2.8934706  4.8594230
## [16,] -5.9390501  0.3851300
## [17,] -4.8332367 -0.2903986
## [18,]  1.2469426 -7.8476411
## [19,]  3.1250866  6.1396320
## [20,] -1.3811810 -4.2262826
## [21,] -9.4753715 -1.4423280
## [22,] -0.0153081  3.4417349
## [23,]  0.8493886 -0.7423192
## [24,] -7.5412121 -5.4716675
## [25,]  4.6535762 -4.3015025
## [26,]  5.3681568 -1.4801140
## [27,] -4.4400609 -7.4140885
## [28,] -0.6993775 -5.9066700
## [29,]  3.2630314 -5.7816956
## [30,]  5.7770457 -4.6820568
## [31,]  4.4667454 -2.1541652
## [32,]  3.8539729 -3.1505661
## [33,] -1.7021859 -6.9899576
## [34,] -6.6581813  1.2340415
## [35,] -0.1162928 -0.2649538
## [36,] -2.1118783 -5.8055937
## [37,] -1.1042069 -7.8635729
## [38,]  1.9409226 -1.1833943
## [39,]  1.1018851  0.5806037
## [40,] -2.0009070 -9.3560113
## [41,] -6.7572595 -6.1895224
## [42,]  6.9277301  1.7766033
## [43,]  4.0897209  1.1316479
## [44,] -1.7108759  4.6139723
## [45,] -4.8806501 -3.4613331
## [46,]  5.4017651 -3.1706142
## [47,]  0.3345148 -9.2795960
## [48,] -8.4196921  1.4796993
## [49,]  6.4225309  0.2578359
## [50,] -2.3835902  3.1518098
## [51,] -5.4700124 -6.3280412
## [52,] -5.5255639 -7.5540580
## [53,]  1.6352152  5.7713106
## [54,] -2.3814251 -3.3847720
## [55,]  3.4896972  2.8010908
## [56,]  0.4041497 -5.9584940
## [57,] -3.7181413 -8.3469413
## [58,] -4.3884769  6.2537898
## [59,] -3.6048974 -4.5866491
## [60,]  0.9065197  2.7458620
## [61,]  3.1408585  1.8313795
## [62,]  3.4529660  3.7862943
## [63,] -6.4371635 -6.9474147
## [64,] -8.2041591  0.4022381
## [65,] -7.2484211 -3.8570796
## [66,] -4.0261120 -5.5836003
## [67,] -7.1844609  3.5271664
## [68,]  3.8394486  4.6630706
## [69,] -4.8334879 -2.4408395
## [70,] -5.6448611  4.8785242
## [71,] -8.4447896 -2.8706330
## [72,] -2.8868665 -8.9826137
## [73,] -6.2130758 -2.7738507
## [74,] -1.1766567  3.6194865
## [75,]  1.0046128 -8.6878606
## [76,]  6.5983553 -3.1506017
## [77,]  5.5890522 -6.4794915
## [78,] -0.4690633  6.2174720
## [79,] -0.9492317  6.9040236
## [80,]  2.4488241  0.8281308
## [81,]  5.6460342  2.8331711
## [82,]  1.0317128  6.4960967
## [83,]  2.7007555  5.3387889
## [84,] -3.4865031  5.6914734
## [85,] -2.5496527  0.2939744
## [86,] -2.6118500 -4.5256582
## [87,]  4.9195778 -7.0660419
## [88,] -7.4343239 -2.4652216
## [89,] -7.9492310 -0.9515845
## [90,] -6.1704283  3.9543860
## [91,]  4.1145229 -5.2122928
## [92,]  1.9191220 -2.7386225
## [93,]  6.0540930 -0.7724220
## [94,] -3.5755239  1.0838164
## [95,] -6.5568046 -5.0840963
## [96,]  0.1093969 -1.7646756
## [97,]  6.0754121  1.5585656
## [98,]  1.7109259 -3.7453553
```

```
dev.off()
```

```
## png 
##   2
```

```
pdf("plot/cond_ind_graphsT0.pdf",width = 5,height = 5)
set.seed(200)
Ugraph(T0_6datasets$mat_adj_overlap/6, type = "weighted", cut = 0.05, Vsize = 10, Vcex = 0.5,
       lay = "layout_with_fr",prune = T)
```

```
## Warning in type.convert.default(X[[i]], ...): 'as.is' should be specified by
## the caller; using TRUE

## Warning in type.convert.default(X[[i]], ...): 'as.is' should be specified by
## the caller; using TRUE
```

```
##               [,1]       [,2]
##   [1,] -6.60605043  3.3193068
##   [2,]  3.82826613 -7.1729793
##   [3,] -8.49081257 -2.1785693
##   [4,] -3.79964519  4.7312062
##   [5,] -1.00952785  0.1969803
##   [6,] -0.32898181  2.6415692
##   [7,]  1.35927158  7.6225981
##   [8,] -7.13295145 -6.0276950
##   [9,] -7.77007335 -5.3323513
##  [10,] -1.33626173 -0.8589355
##  [11,] -4.65339126 -4.6840538
##  [12,] -3.58807032 -8.1903143
##  [13,]  6.84306875  1.6862219
##  [14,] -8.35506245  0.9166106
##  [15,] -5.95837636  0.4697685
##  [16,] -1.85140655  5.2608800
##  [17,] -3.83056515  7.2783624
##  [18,] -3.72998941  6.2073280
##  [19,] -1.00446773 -8.9681760
##  [20,] -2.85448373 -8.5706020
##  [21,] -9.11887765 -0.0333176
##  [22,]  4.30124606 -0.1518250
##  [23,] -8.51440518  3.8435743
##  [24,]  1.54723222 -8.1783886
##  [25,] -3.31848456  2.9919813
##  [26,] -1.47590052  7.1698419
##  [27,]  5.84700874  3.8744956
##  [28,] -4.99918954 -7.6073145
##  [29,]  3.61987800 -2.5618787
##  [30,]  5.10062984 -6.1133414
##  [31,]  3.26332642 -6.5214805
##  [32,]  3.78985132  4.0872434
##  [33,]  4.69605471  4.6217213
##  [34,]  1.02598511  5.0451381
##  [35,] -8.48581863 -4.6420417
##  [36,] -2.33689105  4.1925610
##  [37,]  5.44189173 -5.3211274
##  [38,]  1.62098877 -1.2315747
##  [39,] -2.57961866 -2.6370457
##  [40,] -4.13624598  2.8631989
##  [41,]  1.74462332  4.4840991
##  [42,]  5.42683942 -3.1534715
##  [43,]  0.81465240 -7.5223324
##  [44,]  4.22247911  0.7820603
##  [45,] -8.19503488 -1.3902714
##  [46,]  6.66263955  2.8501572
##  [47,] -3.34110422 -0.3848476
##  [48,] -5.05196906  3.9652286
##  [49,] -5.66760090  2.8433107
##  [50,] -6.63705090  4.0050223
##  [51,] -0.83269846 -5.7226732
##  [52,]  7.31133208 -0.6006208
##  [53,]  4.38729118 -3.0872573
##  [54,] -5.41347763 -5.4265799
##  [55,] -3.99784928 -1.8656655
##  [56,] -2.75017407 -4.9598951
##  [57,] -4.77748520  0.9164446
##  [58,]  6.62716386  0.5561287
##  [59,]  5.57780457 -4.3345208
##  [60,] -1.15378715  8.1816362
##  [61,] -3.42056956 -4.1519937
##  [62,] -0.43919922  7.3843179
##  [63,]  5.92871954 -1.9195413
##  [64,] -7.54011388  4.5972747
##  [65,] -7.18071613  1.7977632
##  [66,]  0.02893766 -4.1237326
##  [67,]  0.11702146 -5.2090591
##  [68,]  6.35854396 -0.6646932
##  [69,] -1.85903004  1.8846426
##  [70,] -0.59857871 -8.1268255
##  [71,]  0.80419893  6.8329119
##  [72,] -6.37468238  4.6108111
##  [73,] -5.24729936  5.5414562
##  [74,] -4.42600934 -0.7409727
##  [75,]  2.79646388 -4.7786154
##  [76,] -9.27214555 -1.2300095
##  [77,] -3.70018900 -5.4170935
##  [78,] -4.19303128  1.7903398
##  [79,]  2.45572861 -8.1621491
##  [80,]  0.92566393  2.1862673
##  [81,]  2.15686035  2.2342026
##  [82,] -7.23064336 -2.5407271
##  [83,] -1.64727802 -6.5683470
##  [84,] -9.02676905  2.8977290
##  [85,] -7.26385488 -3.5819013
##  [86,]  2.32441810  7.6567006
##  [87,] -5.80392723 -7.3493789
##  [88,]  3.30909317  2.7704378
##  [89,]  0.20520885  3.9644558
##  [90,] -5.04024915 -1.8758647
##  [91,] -6.04351794 -1.6694725
##  [92,]  2.11099818 -4.0707565
##  [93,]  1.14425669 -3.7486768
##  [94,]  7.37703966 -3.0683157
##  [95,] -2.32905701  0.1006797
##  [96,]  5.59299764  3.0378790
##  [97,]  3.51138974  5.3097180
##  [98,] -1.26058996 -4.0195439
##  [99,] -0.20081719 -7.0340010
## [100,]  2.35840744 -0.7827533
## [101,]  1.98225716  5.9091655
## [102,]  6.75954627 -2.4408513
## [103,]  3.87314382  1.7561785
## [104,]  4.11378681  6.1651583
```

```
dev.off()
```

```
## png 
##   2
```

```
pdf("plot/cond_ind_graphsTa.pdf",width = 5,height = 5)
set.seed(200)
Ugraph(Ta_6datasets$mat_adj_overlap/6, type = "weighted", cut = 0.05, Vsize = 10, Vcex = 0.5,
       lay = "layout_with_fr",prune = T)
```

```
## Warning in type.convert.default(X[[i]], ...): 'as.is' should be specified by
## the caller; using TRUE

## Warning in type.convert.default(X[[i]], ...): 'as.is' should be specified by
## the caller; using TRUE
```

```
##              [,1]         [,2]
##  [1,] -10.1155469 -1.795434337
##  [2,]  -5.9606053 -2.938840305
##  [3,]  -0.4664599  5.705763482
##  [4,]  -3.9763290  7.771146208
##  [5,]   5.7850649  0.283825099
##  [6,]  -9.2775503 -4.204986877
##  [7,]  -4.6332831 -4.509997544
##  [8,]  -4.6774997 -2.595951741
##  [9,]  -4.8408860  1.144022238
## [10,]  -1.2967077  0.800431295
## [11,]   0.9560264  0.071538893
## [12,]  -7.9193640 -5.183827013
## [13,]  -3.6908868 -1.432006968
## [14,]  -0.2564008  6.996382881
## [15,]   6.6177052  0.210624411
## [16,]  -2.0084138 -3.281190833
## [17,]   1.7461215  8.201055789
## [18,]   0.6066316  8.070153395
## [19,]   5.2185019  3.973639895
## [20,]  -0.1453270 -0.783504455
## [21,]  -1.5322868 -0.616423408
## [22,]  -0.3344010 -3.374455571
## [23,]   1.3523195  3.086989980
## [24,]   4.0071205 -2.310950840
## [25,]  -7.3920854  1.430543291
## [26,]  -3.6333916  3.668865875
## [27,]   4.4495696 -1.419267432
## [28,]  -9.3030597 -0.389119362
## [29,]  -9.0274353 -1.873023405
## [30,]  -8.1604741  2.303193083
## [31,]  -4.1988000  1.375641764
## [32,]  -5.8931014  2.239412324
## [33,] -10.4716989  1.764180427
## [34,]  -0.6425521 -7.640445895
## [35,]   2.5050790  4.136840059
## [36,]  -9.6072425  1.328813080
## [37,] -10.6064037  2.757215718
## [38,]  -0.7883718  2.876891336
## [39,]   1.6086390 -0.983112522
## [40,]   5.5962865  1.763037514
## [41,]   3.6161801 -4.563381086
## [42,]   5.1358534 -1.294791398
## [43,]  -6.4996327 -5.932666439
## [44,]   3.0132526  2.660852313
## [45,]  -2.5562950 -4.618315722
## [46,]  -9.2637137  4.274544880
## [47,]  -6.4446918  4.042597809
## [48,]  -0.9359304 -4.193576895
## [49,]  -5.7373936  7.952110004
## [50,]   0.6241195  1.118657876
## [51,]  -7.7219033 -3.846982187
## [52,]  -4.4106798 -5.943961025
## [53,]   3.4585866 -3.200675182
## [54,]   2.5770660 -4.921107150
## [55,]  -1.4640808  8.596983637
## [56,]   1.6233237  4.119464891
## [57,]   4.9104508  0.817517867
## [58,]  -2.6659296 -0.987444474
## [59,]  -9.4070650  3.021834083
## [60,]  -0.6137363 -1.813834849
## [61,]   1.7769400  2.168772312
## [62,]   1.7025176  5.900606587
## [63,]   4.2584207 -4.141114991
## [64,]  -6.5390245  7.453663841
## [65,]  -2.9257851  2.132968201
## [66,]   2.7963322  1.076864693
## [67,]   2.0695321 -3.348553570
## [68,]  -6.4750654 -0.601365420
## [69,]   0.9864835  7.079817056
## [70,]  -7.3603265  7.045022484
## [71,]   1.9679816 -6.018233964
## [72,]  -7.3602271  4.941323472
## [73,]  -5.2041517 -1.841711679
## [74,]  -3.8054662  5.377419502
## [75,]   4.6543652  2.924254257
## [76,]  -4.1386222 -6.996932836
## [77,]  -6.2829429  5.275201034
## [78,]  -1.2246485 -6.275222787
## [79,]  -1.5984720 -7.766757748
## [80,]   2.1638772 -0.008901654
## [81,]  -8.5091305 -3.549507401
## [82,]  -2.5247252  8.862397033
## [83,]  -0.2452468  8.121618811
## [84,]  -3.5184618 -2.764986872
## [85,]  -2.4173493  5.554123996
## [86,]   0.9591899 -2.347359838
## [87,]  -1.6014250 -5.145952745
## [88,]  -2.8017507  4.340941410
## [89,]  -2.0003250  2.758915875
## [90,]  -5.2999918 -0.762221958
## [91,]  -8.2586744 -0.204336880
## [92,]   3.4930642  4.380833927
## [93,]  -8.3428153 -2.112385510
## [94,]  -3.2430469  6.880202077
## [95,]   3.6384561 -1.166304651
## [96,]   1.4372920  4.836983449
## [97,]  -7.2060301 -4.762773569
## [98,]  -6.9094472  2.440443641
```

```
dev.off()
```

```
## png 
##   2
```

## Choice of targets

```
set.seed(200)
# obtain alternative target Ta

list_S_neg=readRDS(file="result/list_S_neg.rds")
list_S_pos=readRDS(file="result/list_S_pos.rds")
list_T_er_pos_cor=readRDS(file = "result/list_T_er_pos_cor.rds")

Ta_list_ginv =vector(mode = "list",length=length(list_T_er_pos_cor))
Ta_list_glasso =vector(mode = "list",length=length(list_T_er_pos_cor))

for (i in 1:length(list_T_er_pos_cor)){
  Ta_list_ginv[[i]]   = cov2cor(ginv(list_S_pos[[i]]))
  Ta_list_glasso[[i]] = cov2cor(CVglasso(X=list_data_er_pos[[i]], crit.cv = "loglik")$Omega)
}
```

```
## 
  |                                                                            
  |                                                                      |   0%
  |                                                                            
  |=                                                                     |   2%
  |                                                                            
  |===                                                                   |   4%
  |                                                                            
  |====                                                                  |   6%
  |                                                                            
  |======                                                                |   8%
  |                                                                            
  |=======                                                               |  10%
  |                                                                            
  |========                                                              |  12%
  |                                                                            
  |==========                                                            |  14%
  |                                                                            
  |===========                                                           |  16%
  |                                                                            
  |=============                                                         |  18%
  |                                                                            
  |==============                                                        |  20%
  |                                                                            
  |===============                                                       |  22%
  |                                                                            
  |=================                                                     |  24%
  |                                                                            
  |==================                                                    |  26%
  |                                                                            
  |====================                                                  |  28%
  |                                                                            
  |=====================                                                 |  30%
  |                                                                            
  |======================                                                |  32%
  |                                                                            
  |========================                                              |  34%
  |                                                                            
  |=========================                                             |  36%
  |                                                                            
  |===========================                                           |  38%
  |                                                                            
  |============================                                          |  40%
  |                                                                            
  |=============================                                         |  42%
  |                                                                            
  |===============================                                       |  44%
  |                                                                            
  |================================                                      |  46%
  |                                                                            
  |==================================                                    |  48%
  |                                                                            
  |===================================                                   |  50%
  |                                                                            
  |====================================                                  |  52%
  |                                                                            
  |======================================                                |  54%
  |                                                                            
  |=======================================                               |  56%
  |                                                                            
  |=========================================                             |  58%
  |                                                                            
  |==========================================                            |  60%
  |                                                                            
  |===========================================                           |  62%
  |                                                                            
  |=============================================                         |  64%
  |                                                                            
  |==============================================                        |  66%
  |                                                                            
  |================================================                      |  68%
  |                                                                            
  |=================================================                     |  70%
  |                                                                            
  |==================================================                    |  72%
  |                                                                            
  |====================================================                  |  74%
  |                                                                            
  |=====================================================                 |  76%
  |                                                                            
  |=======================================================               |  78%
  |                                                                            
  |========================================================              |  80%
  |                                                                            
  |=========================================================             |  82%
  |                                                                            
  |===========================================================           |  84%
  |                                                                            
  |============================================================          |  86%
  |                                                                            
  |==============================================================        |  88%
  |                                                                            
  |===============================================================       |  90%
  |                                                                            
  |================================================================      |  92%
  |                                                                            
  |==================================================================    |  94%
  |                                                                            
  |===================================================================   |  96%
  |                                                                            
  |===================================================================== |  98%
  |                                                                            
  |======================================================================| 100%
  |                                                                            
  |                                                                      |   0%
  |                                                                            
  |=                                                                     |   2%
  |                                                                            
  |===                                                                   |   4%
  |                                                                            
  |====                                                                  |   6%
  |                                                                            
  |======                                                                |   8%
  |                                                                            
  |=======                                                               |  10%
  |                                                                            
  |========                                                              |  12%
  |                                                                            
  |==========                                                            |  14%
  |                                                                            
  |===========                                                           |  16%
  |                                                                            
  |=============                                                         |  18%
  |                                                                            
  |==============                                                        |  20%
  |                                                                            
  |===============                                                       |  22%
  |                                                                            
  |=================                                                     |  24%
  |                                                                            
  |==================                                                    |  26%
  |                                                                            
  |====================                                                  |  28%
  |                                                                            
  |=====================                                                 |  30%
  |                                                                            
  |======================                                                |  32%
  |                                                                            
  |========================                                              |  34%
  |                                                                            
  |=========================                                             |  36%
  |                                                                            
  |===========================                                           |  38%
  |                                                                            
  |============================                                          |  40%
  |                                                                            
  |=============================                                         |  42%
  |                                                                            
  |===============================                                       |  44%
  |                                                                            
  |================================                                      |  46%
  |                                                                            
  |==================================                                    |  48%
  |                                                                            
  |===================================                                   |  50%
  |                                                                            
  |====================================                                  |  52%
  |                                                                            
  |======================================                                |  54%
  |                                                                            
  |=======================================                               |  56%
  |                                                                            
  |=========================================                             |  58%
  |                                                                            
  |==========================================                            |  60%
  |                                                                            
  |===========================================                           |  62%
  |                                                                            
  |=============================================                         |  64%
  |                                                                            
  |==============================================                        |  66%
  |                                                                            
  |================================================                      |  68%
  |                                                                            
  |=================================================                     |  70%
  |                                                                            
  |==================================================                    |  72%
  |                                                                            
  |====================================================                  |  74%
  |                                                                            
  |=====================================================                 |  76%
  |                                                                            
  |=======================================================               |  78%
  |                                                                            
  |========================================================              |  80%
  |                                                                            
  |=========================================================             |  82%
  |                                                                            
  |===========================================================           |  84%
  |                                                                            
  |============================================================          |  86%
  |                                                                            
  |==============================================================        |  88%
  |                                                                            
  |===============================================================       |  90%
  |                                                                            
  |================================================================      |  92%
  |                                                                            
  |==================================================================    |  94%
  |                                                                            
  |===================================================================   |  96%
  |                                                                            
  |===================================================================== |  98%
  |                                                                            
  |======================================================================| 100%
  |                                                                            
  |                                                                      |   0%
  |                                                                            
  |=                                                                     |   2%
  |                                                                            
  |===                                                                   |   4%
  |                                                                            
  |====                                                                  |   6%
  |                                                                            
  |======                                                                |   8%
  |                                                                            
  |=======                                                               |  10%
  |                                                                            
  |========                                                              |  12%
  |                                                                            
  |==========                                                            |  14%
  |                                                                            
  |===========                                                           |  16%
  |                                                                            
  |=============                                                         |  18%
  |                                                                            
  |==============                                                        |  20%
  |                                                                            
  |===============                                                       |  22%
  |                                                                            
  |=================                                                     |  24%
  |                                                                            
  |==================                                                    |  26%
  |                                                                            
  |====================                                                  |  28%
  |                                                                            
  |=====================                                                 |  30%
  |                                                                            
  |======================                                                |  32%
  |                                                                            
  |========================                                              |  34%
  |                                                                            
  |=========================                                             |  36%
  |                                                                            
  |===========================                                           |  38%
  |                                                                            
  |============================                                          |  40%
  |                                                                            
  |=============================                                         |  42%
  |                                                                            
  |===============================                                       |  44%
  |                                                                            
  |================================                                      |  46%
  |                                                                            
  |==================================                                    |  48%
  |                                                                            
  |===================================                                   |  50%
  |                                                                            
  |====================================                                  |  52%
  |                                                                            
  |======================================                                |  54%
  |                                                                            
  |=======================================                               |  56%
  |                                                                            
  |=========================================                             |  58%
  |                                                                            
  |==========================================                            |  60%
  |                                                                            
  |===========================================                           |  62%
  |                                                                            
  |=============================================                         |  64%
  |                                                                            
  |==============================================                        |  66%
  |                                                                            
  |================================================                      |  68%
  |                                                                            
  |=================================================                     |  70%
  |                                                                            
  |==================================================                    |  72%
  |                                                                            
  |====================================================                  |  74%
  |                                                                            
  |=====================================================                 |  76%
  |                                                                            
  |=======================================================               |  78%
  |                                                                            
  |========================================================              |  80%
  |                                                                            
  |=========================================================             |  82%
  |                                                                            
  |===========================================================           |  84%
  |                                                                            
  |============================================================          |  86%
  |                                                                            
  |==============================================================        |  88%
  |                                                                            
  |===============================================================       |  90%
  |                                                                            
  |================================================================      |  92%
  |                                                                            
  |==================================================================    |  94%
  |                                                                            
  |===================================================================   |  96%
  |                                                                            
  |===================================================================== |  98%
  |                                                                            
  |======================================================================| 100%
  |                                                                            
  |                                                                      |   0%
  |                                                                            
  |=                                                                     |   2%
  |                                                                            
  |===                                                                   |   4%
  |                                                                            
  |====                                                                  |   6%
  |                                                                            
  |======                                                                |   8%
  |                                                                            
  |=======                                                               |  10%
  |                                                                            
  |========                                                              |  12%
  |                                                                            
  |==========                                                            |  14%
  |                                                                            
  |===========                                                           |  16%
  |                                                                            
  |=============                                                         |  18%
  |                                                                            
  |==============                                                        |  20%
  |                                                                            
  |===============                                                       |  22%
  |                                                                            
  |=================                                                     |  24%
  |                                                                            
  |==================                                                    |  26%
  |                                                                            
  |====================                                                  |  28%
  |                                                                            
  |=====================                                                 |  30%
  |                                                                            
  |======================                                                |  32%
  |                                                                            
  |========================                                              |  34%
  |                                                                            
  |=========================                                             |  36%
  |                                                                            
  |===========================                                           |  38%
  |                                                                            
  |============================                                          |  40%
  |                                                                            
  |=============================                                         |  42%
  |                                                                            
  |===============================                                       |  44%
  |                                                                            
  |================================                                      |  46%
  |                                                                            
  |==================================                                    |  48%
  |                                                                            
  |===================================                                   |  50%
  |                                                                            
  |====================================                                  |  52%
  |                                                                            
  |======================================                                |  54%
  |                                                                            
  |=======================================                               |  56%
  |                                                                            
  |=========================================                             |  58%
  |                                                                            
  |==========================================                            |  60%
  |                                                                            
  |===========================================                           |  62%
  |                                                                            
  |=============================================                         |  64%
  |                                                                            
  |==============================================                        |  66%
  |                                                                            
  |================================================                      |  68%
  |                                                                            
  |=================================================                     |  70%
  |                                                                            
  |==================================================                    |  72%
  |                                                                            
  |====================================================                  |  74%
  |                                                                            
  |=====================================================                 |  76%
  |                                                                            
  |=======================================================               |  78%
  |                                                                            
  |========================================================              |  80%
  |                                                                            
  |=========================================================             |  82%
  |                                                                            
  |===========================================================           |  84%
  |                                                                            
  |============================================================          |  86%
  |                                                                            
  |==============================================================        |  88%
  |                                                                            
  |===============================================================       |  90%
  |                                                                            
  |================================================================      |  92%
  |                                                                            
  |==================================================================    |  94%
  |                                                                            
  |===================================================================   |  96%
  |                                                                            
  |===================================================================== |  98%
  |                                                                            
  |======================================================================| 100%
  |                                                                            
  |                                                                      |   0%
  |                                                                            
  |=                                                                     |   2%
  |                                                                            
  |===                                                                   |   4%
  |                                                                            
  |====                                                                  |   6%
  |                                                                            
  |======                                                                |   8%
  |                                                                            
  |=======                                                               |  10%
  |                                                                            
  |========                                                              |  12%
  |                                                                            
  |==========                                                            |  14%
  |                                                                            
  |===========                                                           |  16%
  |                                                                            
  |=============                                                         |  18%
  |                                                                            
  |==============                                                        |  20%
  |                                                                            
  |===============                                                       |  22%
  |                                                                            
  |=================                                                     |  24%
  |                                                                            
  |==================                                                    |  26%
  |                                                                            
  |====================                                                  |  28%
  |                                                                            
  |=====================                                                 |  30%
  |                                                                            
  |======================                                                |  32%
  |                                                                            
  |========================                                              |  34%
  |                                                                            
  |=========================                                             |  36%
  |                                                                            
  |===========================                                           |  38%
  |                                                                            
  |============================                                          |  40%
  |                                                                            
  |=============================                                         |  42%
  |                                                                            
  |===============================                                       |  44%
  |                                                                            
  |================================                                      |  46%
  |                                                                            
  |==================================                                    |  48%
  |                                                                            
  |===================================                                   |  50%
  |                                                                            
  |====================================                                  |  52%
  |                                                                            
  |======================================                                |  54%
  |                                                                            
  |=======================================                               |  56%
  |                                                                            
  |=========================================                             |  58%
  |                                                                            
  |==========================================                            |  60%
  |                                                                            
  |===========================================                           |  62%
  |                                                                            
  |=============================================                         |  64%
  |                                                                            
  |==============================================                        |  66%
  |                                                                            
  |================================================                      |  68%
  |                                                                            
  |=================================================                     |  70%
  |                                                                            
  |==================================================                    |  72%
  |                                                                            
  |====================================================                  |  74%
  |                                                                            
  |=====================================================                 |  76%
  |                                                                            
  |=======================================================               |  78%
  |                                                                            
  |========================================================              |  80%
  |                                                                            
  |=========================================================             |  82%
  |                                                                            
  |===========================================================           |  84%
  |                                                                            
  |============================================================          |  86%
  |                                                                            
  |==============================================================        |  88%
  |                                                                            
  |===============================================================       |  90%
  |                                                                            
  |================================================================      |  92%
  |                                                                            
  |==================================================================    |  94%
  |                                                                            
  |===================================================================   |  96%
  |                                                                            
  |===================================================================== |  98%
  |                                                                            
  |======================================================================| 100%
  |                                                                            
  |                                                                      |   0%
  |                                                                            
  |=                                                                     |   2%
  |                                                                            
  |===                                                                   |   4%
  |                                                                            
  |====                                                                  |   6%
  |                                                                            
  |======                                                                |   8%
  |                                                                            
  |=======                                                               |  10%
  |                                                                            
  |========                                                              |  12%
  |                                                                            
  |==========                                                            |  14%
  |                                                                            
  |===========                                                           |  16%
  |                                                                            
  |=============                                                         |  18%
  |                                                                            
  |==============                                                        |  20%
  |                                                                            
  |===============                                                       |  22%
  |                                                                            
  |=================                                                     |  24%
  |                                                                            
  |==================                                                    |  26%
  |                                                                            
  |====================                                                  |  28%
  |                                                                            
  |=====================                                                 |  30%
  |                                                                            
  |======================                                                |  32%
  |                                                                            
  |========================                                              |  34%
  |                                                                            
  |=========================                                             |  36%
  |                                                                            
  |===========================                                           |  38%
  |                                                                            
  |============================                                          |  40%
  |                                                                            
  |=============================                                         |  42%
  |                                                                            
  |===============================                                       |  44%
  |                                                                            
  |================================                                      |  46%
  |                                                                            
  |==================================                                    |  48%
  |                                                                            
  |===================================                                   |  50%
  |                                                                            
  |====================================                                  |  52%
  |                                                                            
  |======================================                                |  54%
  |                                                                            
  |=======================================                               |  56%
  |                                                                            
  |=========================================                             |  58%
  |                                                                            
  |==========================================                            |  60%
  |                                                                            
  |===========================================                           |  62%
  |                                                                            
  |=============================================                         |  64%
  |                                                                            
  |==============================================                        |  66%
  |                                                                            
  |================================================                      |  68%
  |                                                                            
  |=================================================                     |  70%
  |                                                                            
  |==================================================                    |  72%
  |                                                                            
  |====================================================                  |  74%
  |                                                                            
  |=====================================================                 |  76%
  |                                                                            
  |=======================================================               |  78%
  |                                                                            
  |========================================================              |  80%
  |                                                                            
  |=========================================================             |  82%
  |                                                                            
  |===========================================================           |  84%
  |                                                                            
  |============================================================          |  86%
  |                                                                            
  |==============================================================        |  88%
  |                                                                            
  |===============================================================       |  90%
  |                                                                            
  |================================================================      |  92%
  |                                                                            
  |==================================================================    |  94%
  |                                                                            
  |===================================================================   |  96%
  |                                                                            
  |===================================================================== |  98%
  |                                                                            
  |======================================================================| 100%
```

```
#################### obtain test statistic theta for ER- data, where T0=diag(p), Ta=preceding obtained

p_val_ginv = vector("list", length = length(list_T_er_pos_cor))
p_val_glasso = vector("list", length = length(list_T_er_pos_cor))
theta_ginv =numeric(length(list_T_er_pos_cor))
theta_glasso =numeric(length(list_T_er_pos_cor))
samplesize_neg = list(neg_vdx=nrow(er_negative_vdx),
                     neg_mainz=nrow(er_negative_mainz),
                     neg_nki=nrow(er_negative_nki),
                     neg_upp=nrow(er_negative_upp),
                     neg_unt=nrow(er_negative_unt),
                     neg_transbig = nrow(er_negative_transbig))

### compute optimal theta for 6 Ta= er+_ginv[i] i=1,...,6
p=ncol(er_negative_vdx)
T0 = diag(p)

for ( j in 1: length(list_T_er_pos_cor)){
  Ta=Ta_list_ginv[[j]]
  
  gamma_calculate_upper=seq(0.1,10,by=0.001)
  for( i in 1: length(gamma_calculate_upper)){
    Omega_upper = T0 + gamma_calculate_upper[i]*(Ta-T0)
    eigenvalue = eigen(Omega_upper,symmetric = T)$values
    if (min(eigenvalue)<0){
      gamma_upper=gamma_calculate_upper[i-1]
      break
    }
  }

  gamma_calculate_lower=seq(0,-10,by=-0.001)
  for( i in 1: length(gamma_calculate_lower)){
    Omega_lower = T0 + gamma_calculate_lower[i]*(Ta-T0)
    eigenvalue = eigen(Omega_lower,symmetric = T)$values
    if (min(eigenvalue)<0){
      gamma_lower=gamma_calculate_lower[i-1]
      break
    }
  }

  
    S_hat=covML(list_data_er_neg[[j]],cor = T )
    if(theta_estimating_equation(T1=T0,T2=Ta,S=S_hat,theta=gamma_lower)*theta_estimating_equation(T1=T0,T2=Ta,S=S_hat,theta=-0.0001)<0){
      theta=uniroot(theta_estimating_equation,c(gamma_lower,-0.0001),tol=1e-5,T1=T0,T2=Ta,S=S_hat)$root
    }else if(theta_estimating_equation(T1=T0,T2=Ta,S=S_hat,theta=0.0001)*theta_estimating_equation(T1=T0,T2=Ta,S=S_hat,theta=gamma_upper)<0){
      theta=uniroot(theta_estimating_equation,c(0.0001,gamma_upper),tol=1e-5,T1=T0,T2=Ta,S=S_hat)$root
    }
    theta_ginv[j]=theta

    theta_res =frequency_density_ParallelSim(gamma=0,n=samplesize_neg[[j]],T1=T0,T2=Ta,
                                             range=c(gamma_lower,gamma_upper),B=1000)[[1]]

    p_val_ginv[j] = mean(c(theta_res,theta_ginv[j]) >= theta_ginv[j])

  
}
```

Table 2, supplementary material

```
table2_SM= data.frame(name_dat,unlist(samplesize_alldatasets_neg),
                      unlist(samplesize_alldatasets_pos),round(theta_ginv,digits = 3),
                      unlist(p_val_ginv))
colnames(table2_SM)=c("dataset","#ER-","#ER+","theta_inf","p-value")
row.names(table2_SM)=c()

saveRDS(theta_ginv,"result/theta_ginv.rds")
saveRDS(table2_SM,"result/table2_SM.rds")
write.csv(x = table2_SM,file = "result/table2_SM.csv")
```

```
### compute optimal theta for 6 Ta= er+_glasso[i] i=1,...,6
p=ncol(er_negative_vdx)
T0 = diag(p)

for ( j in 1: length(list_T_er_pos_cor)){
  Ta=Ta_list_glasso[[j]]
  
  gamma_calculate_upper=seq(0.1,10,by=0.001)
  for( i in 1: length(gamma_calculate_upper)){
    Omega_upper = T0 + gamma_calculate_upper[i]*(Ta-T0)
    eigenvalue = eigen(Omega_upper,symmetric = T)$values
    if (min(eigenvalue)<0){
      gamma_upper=gamma_calculate_upper[i-1]
      break
    }
  }

  gamma_calculate_lower=seq(0,-10,by=-0.001)
  for( i in 1: length(gamma_calculate_lower)){
    Omega_lower = T0 + gamma_calculate_lower[i]*(Ta-T0)
    eigenvalue = eigen(Omega_lower,symmetric = T)$values
    if (min(eigenvalue)<0){
      gamma_lower=gamma_calculate_lower[i-1]
      break
    }
  }

  {
    S_hat=covML(list_data_er_neg[[j]],cor = T )
    if(theta_estimating_equation(T1=T0,T2=Ta,S=S_hat,theta=gamma_lower)*theta_estimating_equation(T1=T0,T2=Ta,S=S_hat,theta=-0.0001)<0){
      theta=uniroot(theta_estimating_equation,c(gamma_lower,-0.0001),tol=1e-5,T1=T0,T2=Ta,S=S_hat)$root
    }else if(theta_estimating_equation(T1=T0,T2=Ta,S=S_hat,theta=0.0001)*theta_estimating_equation(T1=T0,T2=Ta,S=S_hat,theta=gamma_upper)<0){
      theta=uniroot(theta_estimating_equation,c(0.0001,gamma_upper),tol=1e-5,T1=T0,T2=Ta,S=S_hat)$root
    }
    theta_glasso[j]=theta

    theta_res =frequency_density_ParallelSim(gamma=0,n=samplesize_neg[[j]],T1=T0,T2=Ta,
                                             range=c(gamma_lower,gamma_upper),B=1000)[[1]]
    # theta_res[i]=theta_resample(T1=T0,T2=Ta,theta=0,mu=numeric(p),n=samplesize_alldatasets_pos[[j]],B=1000)

    p_val_glasso[[j]] = mean(c(theta_res,theta_glasso[[j]]) >= theta_glasso[j])

  }
}
```

Table 3, supplementary material

```
table3_SM= data.frame(name_dat,unlist(samplesize_alldatasets_neg),
                      unlist(samplesize_alldatasets_pos),round(theta_glasso,digits = 3),
                      unlist(p_val_glasso))
colnames(table3_SM)=c("dataset","#ER-","#ER+","theta_inf","p-value")
row.names(table3_SM)=c()

saveRDS(theta_glasso,"result/theta_glasso.rds")
saveRDS(table3_SM,"result/table3_SM.rds")
write.csv(x = table3_SM,file = "result/table3_SM.csv")
```

```
########################### compute quadratic loss and Frobenius loss for ginv
list_Floss_ginv<-vector("list", length = length(list_S_pos))
list_l2loss_ginv<-vector("list", length = length(list_S_pos))

theta_grid<-seq(0,0.999,0.001)

for(i in 1:length(list_S_pos)){
  Floss_temp=lapply(theta_grid,
                    FUN = function(x,T0,Ta,S){
                      Omega_theta=T0+x*(Ta-T0)
                      F_loss= sqrt(sum((solve(Omega_theta)-S)^2))
                    },
                    T0=diag(p),Ta=Ta_list_ginv[[i]],S=list_S_pos[[i]])
  list_Floss_ginv[[i]]=unlist(Floss_temp)
  l2loss_temp=lapply(theta_grid,
                     FUN = function(x,T0,Ta,S){
                       Omega_theta=T0+x*(Ta-T0)
                       l2_loss= sqrt(sum((Omega_theta %*% S-diag(p))^2))
                     },
                     T0=diag(p),Ta=Ta_list_ginv[[i]],S=list_S_pos[[i]])
  list_l2loss_ginv[[i]]=unlist(l2loss_temp)
} 

saveRDS(list_Floss_ginv,"result/TargetChoice_list_Floss_ginv.rds")
saveRDS(list_l2loss_ginv,"result/TargetChoice_list_l2loss_ginv.rds")


########################### compute quadratic loss and Frobenius loss for glasso
list_Floss_glasso<-vector("list", length = length(list_S_pos))
list_l2loss_glasso<-vector("list", length = length(list_S_pos))

theta_grid<-seq(0,0.999,0.001)

for(i in 1:length(list_S_pos)){
  Floss_temp=lapply(theta_grid,
                    FUN = function(x,T0,Ta,S){
                      Omega_theta=T0+x*(Ta-T0)
                      F_loss= sqrt(sum((solve(Omega_theta)-S)^2))
                    },
                    T0=diag(p),Ta=Ta_list_glasso[[i]],S=list_S_pos[[i]])
  list_Floss_glasso[[i]]=unlist(Floss_temp)
  l2loss_temp=lapply(theta_grid,
                     FUN = function(x,T0,Ta,S){
                       Omega_theta=T0+x*(Ta-T0)
                       l2_loss= sqrt(sum((Omega_theta %*% S-diag(p))^2))
                     },
                     T0=diag(p),Ta=Ta_list_glasso[[i]],S=list_S_pos[[i]])
  list_l2loss_glasso[[i]]=unlist(l2loss_temp)
} 
saveRDS(list_Floss_glasso,"result/TargetChoice_list_Floss_glasso.rds")
saveRDS(list_l2loss_glasso,"result/TargetChoice_list_l2loss_glasso.rds")
```

Loss plot: generalized inverse, Figure 21, supplementary material

```
# loss plot
theta_grid<-seq(0,0.999,0.001)
list_Floss_ginv = readRDS("result/TargetChoice_list_Floss_ginv.rds")
list_l2loss_ginv = readRDS("result/TargetChoice_list_l2loss_ginv.rds")
theta_ginv <- readRDS("result/theta_ginv.rds")

pdf("plot/TargetChoice_loss_ginv.pdf",width=10,height = 5)
par(mfrow=c(1,2))
plot(0,type="n",xlim=c(0,1),ylim=c(10,33),xlab=expression(theta),ylab="Frobenius loss")
for (i in 1:6){
  lines(x=theta_grid,y=list_Floss_ginv[[i]],col=i,lty=i,lwd=2)
  temp=which.min(abs(theta_ginv[[i]]-theta_grid))
  points(x=theta_grid[temp],y=list_Floss_ginv[[i]][temp],pch=16,col=i,cex=1.5)
}
legend(list(x = 0.25,y = 34),legend = c("VDX","MAINZ","NKI","UPP","UNT","TRANSBIG"),
       col=c(1,2,3,4,5,6),
       bty = "n", # Removes the legend box
       lty=1:6,
       pch=16,
       cex = 0.95)

plot(0,type="n",xlim=c(0,1),ylim=c(7,25),xlab=expression(theta),ylab="Quadratic loss")
for (i in 1:6){
  lines(x=theta_grid,y=list_l2loss_ginv[[i]],col=i,lty=i,lwd=2)
  temp=which.min(abs(theta_ginv[[i]]-theta_grid))
  points(x=theta_grid[temp],y=list_l2loss_ginv[[i]][temp],pch=16,col=i,cex=1.5)
}
legend(list(x = 0.5,y = 26),legend = c("VDX","MAINZ","NKI","UPP","UNT","TRANSBIG"),
       col=c(1,2,3,4,5,6),
       bty = "n", # Removes the legend box
       lty=1:6,
       pch=16,
       cex = 0.95)
mtext(text=expression(paste("Loss, ", bold(T)[a], ": Generalized inverse")), 
      side = 3, line = -1.75, outer = TRUE)
dev.off()
```

```
## png 
##   2
```

```
par(mfrow=c(1,1))
```

Loss plot: Gaussian LASSO, Figure 22, supplementary material

```
#loss plot
theta_grid<-seq(0,0.999,0.001)
list_Floss_glasso = readRDS("result/TargetChoice_list_Floss_glasso.rds")
list_l2loss_glasso = readRDS("result/TargetChoice_list_l2loss_glasso.rds")
theta_glasso <- readRDS("result/theta_glasso.rds")


pdf("plot/TargetChoice_loss_glasso.pdf",width=10,height = 5)
par(mfrow=c(1,2))
plot(0,type="n",xlim=c(0,1),ylim=c(5,28),xlab=expression(theta),ylab="Frobenius loss")
for (i in 1:6){
  lines(x=theta_grid,y=list_Floss_glasso[[i]],col=i,lty=i,lwd=2)
  temp=which.min(abs(theta_glasso[[i]]-theta_grid))
  points(x=theta_grid[temp],y=list_Floss_glasso[[i]][temp],pch=16,col=i,cex=1.5)
}
legend(list(x = 0.65,y = 29),legend = c("VDX","MAINZ","NKI","UPP","UNT","TRANSBIG"),
       col=c(1,2,3,4,5,6),
       bty = "n", # Removes the legend box
       lty=1:6,
       pch=16,
       cex = 0.95)

plot(0,type="n",xlim=c(0,1),ylim=c(5,28),xlab=expression(theta),ylab="Quadratic loss")
for (i in 1:6){
  lines(x=theta_grid,y=list_l2loss_glasso[[i]],col=i,lty=i,lwd=2)
  temp=which.min(abs(theta_glasso[[i]]-theta_grid))
  points(x=theta_grid[temp],y=list_l2loss_glasso[[i]][temp],pch=16,col=i,cex=1.5)
}
legend(list(x = 0.65,y = 29),legend = c("VDX","MAINZ","NKI","UPP","UNT","TRANSBIG"),
       col=c(1,2,3,4,5,6),
       bty = "n", # Removes the legend box
       lty=1:6,
       pch=16,
       cex = 0.95)
mtext(text=expression(paste("Loss, ", bold(T)[a],": Gaussian LASSO")), 
      side = 3, line = -1.75, outer = TRUE)

dev.off()
```

```
## png 
##   2
```

```
par(mfrow=c(1,1))
```

## Federated learning

```
set.seed(200)
OPT_er_neg_transbig <- optPenalty.kCV(Y = as.matrix(er_negative_transbig), lambdaMin = 0.0001, lambdaMax = 5, step = 100,output = "light",verbose = F);
```

```
Target_er_neg_transbig<- OPT_er_neg_transbig $optPrec
Target_er_neg_transbig = cov2cor(Target_er_neg_transbig)

Ta_list = list(er_neg_ridgeP = Target_er_neg_transbig )

#################### obtain test statistic theta for ER- data, where T0=diag(p), Ta=Ta_list

p_val_ridgeP =numeric(5)
theta_ridgeP =numeric(5)

samplesize_neg = list(neg_vdx=nrow(er_negative_vdx),
                      neg_mainz=nrow(er_negative_mainz),
                      neg_nki=nrow(er_negative_nki),
                      neg_upp=nrow(er_negative_upp),
                      neg_unt=nrow(er_negative_unt))


### compute optimal theta for T0 = diag(p); Ta= T_er- = ridgeP estimate

### Ta = ridgeP
p=ncol(er_negative_vdx)
T0 = diag(p)

for ( j in 1: length(samplesize_neg )){
  Ta=Ta_list[[1]]
  
  range_temp = range_theta(T0=T0,Ta=Ta)
  gamma_lower = range_temp[1]
  gamma_upper = range_temp[2]
  # 
  S_hat=covML(list_data_er_neg[[j]],cor = T )
  theta_ridgeP[j] = compute_thetainf_01(S=S_hat,T0 = T0,Ta=Ta)
  
  theta_res =frequency_density_ParallelSim(gamma=0,n=samplesize_neg[[j]],T1=T0,T2=Ta,
                                             range=c(gamma_lower,gamma_upper),B=1000)[[1]]
    
  p_val_ridgeP[j] = mean(c(theta_res,theta_ridgeP[j]) >= theta_ridgeP[j])
}


########################### compute quadratic loss and Frobenius loss for ridgeP
list_Floss_ridgeP<-vector("list", length = length(samplesize_neg))
list_l2loss_ridgeP<-vector("list", length = length(samplesize_neg))

theta_grid<-seq(0,0.999,0.001)

for(i in 1:length(samplesize_neg)){
  Floss_temp=lapply(theta_grid,
                    FUN = function(x,T0,Ta,S){
                      Omega_theta=T0+x*(Ta-T0)
                      F_loss= sqrt(sum((solve(Omega_theta)-S)^2))
                    },
                    T0=diag(p),Ta=Ta_list[[1]],S=list_S_pos[[i]])
  list_Floss_ridgeP[[i]]=unlist(Floss_temp)
  l2loss_temp=lapply(theta_grid,
                     FUN = function(x,T0,Ta,S){
                       Omega_theta=T0+x*(Ta-T0)
                       l2_loss= sqrt(sum((Omega_theta %*% S-diag(p))^2))
                     },
                     T0=diag(p),Ta=Ta_list[[1]],S=list_S_pos[[i]])
  list_l2loss_ridgeP[[i]]=unlist(l2loss_temp)
} 

saveRDS(list_Floss_ridgeP,"result/fed_list_Floss_ridgeP.rds")
saveRDS(list_l2loss_ridgeP,"result/fed_list_l2loss_ridgeP.rds")
saveRDS(theta_ridgeP,"result/fed_theta_ridgeP.rds")
saveRDS(p_val_ridgeP,"result/fed_p_val_ridgeP.rds")

name_dat=readRDS(file="result/name_dataset.rds")

table4_SM= data.frame(name_dat[1:5],unlist(samplesize_alldatasets_neg)[1:5],
                      round(theta_ridgeP,digits = 3),
                      p_val_ridgeP)
colnames(table4_SM)=c("dataset","#ER-","theta_inf","p-value")
row.names(table4_SM)=c()

saveRDS(table4_SM,"result/table4_SM.rds")
write.csv(x = table4_SM,file = "result/table4_SM.csv")
```

Loss plot: federated learning, Figure 23, supplementary material

```
theta_grid<-seq(0,0.999,0.001)
list_Floss_ridgeP = readRDS("result/fed_list_Floss_ridgeP.rds")
list_l2loss_ridgeP = readRDS("result/fed_list_l2loss_ridgeP.rds")
theta_ridgeP = readRDS("result/fed_theta_ridgeP.rds")

pdf("plot/fed_learning_lossVStheta.pdf",width=10,height=5)
par(mfrow=c(1,2))
plot(0,type="n",xlim=c(0,1),ylim=c(10,35),xlab=expression(theta),ylab="Frobenius loss")
for (i in 1:5){
  lines(x=theta_grid,y=list_Floss_ridgeP[[i]],col=i,lty=i,lwd=2)
  temp=which.min(abs(theta_ridgeP[[i]]-theta_grid))
  points(x=theta_grid[temp],y=list_Floss_ridgeP[[i]][temp],pch=16,col=i,cex=1.5)
}
legend(list(x = 0.65,y = 33),legend = c("VDX","MAINZ","NKI","UPP","UNT"),
       col=c(1,2,3,4,5),
       bty = "n", # Removes the legend box
       lty=1:5,
       pch=16,
       cex = 0.95)


plot(0,type="n",xlim=c(0,1),ylim=c(10,25),xlab=expression(theta),ylab="Quadratic loss")
for (i in 1:5){
  lines(x=theta_grid,y=list_l2loss_ridgeP[[i]],col=i,lty=i,lwd=2)
  temp=which.min(abs(theta_ridgeP[[i]]-theta_grid))
  points(x=theta_grid[temp],y=list_l2loss_ridgeP[[i]][temp],pch=16,col=i,cex=1.5)
}
legend(list(x = 0.7,y = 25),legend = c("VDX","MAINZ","NKI","UPP","UNT"),
       col=c(1,2,3,4,5),
       bty = "n", # Removes the legend box
       lty=1:5,
       pch=16,
       cex = 0.95)

mtext(text=expression(paste("Loss, ",bold(T)[a]: transbig, " ER-, RidgeP")), 
      side = 3, line = -1.75, outer = TRUE)

dev.off()
```

```
## png 
##   2
```

```
par(mfrow=c(1,1))
```

## Diagnostic plot: Figure 4, main text

```
list_T_er_neg_cor <- readRDS("result/list_T_er_neg_cor.rds")
list_T_er_pos_cor <- readRDS("result/list_T_er_pos_cor.rds")
p=nrow(list_T_er_neg_cor$Target_er_neg_vdx)
y_coor = as.vector(list_T_er_neg_cor$Target_er_neg_vdx-diag(p))


par(mfrow=c(1,2))

y_coord = list_T_er_neg_cor$Target_er_neg_nki[lower.tri(list_T_er_neg_cor$Target_er_neg_nki, diag = FALSE)] # omega_hat - T0
x_coord = list_T_er_pos_cor$Target_er_pos_nki[lower.tri(list_T_er_pos_cor$Target_er_pos_nki, diag = FALSE)] # Ta-T0
#"Sigma_hat^{-1}-T0"

pdf("plot/Diagnostic_plot_left.pdf",width=5,height=5)
plot(x=x_coord,y=y_coord,xlab=expression(T[a]-T[0]),ylab="",
     pch=1,)
mtext(expression(paste(hat(Omega)[nki]-T[0])), side=2, adj=0.5, line=2.5,cex = 1)
dev.off()
```

```
## png 
##   2
```

```
p=nrow(list_T_er_neg_cor$Target_er_neg_vdx)
x_coord = list_T_er_pos_cor$Target_er_pos_vdx[lower.tri(list_T_er_pos_cor$Target_er_pos_vdx, diag = FALSE)] - 
  list_T_er_neg_cor$Target_er_neg_vdx[lower.tri(list_T_er_neg_cor$Target_er_neg_vdx, diag = FALSE)]

y_coord = list_T_er_pos_cor$Target_er_pos_mainz[lower.tri(list_T_er_neg_cor$Target_er_neg_upp, diag=FALSE)] - list_T_er_neg_cor$Target_er_neg_vdx[lower.tri(list_T_er_neg_cor$Target_er_neg_vdx, diag = FALSE)]

pdf("plot/Diagnostic_plot_right.pdf",width=5,height=5)
plot(x=x_coord,y=y_coord,xlab=expression(T[a]-T[0]),ylab="",
     pch=1,)
mtext(expression(paste(hat(Omega)[upp]-T[0])), side=2, adj=0.5, line=2.5,cex = 1)
dev.off()
```

```
## png 
##   2
```

```
par(mfrow=c(1,1))
```
