# Supplementary material for: Signpost Testing to Navigate the Parameter Space of the Gaussian Graphical Model With High‐Dimensional Data
Source: Biom J. 2026 Feb 12;68(1):e70115. doi: 10.1002/bimj.70115 (PMC12895234; doi:10.1002/bimj.70115)
Supplement: Supplementary file 1 — Supporting File: bimj70115‐sup‐0001‐Datacode.zip. [file BIMJ-68-e70115-s001.zip › code and data/Script_plot_allinone.html]

Output


# Output

#### Kai Ruan, Mark A. van de Wiel, Wessel N. van Wieringen

#### 2025-10

## Libraries and functions to use in the simulation

```
## Warning: package 'Matrix' was built under R version 4.2.3
```

```
## Warning: package 'ggridges' was built under R version 4.2.3
```

```
## Warning: package 'huge' was built under R version 4.2.3
```

```
## Warning: package 'MVN' was built under R version 4.2.3
```

```
## Warning: package 'ggpubr' was built under R version 4.2.3
```

```
## Warning: package 'tidyverse' was built under R version 4.2.3
```

```
## Warning: package 'tibble' was built under R version 4.2.3
```

```
## Warning: package 'readr' was built under R version 4.2.3
```

```
## Warning: package 'dplyr' was built under R version 4.2.3
```

```
## Warning: package 'forcats' was built under R version 4.2.3
```

```
## Warning: package 'lubridate' was built under R version 4.2.3
```

```
## ── Attaching core tidyverse packages ──────────────────────── tidyverse 2.0.0 ──
## ✔ dplyr     1.1.1     ✔ readr     2.1.4
## ✔ forcats   1.0.0     ✔ stringr   1.5.0
## ✔ lubridate 1.9.2     ✔ tibble    3.2.1
## ✔ purrr     1.0.1     ✔ tidyr     1.3.0
## ── Conflicts ────────────────────────────────────────── tidyverse_conflicts() ──
## ✖ tidyr::expand() masks Matrix::expand()
## ✖ dplyr::filter() masks stats::filter()
## ✖ dplyr::lag()    masks stats::lag()
## ✖ tidyr::pack()   masks Matrix::pack()
## ✖ tidyr::unpack() masks Matrix::unpack()
## ℹ Use the ]8;;http://conflicted.r-lib.org/conflicted package]8;; to force all conflicts to become errors
```

```
## Warning: package 'reshape2' was built under R version 4.2.3
```

```
## 
## Attaching package: 'reshape2'
## 
## The following object is masked from 'package:tidyr':
## 
##     smiths
```

```
## Warning in dir.create("./result"): '.\result' already exists
```

```
## Warning in dir.create("./plot"): '.\plot' already exists
```

## Figure 1, Section 2.2, main text

```
## Picking joint bandwidth of 0.00422
```

## Figure 2, Section 6.1, main text

## Test and fit: Figure 3, Section 6.2, main text

## Diagnostic plot: Figure 4, main text

## Table 1, Section 6.2, main text

```
##    dataset #ER- #ER+ theta_inf     p-value
## 1      VDX  135  209     0.609 0.000999001
## 2    MAINZ   38  162     0.300 0.000999001
## 3      NKI   51  187     0.487 0.000999001
## 4      UPP   34  213     0.564 0.000999001
## 5      UNT   40   86     0.656 0.000999001
## 6 TRANSBIG   64  134     0.201 0.000999001
```

## Regularization path \(\{\hat{\theta}(\lambda):\lambda>0\}\): Figure 1, supplementary material

## Unbiasedness, panel of \(\theta\_{\infty}\): Figure 2, supplementary material

```
## Picking joint bandwidth of 0.014
```

```
## Picking joint bandwidth of 0.00473
```

```
## Picking joint bandwidth of 0.00422
```

```
## Picking joint bandwidth of 0.0099
```

```
## Picking joint bandwidth of 0.00337
```

```
## Picking joint bandwidth of 0.00298
```

\(\theta\_{\lambda}\) for large \(\lambda\): Figure 2, supplementary
material

```
## Picking joint bandwidth of 0.0116
```

```
## Picking joint bandwidth of 0.00502
```

```
## Picking joint bandwidth of 0.0039
```

## Effect of \(\theta\): Figure 3, supplementary material

## Approximated test statistic, QQ-plot: Figure 4, supplementary material

## Histogram of test statistic vs approximated test statistic, Figure 5, supplementary material

## Matrix topologies: Figure 6, supplementary material

```
## 
## Attaching package: 'MASS'
```

```
## The following object is masked from 'package:dplyr':
## 
##     select
```

## Power plot, Figure 7, supplementary material

## Power plot of \(\theta(\lambda)\), Figure 8, supplementary material

## Power plot, Figure 9, supplementary material

## Power plot, Figure 10, supplementary material

## Power plot, Figure 11, supplementary material

## Power plot, Figure 12, supplementary material

## Misspecification, Figure 13, supplementary material

## Misspecification, Figure 14, supplementary material

## Misspecification, Figure 15, supplementary material

## Comparison LRT vs signpost test, Type I error against sample size: Figure 16, supplementary material

### Comparison LRT vs signpost test, power plot: Figure 17, supplementary material

## Spearman correlations for the sparsified partial correlation matrices: Figure 18, supplementary material

## Hellinger distance: Figure 19, supplementary material

## Figure 20, supplementary material

```
## Warning in type.convert.default(X[[i]], ...): 'as.is' should be specified by
## the caller; using TRUE

## Warning in type.convert.default(X[[i]], ...): 'as.is' should be specified by
## the caller; using TRUE
```

```
##             [,1]       [,2]
##  [1,] -3.5337577  6.9279280
##  [2,]  2.4155684 -6.1229713
##  [3,] -1.7830388 -0.9124285
##  [4,] -4.5719012  1.5981362
##  [5,]  3.2978908 -7.8725012
##  [6,]  4.9649017  3.9569143
##  [7,] -3.7217251  3.2847252
##  [8,] -4.8952409  3.8855868
##  [9,] -8.9157521 -0.7695176
## [10,]  1.7744764  1.6934359
## [11,]  7.2587996 -0.4050316
## [12,] -4.3295099  5.0633720
## [13,] -3.3818404 -0.7604829
## [14,]  2.8473070 -8.5682970
## [15,] -2.8934706  4.8594230
## [16,] -5.9390501  0.3851300
## [17,] -4.8332367 -0.2903986
## [18,]  1.2469426 -7.8476411
## [19,]  3.1250866  6.1396320
## [20,] -1.3811810 -4.2262826
## [21,] -9.4753715 -1.4423280
## [22,] -0.0153081  3.4417349
## [23,]  0.8493886 -0.7423192
## [24,] -7.5412121 -5.4716675
## [25,]  4.6535762 -4.3015025
## [26,]  5.3681568 -1.4801140
## [27,] -4.4400609 -7.4140885
## [28,] -0.6993775 -5.9066700
## [29,]  3.2630314 -5.7816956
## [30,]  5.7770457 -4.6820568
## [31,]  4.4667454 -2.1541652
## [32,]  3.8539729 -3.1505661
## [33,] -1.7021859 -6.9899576
## [34,] -6.6581813  1.2340415
## [35,] -0.1162928 -0.2649538
## [36,] -2.1118783 -5.8055937
## [37,] -1.1042069 -7.8635729
## [38,]  1.9409226 -1.1833943
## [39,]  1.1018851  0.5806037
## [40,] -2.0009070 -9.3560113
## [41,] -6.7572595 -6.1895224
## [42,]  6.9277301  1.7766033
## [43,]  4.0897209  1.1316479
## [44,] -1.7108759  4.6139723
## [45,] -4.8806501 -3.4613331
## [46,]  5.4017651 -3.1706142
## [47,]  0.3345148 -9.2795960
## [48,] -8.4196921  1.4796993
## [49,]  6.4225309  0.2578359
## [50,] -2.3835902  3.1518098
## [51,] -5.4700124 -6.3280412
## [52,] -5.5255639 -7.5540580
## [53,]  1.6352152  5.7713106
## [54,] -2.3814251 -3.3847720
## [55,]  3.4896972  2.8010908
## [56,]  0.4041497 -5.9584940
## [57,] -3.7181413 -8.3469413
## [58,] -4.3884769  6.2537898
## [59,] -3.6048974 -4.5866491
## [60,]  0.9065197  2.7458620
## [61,]  3.1408585  1.8313795
## [62,]  3.4529660  3.7862943
## [63,] -6.4371635 -6.9474147
## [64,] -8.2041591  0.4022381
## [65,] -7.2484211 -3.8570796
## [66,] -4.0261120 -5.5836003
## [67,] -7.1844609  3.5271664
## [68,]  3.8394486  4.6630706
## [69,] -4.8334879 -2.4408395
## [70,] -5.6448611  4.8785242
## [71,] -8.4447896 -2.8706330
## [72,] -2.8868665 -8.9826137
## [73,] -6.2130758 -2.7738507
## [74,] -1.1766567  3.6194865
## [75,]  1.0046128 -8.6878606
## [76,]  6.5983553 -3.1506017
## [77,]  5.5890522 -6.4794915
## [78,] -0.4690633  6.2174720
## [79,] -0.9492317  6.9040236
## [80,]  2.4488241  0.8281308
## [81,]  5.6460342  2.8331711
## [82,]  1.0317128  6.4960967
## [83,]  2.7007555  5.3387889
## [84,] -3.4865031  5.6914734
## [85,] -2.5496527  0.2939744
## [86,] -2.6118500 -4.5256582
## [87,]  4.9195778 -7.0660419
## [88,] -7.4343239 -2.4652216
## [89,] -7.9492310 -0.9515845
## [90,] -6.1704283  3.9543860
## [91,]  4.1145229 -5.2122928
## [92,]  1.9191220 -2.7386225
## [93,]  6.0540930 -0.7724220
## [94,] -3.5755239  1.0838164
## [95,] -6.5568046 -5.0840963
## [96,]  0.1093969 -1.7646756
## [97,]  6.0754121  1.5585656
## [98,]  1.7109259 -3.7453553
```

```
## Warning in type.convert.default(X[[i]], ...): 'as.is' should be specified by
## the caller; using TRUE

## Warning in type.convert.default(X[[i]], ...): 'as.is' should be specified by
## the caller; using TRUE
```

```
##               [,1]       [,2]
##   [1,] -6.60605043  3.3193068
##   [2,]  3.82826613 -7.1729793
##   [3,] -8.49081257 -2.1785693
##   [4,] -3.79964519  4.7312062
##   [5,] -1.00952785  0.1969803
##   [6,] -0.32898181  2.6415692
##   [7,]  1.35927158  7.6225981
##   [8,] -7.13295145 -6.0276950
##   [9,] -7.77007335 -5.3323513
##  [10,] -1.33626173 -0.8589355
##  [11,] -4.65339126 -4.6840538
##  [12,] -3.58807032 -8.1903143
##  [13,]  6.84306875  1.6862219
##  [14,] -8.35506245  0.9166106
##  [15,] -5.95837636  0.4697685
##  [16,] -1.85140655  5.2608800
##  [17,] -3.83056515  7.2783624
##  [18,] -3.72998941  6.2073280
##  [19,] -1.00446773 -8.9681760
##  [20,] -2.85448373 -8.5706020
##  [21,] -9.11887765 -0.0333176
##  [22,]  4.30124606 -0.1518250
##  [23,] -8.51440518  3.8435743
##  [24,]  1.54723222 -8.1783886
##  [25,] -3.31848456  2.9919813
##  [26,] -1.47590052  7.1698419
##  [27,]  5.84700874  3.8744956
##  [28,] -4.99918954 -7.6073145
##  [29,]  3.61987800 -2.5618787
##  [30,]  5.10062984 -6.1133414
##  [31,]  3.26332642 -6.5214805
##  [32,]  3.78985132  4.0872434
##  [33,]  4.69605471  4.6217213
##  [34,]  1.02598511  5.0451381
##  [35,] -8.48581863 -4.6420417
##  [36,] -2.33689105  4.1925610
##  [37,]  5.44189173 -5.3211274
##  [38,]  1.62098877 -1.2315747
##  [39,] -2.57961866 -2.6370457
##  [40,] -4.13624598  2.8631989
##  [41,]  1.74462332  4.4840991
##  [42,]  5.42683942 -3.1534715
##  [43,]  0.81465240 -7.5223324
##  [44,]  4.22247911  0.7820603
##  [45,] -8.19503488 -1.3902714
##  [46,]  6.66263955  2.8501572
##  [47,] -3.34110422 -0.3848476
##  [48,] -5.05196906  3.9652286
##  [49,] -5.66760090  2.8433107
##  [50,] -6.63705090  4.0050223
##  [51,] -0.83269846 -5.7226732
##  [52,]  7.31133208 -0.6006208
##  [53,]  4.38729118 -3.0872573
##  [54,] -5.41347763 -5.4265799
##  [55,] -3.99784928 -1.8656655
##  [56,] -2.75017407 -4.9598951
##  [57,] -4.77748520  0.9164446
##  [58,]  6.62716386  0.5561287
##  [59,]  5.57780457 -4.3345208
##  [60,] -1.15378715  8.1816362
##  [61,] -3.42056956 -4.1519937
##  [62,] -0.43919922  7.3843179
##  [63,]  5.92871954 -1.9195413
##  [64,] -7.54011388  4.5972747
##  [65,] -7.18071613  1.7977632
##  [66,]  0.02893766 -4.1237326
##  [67,]  0.11702146 -5.2090591
##  [68,]  6.35854396 -0.6646932
##  [69,] -1.85903004  1.8846426
##  [70,] -0.59857871 -8.1268255
##  [71,]  0.80419893  6.8329119
##  [72,] -6.37468238  4.6108111
##  [73,] -5.24729936  5.5414562
##  [74,] -4.42600934 -0.7409727
##  [75,]  2.79646388 -4.7786154
##  [76,] -9.27214555 -1.2300095
##  [77,] -3.70018900 -5.4170935
##  [78,] -4.19303128  1.7903398
##  [79,]  2.45572861 -8.1621491
##  [80,]  0.92566393  2.1862673
##  [81,]  2.15686035  2.2342026
##  [82,] -7.23064336 -2.5407271
##  [83,] -1.64727802 -6.5683470
##  [84,] -9.02676905  2.8977290
##  [85,] -7.26385488 -3.5819013
##  [86,]  2.32441810  7.6567006
##  [87,] -5.80392723 -7.3493789
##  [88,]  3.30909317  2.7704378
##  [89,]  0.20520885  3.9644558
##  [90,] -5.04024915 -1.8758647
##  [91,] -6.04351794 -1.6694725
##  [92,]  2.11099818 -4.0707565
##  [93,]  1.14425669 -3.7486768
##  [94,]  7.37703966 -3.0683157
##  [95,] -2.32905701  0.1006797
##  [96,]  5.59299764  3.0378790
##  [97,]  3.51138974  5.3097180
##  [98,] -1.26058996 -4.0195439
##  [99,] -0.20081719 -7.0340010
## [100,]  2.35840744 -0.7827533
## [101,]  1.98225716  5.9091655
## [102,]  6.75954627 -2.4408513
## [103,]  3.87314382  1.7561785
## [104,]  4.11378681  6.1651583
```

```
## Warning in type.convert.default(X[[i]], ...): 'as.is' should be specified by
## the caller; using TRUE

## Warning in type.convert.default(X[[i]], ...): 'as.is' should be specified by
## the caller; using TRUE
```

```
##              [,1]         [,2]
##  [1,] -10.1155469 -1.795434337
##  [2,]  -5.9606053 -2.938840305
##  [3,]  -0.4664599  5.705763482
##  [4,]  -3.9763290  7.771146208
##  [5,]   5.7850649  0.283825099
##  [6,]  -9.2775503 -4.204986877
##  [7,]  -4.6332831 -4.509997544
##  [8,]  -4.6774997 -2.595951741
##  [9,]  -4.8408860  1.144022238
## [10,]  -1.2967077  0.800431295
## [11,]   0.9560264  0.071538893
## [12,]  -7.9193640 -5.183827013
## [13,]  -3.6908868 -1.432006968
## [14,]  -0.2564008  6.996382881
## [15,]   6.6177052  0.210624411
## [16,]  -2.0084138 -3.281190833
## [17,]   1.7461215  8.201055789
## [18,]   0.6066316  8.070153395
## [19,]   5.2185019  3.973639895
## [20,]  -0.1453270 -0.783504455
## [21,]  -1.5322868 -0.616423408
## [22,]  -0.3344010 -3.374455571
## [23,]   1.3523195  3.086989980
## [24,]   4.0071205 -2.310950840
## [25,]  -7.3920854  1.430543291
## [26,]  -3.6333916  3.668865875
## [27,]   4.4495696 -1.419267432
## [28,]  -9.3030597 -0.389119362
## [29,]  -9.0274353 -1.873023405
## [30,]  -8.1604741  2.303193083
## [31,]  -4.1988000  1.375641764
## [32,]  -5.8931014  2.239412324
## [33,] -10.4716989  1.764180427
## [34,]  -0.6425521 -7.640445895
## [35,]   2.5050790  4.136840059
## [36,]  -9.6072425  1.328813080
## [37,] -10.6064037  2.757215718
## [38,]  -0.7883718  2.876891336
## [39,]   1.6086390 -0.983112522
## [40,]   5.5962865  1.763037514
## [41,]   3.6161801 -4.563381086
## [42,]   5.1358534 -1.294791398
## [43,]  -6.4996327 -5.932666439
## [44,]   3.0132526  2.660852313
## [45,]  -2.5562950 -4.618315722
## [46,]  -9.2637137  4.274544880
## [47,]  -6.4446918  4.042597809
## [48,]  -0.9359304 -4.193576895
## [49,]  -5.7373936  7.952110004
## [50,]   0.6241195  1.118657876
## [51,]  -7.7219033 -3.846982187
## [52,]  -4.4106798 -5.943961025
## [53,]   3.4585866 -3.200675182
## [54,]   2.5770660 -4.921107150
## [55,]  -1.4640808  8.596983637
## [56,]   1.6233237  4.119464891
## [57,]   4.9104508  0.817517867
## [58,]  -2.6659296 -0.987444474
## [59,]  -9.4070650  3.021834083
## [60,]  -0.6137363 -1.813834849
## [61,]   1.7769400  2.168772312
## [62,]   1.7025176  5.900606587
## [63,]   4.2584207 -4.141114991
## [64,]  -6.5390245  7.453663841
## [65,]  -2.9257851  2.132968201
## [66,]   2.7963322  1.076864693
## [67,]   2.0695321 -3.348553570
## [68,]  -6.4750654 -0.601365420
## [69,]   0.9864835  7.079817056
## [70,]  -7.3603265  7.045022484
## [71,]   1.9679816 -6.018233964
## [72,]  -7.3602271  4.941323472
## [73,]  -5.2041517 -1.841711679
## [74,]  -3.8054662  5.377419502
## [75,]   4.6543652  2.924254257
## [76,]  -4.1386222 -6.996932836
## [77,]  -6.2829429  5.275201034
## [78,]  -1.2246485 -6.275222787
## [79,]  -1.5984720 -7.766757748
## [80,]   2.1638772 -0.008901654
## [81,]  -8.5091305 -3.549507401
## [82,]  -2.5247252  8.862397033
## [83,]  -0.2452468  8.121618811
## [84,]  -3.5184618 -2.764986872
## [85,]  -2.4173493  5.554123996
## [86,]   0.9591899 -2.347359838
## [87,]  -1.6014250 -5.145952745
## [88,]  -2.8017507  4.340941410
## [89,]  -2.0003250  2.758915875
## [90,]  -5.2999918 -0.762221958
## [91,]  -8.2586744 -0.204336880
## [92,]   3.4930642  4.380833927
## [93,]  -8.3428153 -2.112385510
## [94,]  -3.2430469  6.880202077
## [95,]   3.6384561 -1.166304651
## [96,]   1.4372920  4.836983449
## [97,]  -7.2060301 -4.762773569
## [98,]  -6.9094472  2.440443641
```

## Figure 21, supplementary material

```
## null device 
##           1
```

## Figure 22, supplementary material

## Figure 23, supplementary material

## Table 1, supplementary material

```
##                     VDX  MAINZ    NKI     UPP    UNT TRANSBIG
## Omega(theta_hat) 1.6403 8.1288 6.6548 14.8148 5.4481   6.6548
## T0               1.8128 7.3550 4.9295  8.1288 4.9295   5.4481
## Ta               1.0993 1.8128 1.8128  4.4602 2.4473   0.9947
```

## Table 2, supplementary material

```
##    dataset #ER- #ER+ theta_inf     p-value
## 1      VDX  135  209     0.271 0.000999001
## 2    MAINZ   38  162     0.093 0.000999001
## 3      NKI   51  187     0.256 0.000999001
## 4      UPP   34  213     0.254 0.000999001
## 5      UNT   40   86     0.085 0.000999001
## 6 TRANSBIG   64  134     0.050 0.000999001
```

## Table 3, supplementary material

```
##    dataset #ER- #ER+ theta_inf     p-value
## 1      VDX  135  209     0.899 0.000999001
## 2    MAINZ   38  162     0.731 0.000999001
## 3      NKI   51  187     0.703 0.000999001
## 4      UPP   34  213     0.903 0.000999001
## 5      UNT   40   86     0.912 0.000999001
## 6 TRANSBIG   64  134     0.865 0.000999001
```

## Table 4, supplementary material

```
##   dataset #ER- theta_inf     p-value
## 1     VDX  135     0.953 0.000999001
## 2   MAINZ   38     0.997 0.000999001
## 3     NKI   51     0.896 0.000999001
## 4     UPP   34     0.912 0.000999001
## 5     UNT   40     1.000 0.000999001
```
