# Supplementary figures and images for: Signpost Testing to Navigate the Parameter Space of the Gaussian Graphical Model With High‐Dimensional Data
Source: Biom J. 2026 Feb 12;68(1):e70115. doi: 10.1002/bimj.70115 (PMC12895234; doi:10.1002/bimj.70115)

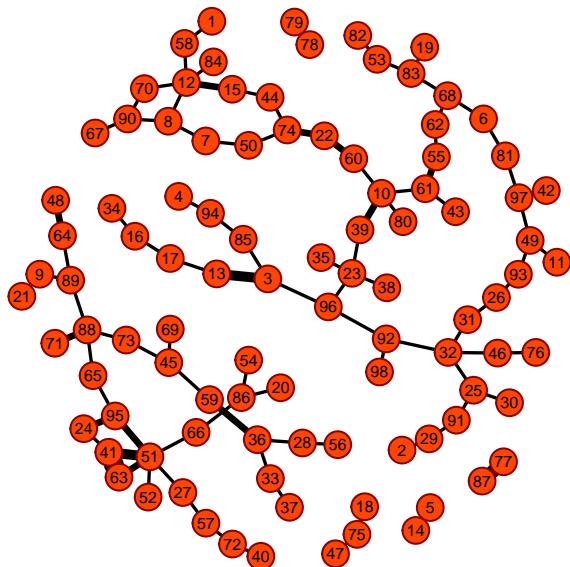

Supplement: Supplementary file 1 — Supporting File: bimj70115‐sup‐0001‐Datacode.zip. [file BIMJ-68-e70115-s001.zip › code and data/plot/cond_ind_graphsOmega.pdf]

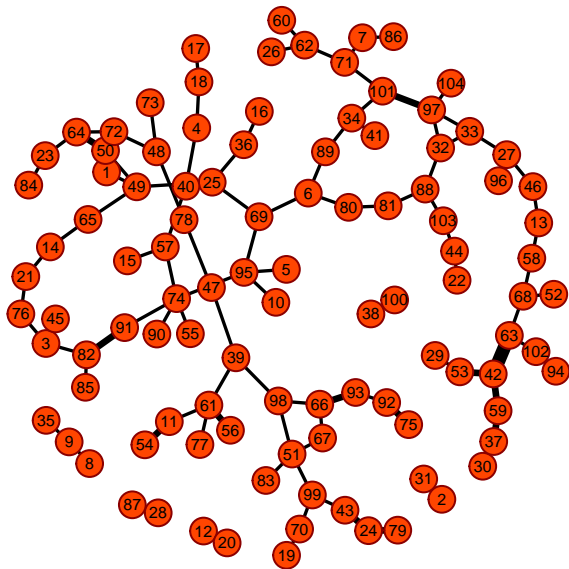

Supplement: Supplementary file 1 — Supporting File: bimj70115‐sup‐0001‐Datacode.zip. [file BIMJ-68-e70115-s001.zip › code and data/plot/cond_ind_graphsT0.pdf]

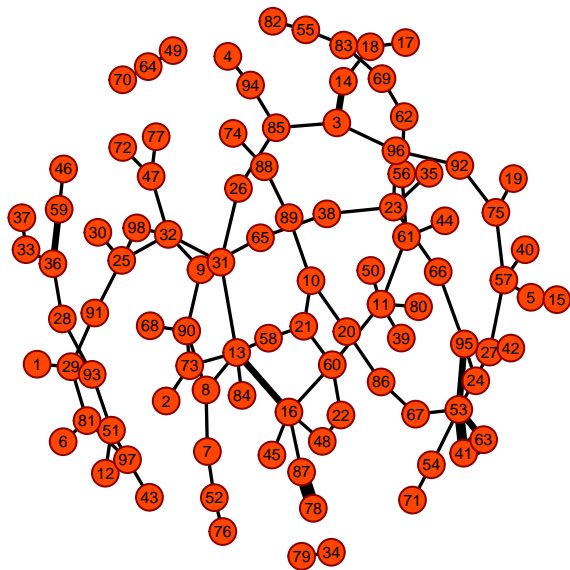

Supplement: Supplementary file 1 — Supporting File: bimj70115‐sup‐0001‐Datacode.zip. [file BIMJ-68-e70115-s001.zip › code and data/plot/cond_ind_graphsTa.pdf]

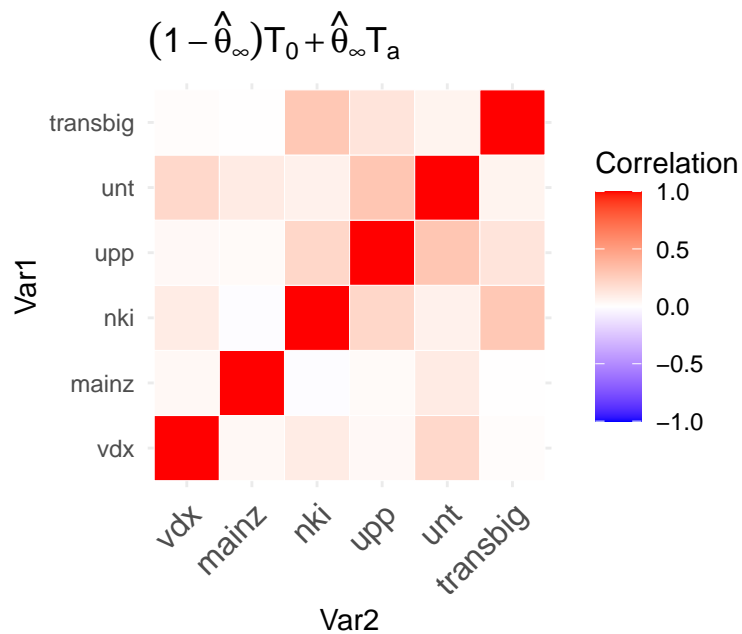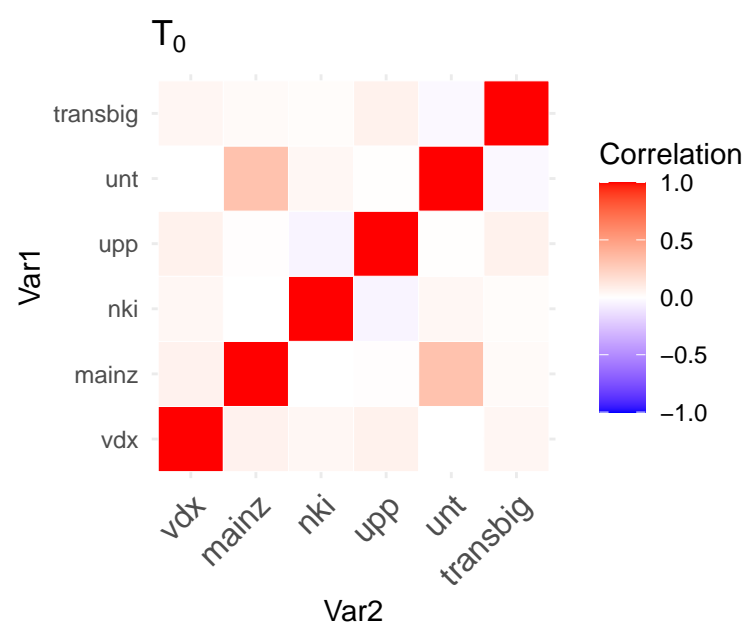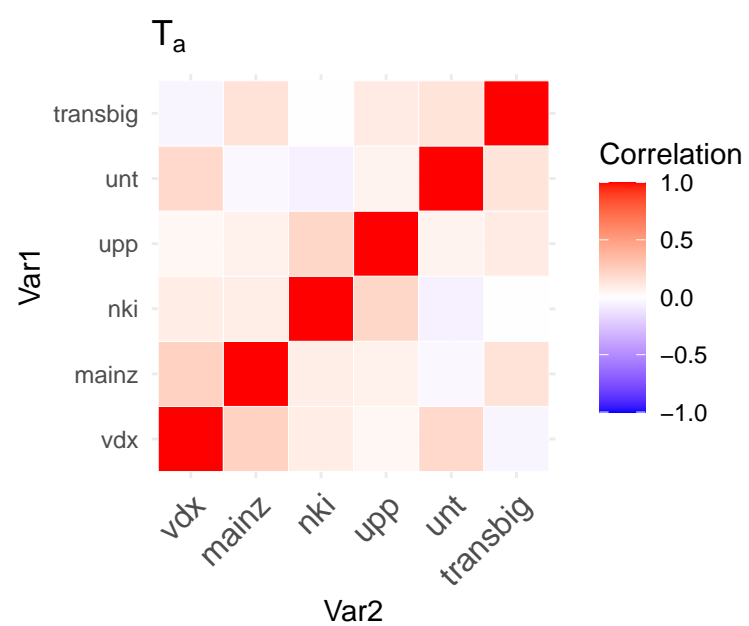

Supplement: Supplementary file 1 — Supporting File: bimj70115‐sup‐0001‐Datacode.zip. [file BIMJ-68-e70115-s001.zip › code and data/plot/cor_top100_spearman.pdf]

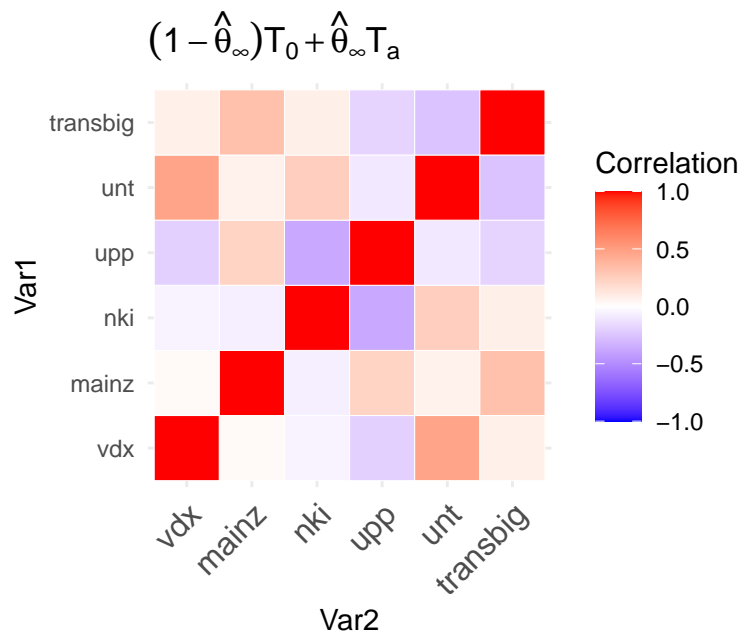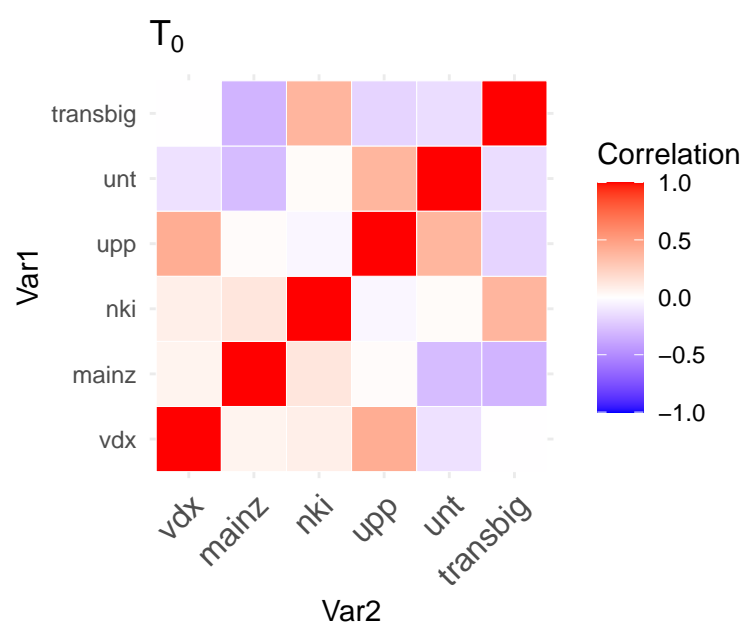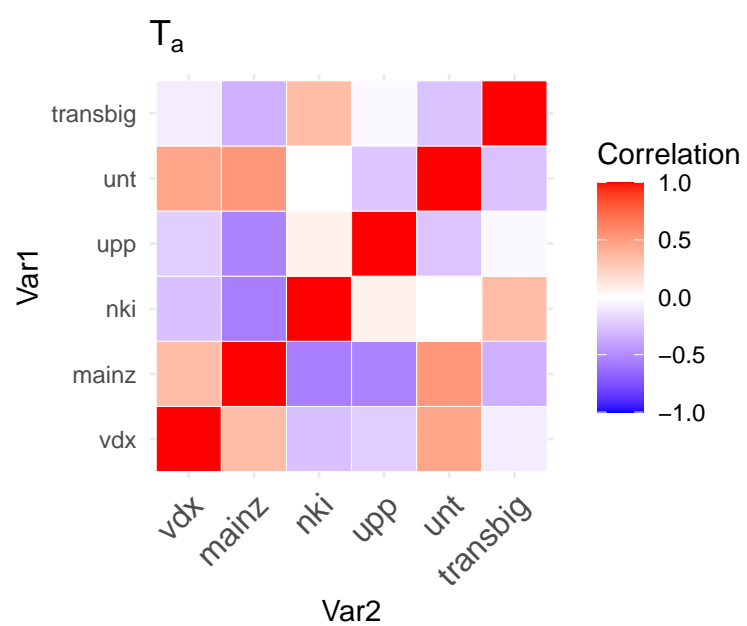

Supplement: Supplementary file 1 — Supporting File: bimj70115‐sup‐0001‐Datacode.zip. [file BIMJ-68-e70115-s001.zip › code and data/plot/cor_top20_spearman.pdf]

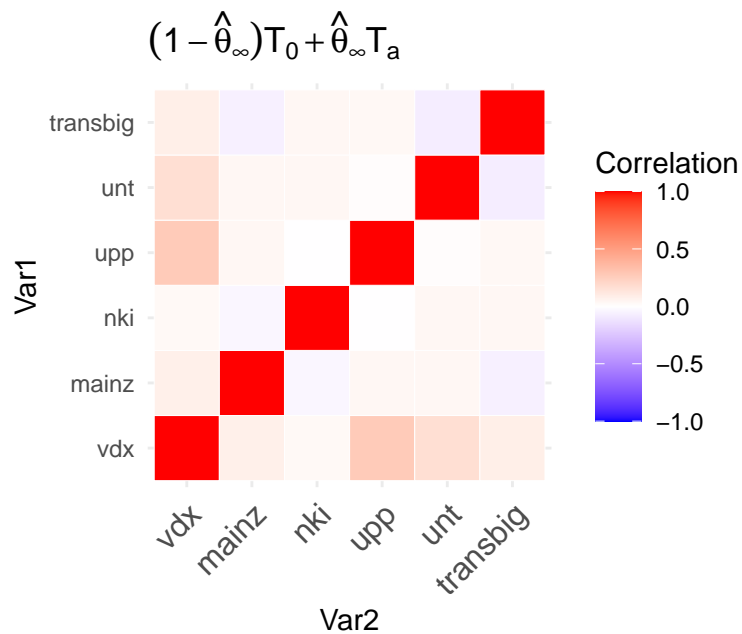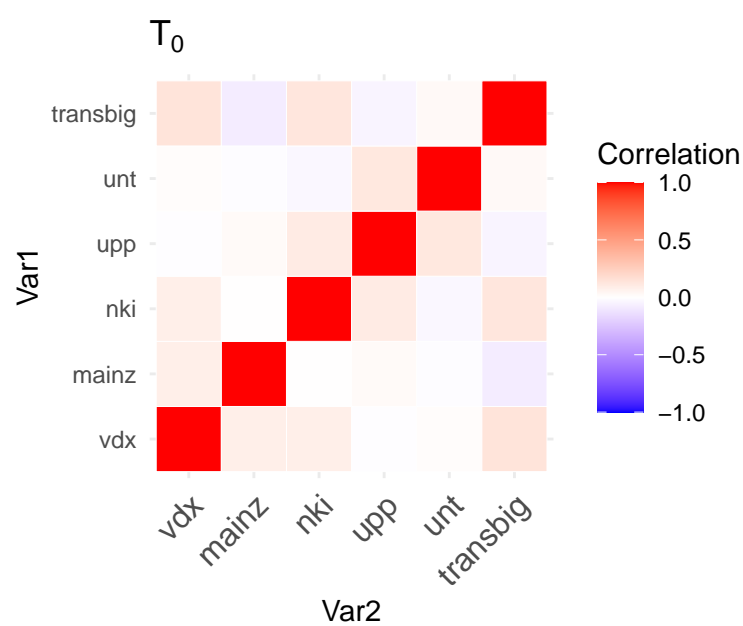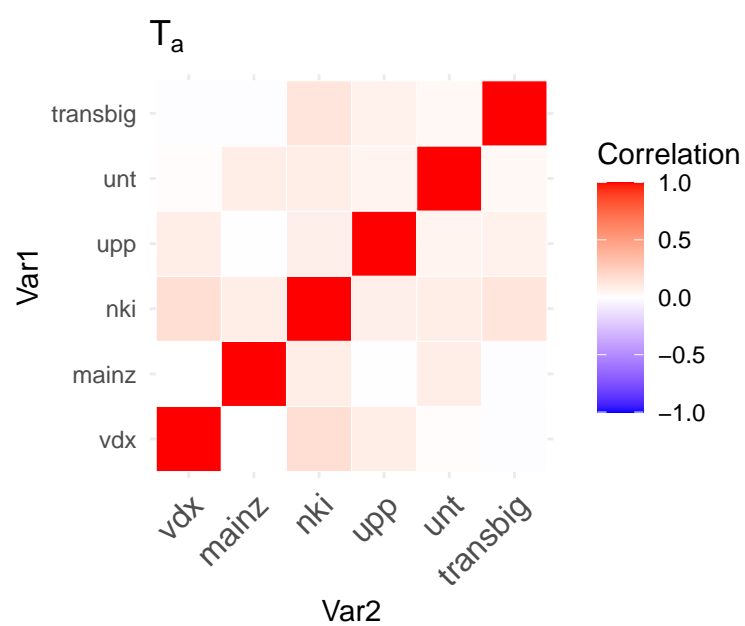

Supplement: Supplementary file 1 — Supporting File: bimj70115‐sup‐0001‐Datacode.zip. [file BIMJ-68-e70115-s001.zip › code and data/plot/cor_top200_spearman.pdf]

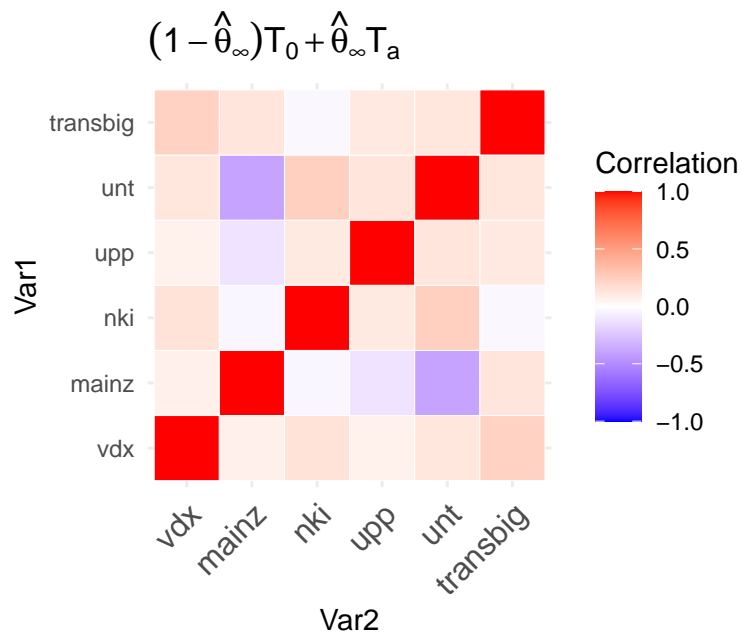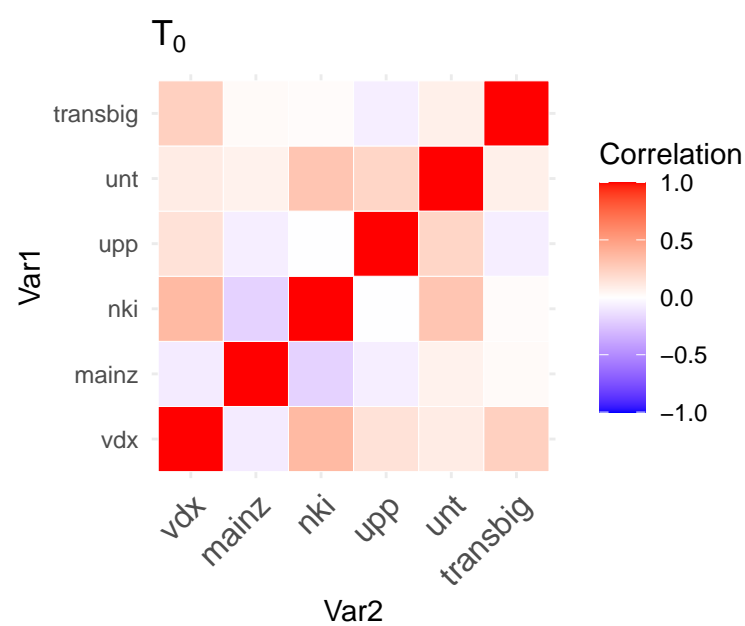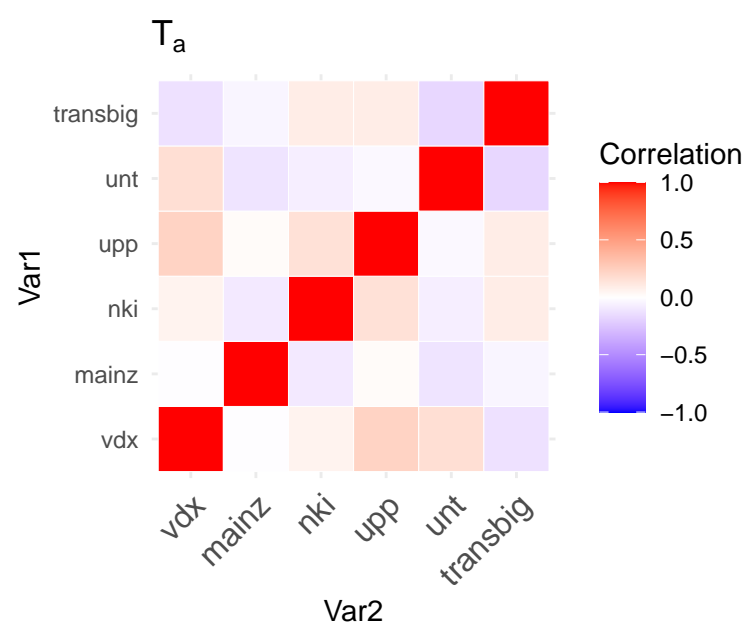

Supplement: Supplementary file 1 — Supporting File: bimj70115‐sup‐0001‐Datacode.zip. [file BIMJ-68-e70115-s001.zip › code and data/plot/cor_top50_spearman.pdf]

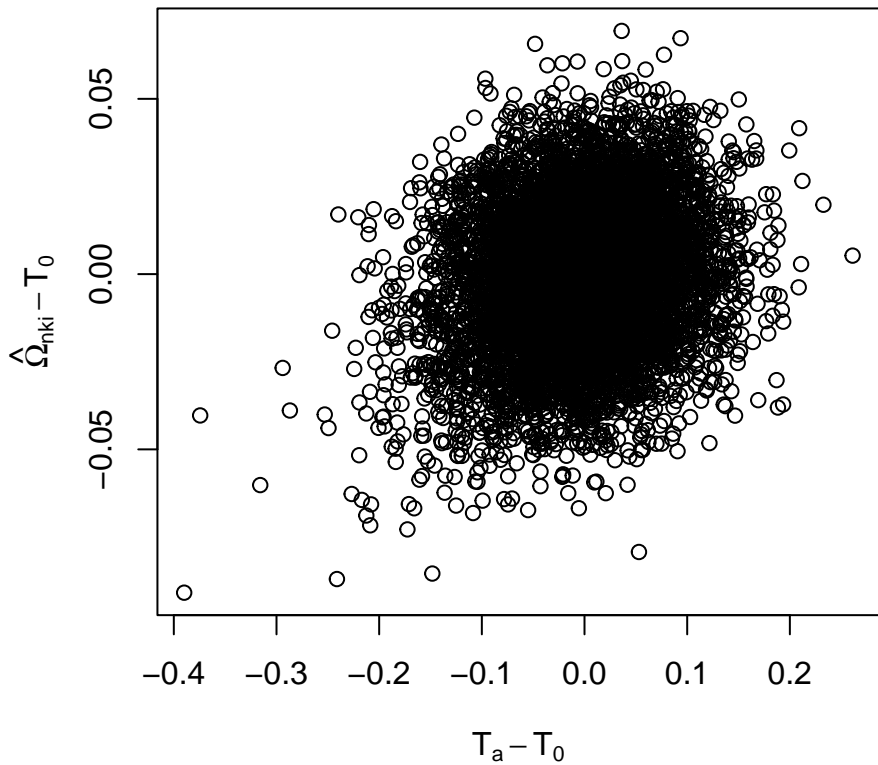

Supplement: Supplementary file 1 — Supporting File: bimj70115‐sup‐0001‐Datacode.zip. [file BIMJ-68-e70115-s001.zip › code and data/plot/Diagnostic_plot_left.pdf]

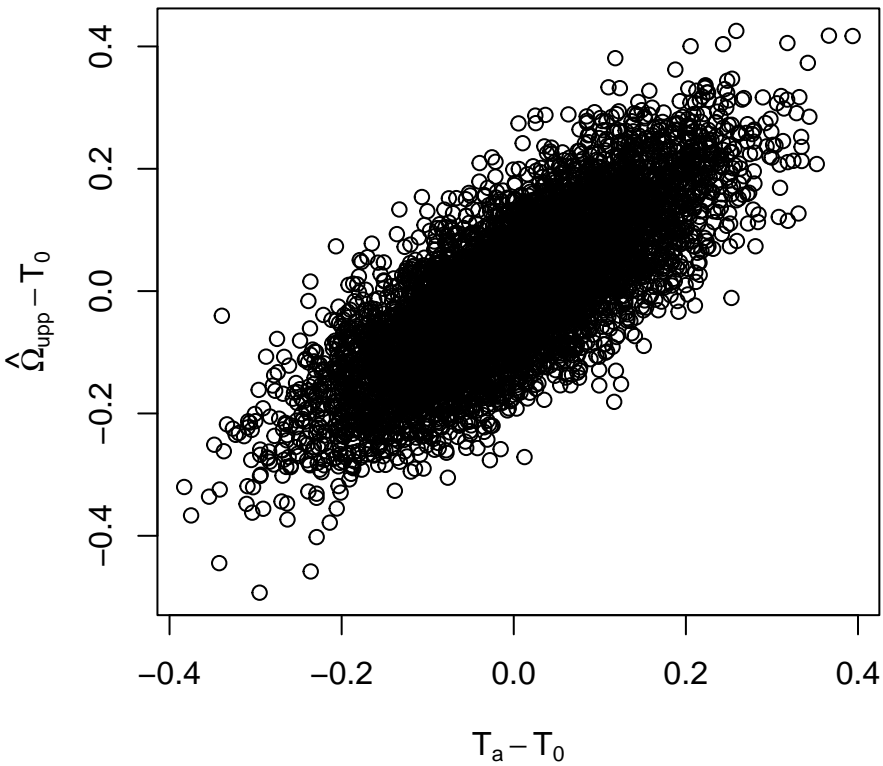

Supplement: Supplementary file 1 — Supporting File: bimj70115‐sup‐0001‐Datacode.zip. [file BIMJ-68-e70115-s001.zip › code and data/plot/Diagnostic_plot_right.pdf]

Hellinger Distance

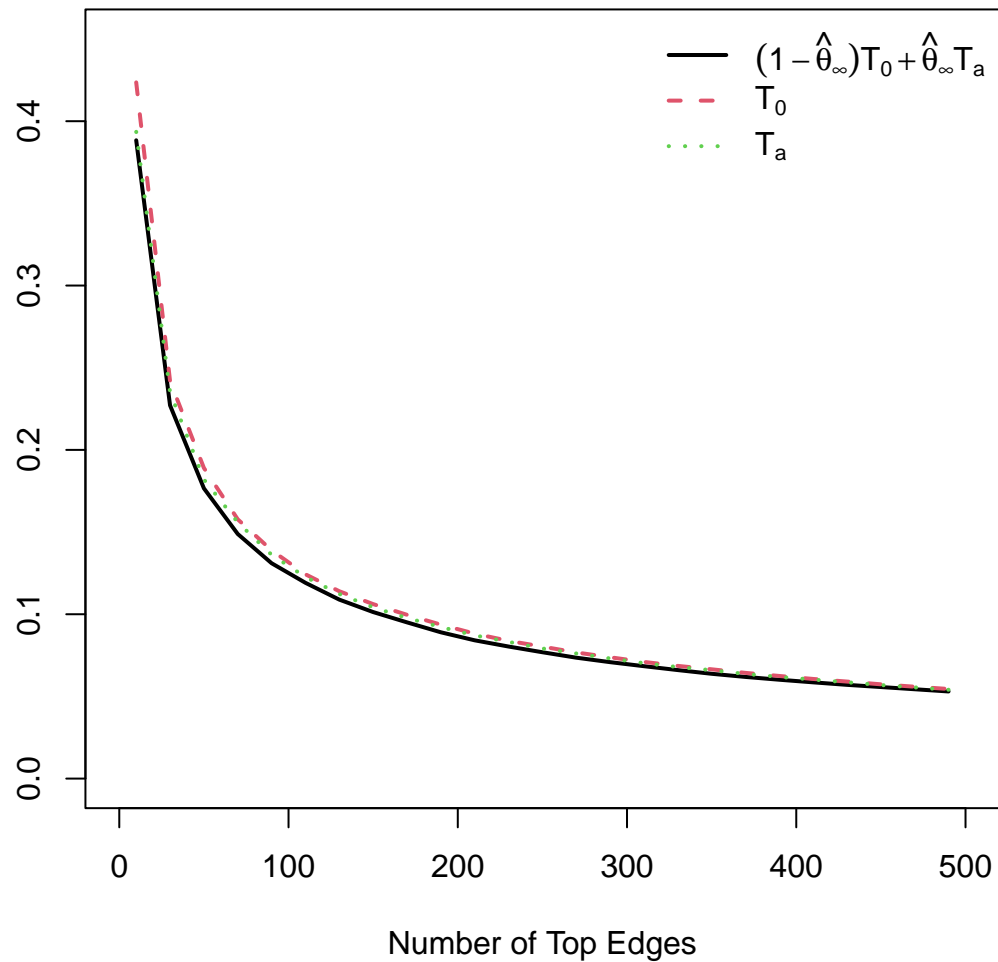

Supplement: Supplementary file 1 — Supporting File: bimj70115‐sup‐0001‐Datacode.zip. [file BIMJ-68-e70115-s001.zip › code and data/plot/distance_500.pdf]

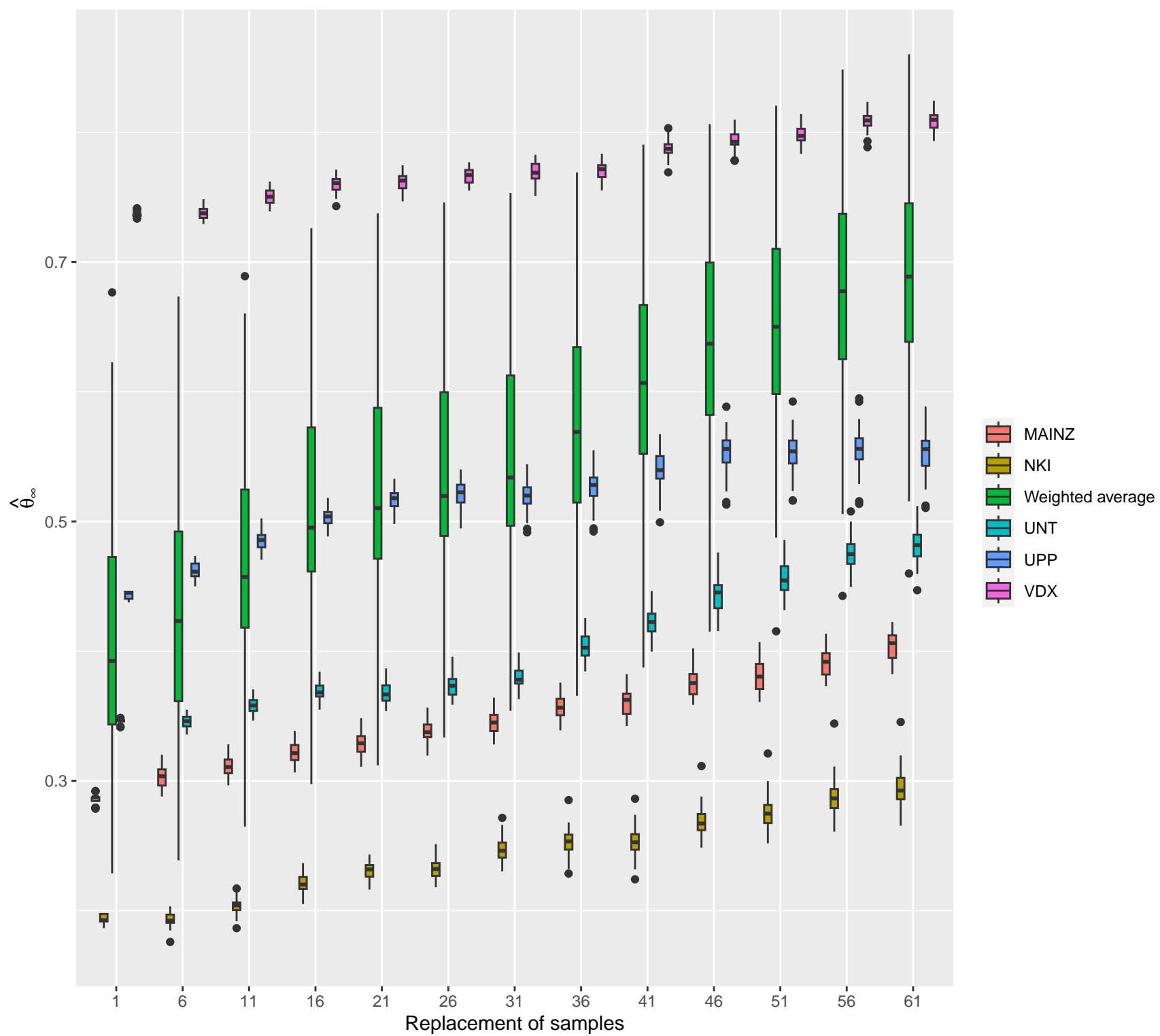

Supplement: Supplementary file 1 — Supporting File: bimj70115‐sup‐0001‐Datacode.zip. [file BIMJ-68-e70115-s001.zip › code and data/plot/empirical_validity.pdf]

Loss,  $T_a$ : transbig ER-, RidgeP

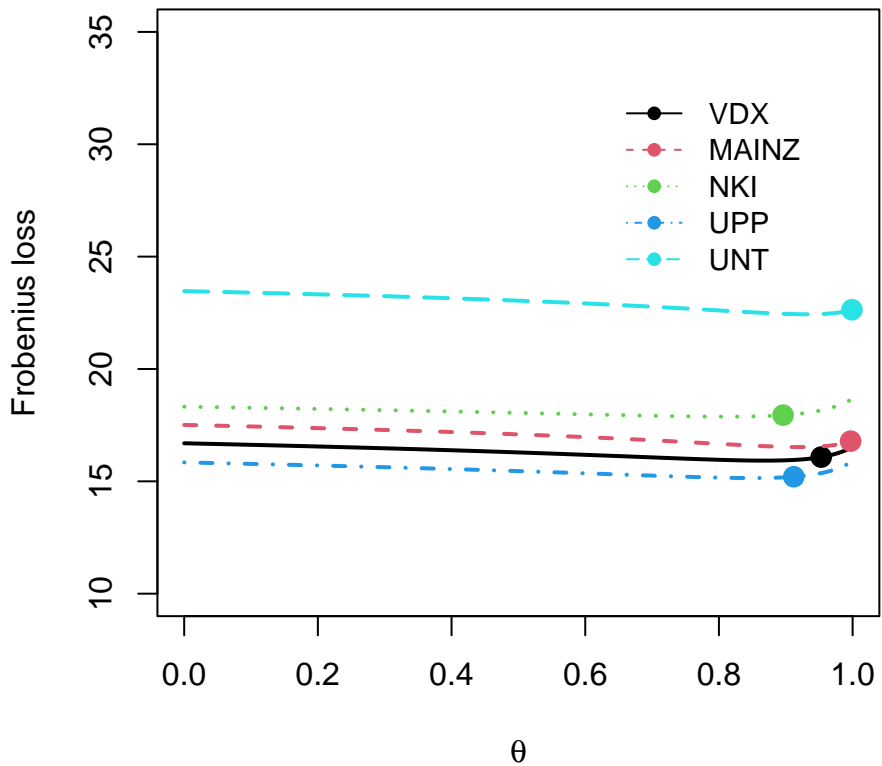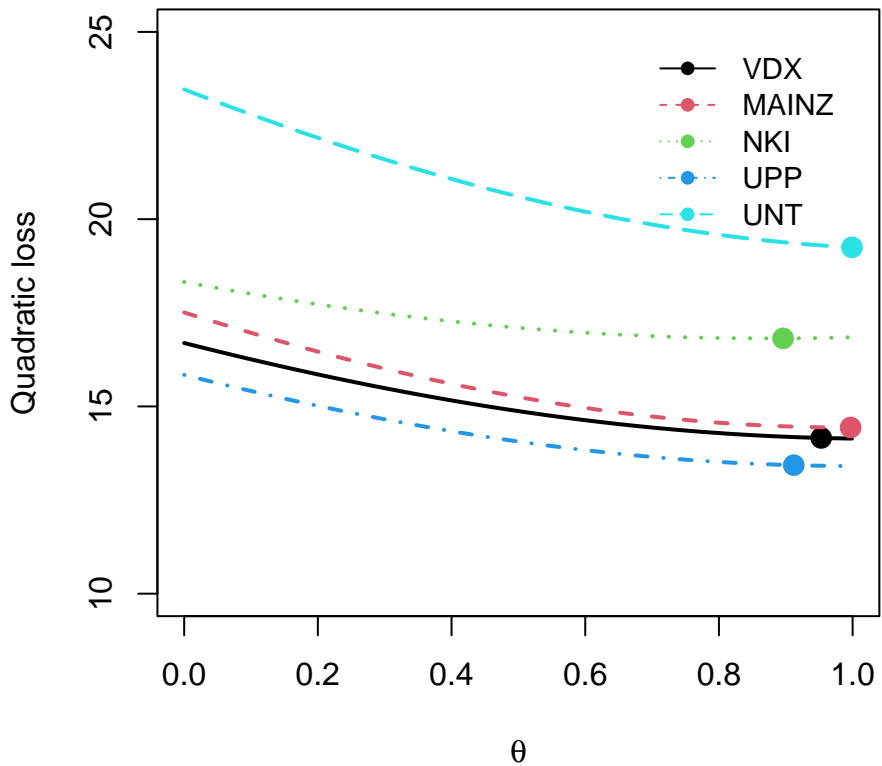

Supplement: Supplementary file 1 — Supporting File: bimj70115‐sup‐0001‐Datacode.zip. [file BIMJ-68-e70115-s001.zip › code and data/plot/fed_learning_lossVStheta.pdf]

scale-free inverse,  $p = 50$ ,  $\lambda = 10$

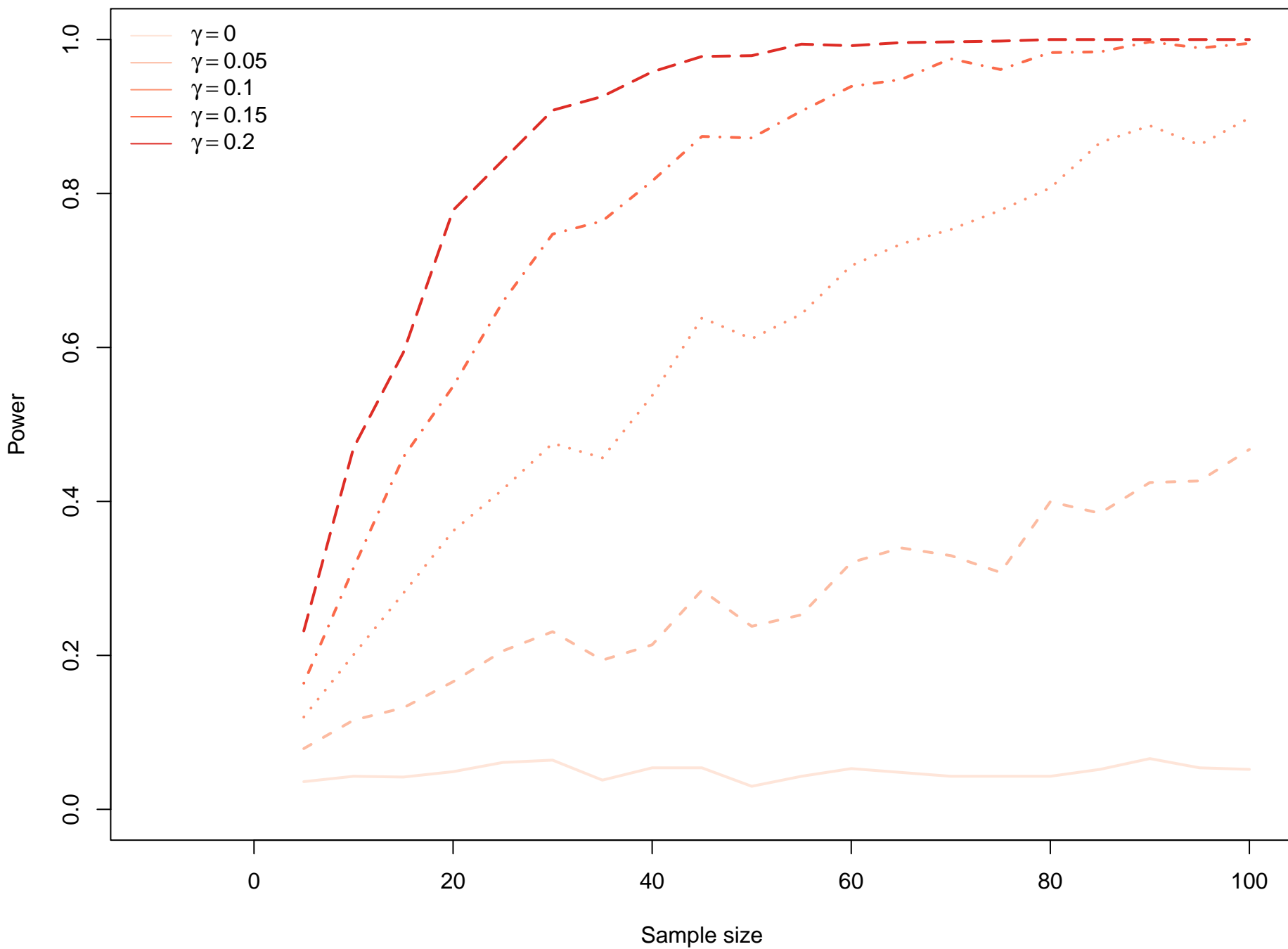

Supplement: Supplementary file 1 — Supporting File: bimj70115‐sup‐0001‐Datacode.zip. [file BIMJ-68-e70115-s001.zip › code and data/plot/general_powerplot_SFinv_p50_lambda10.pdf]

# Frequency Density of $\theta$ given $\gamma = 0$ , $n = 50$ , $\mathbf{T}_a = \text{scale-free inverse}$

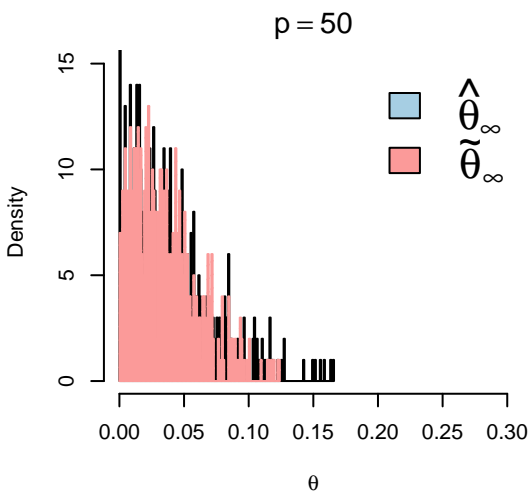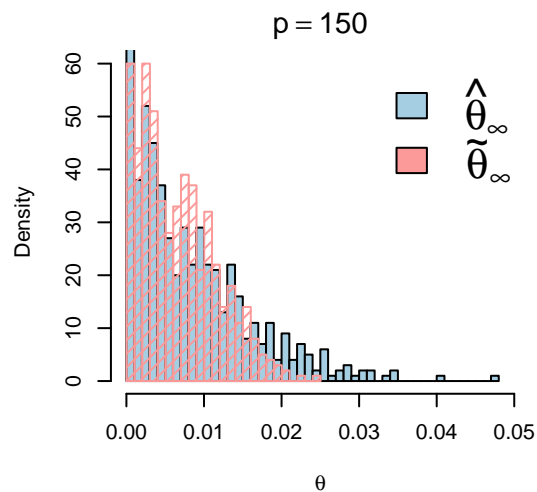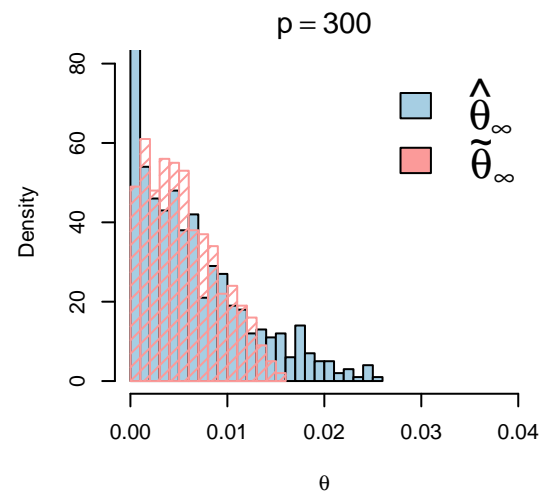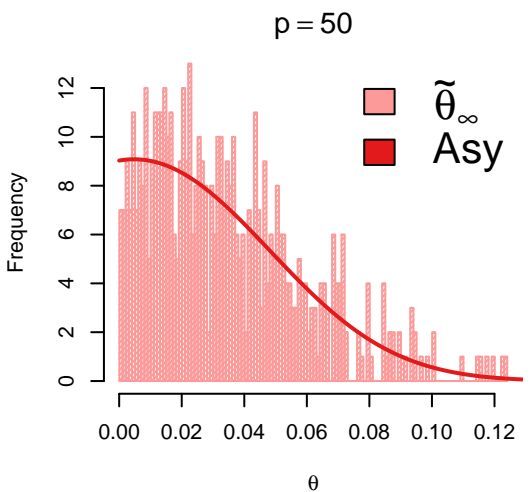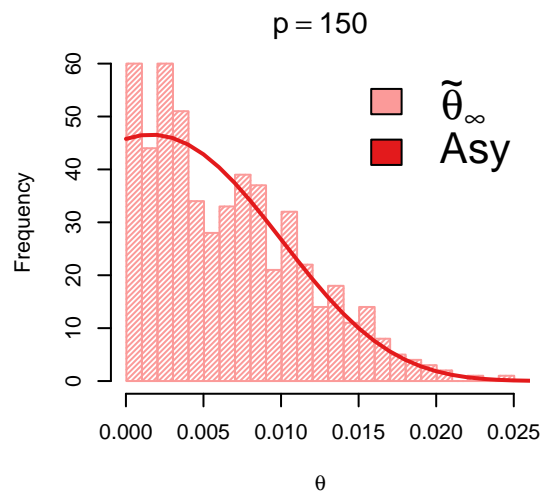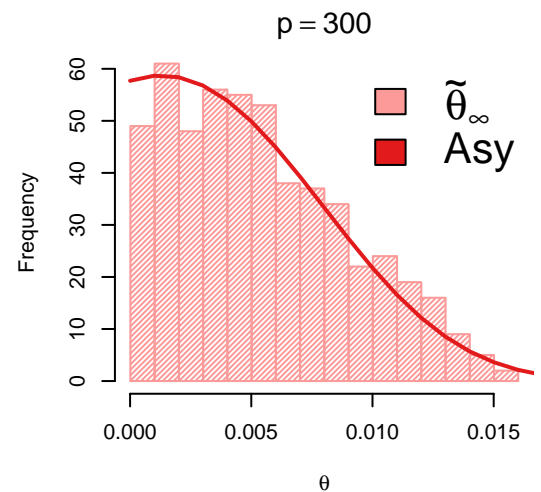

Supplement: Supplementary file 1 — Supporting File: bimj70115‐sup‐0001‐Datacode.zip. [file BIMJ-68-e70115-s001.zip › code and data/plot/hist_SFinv_n50.pdf]

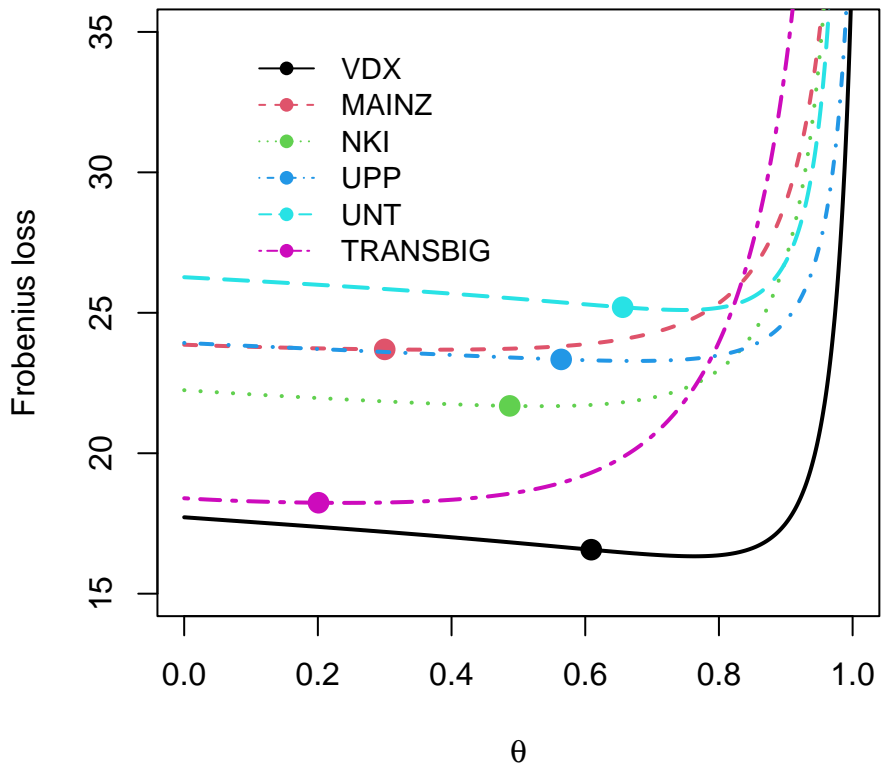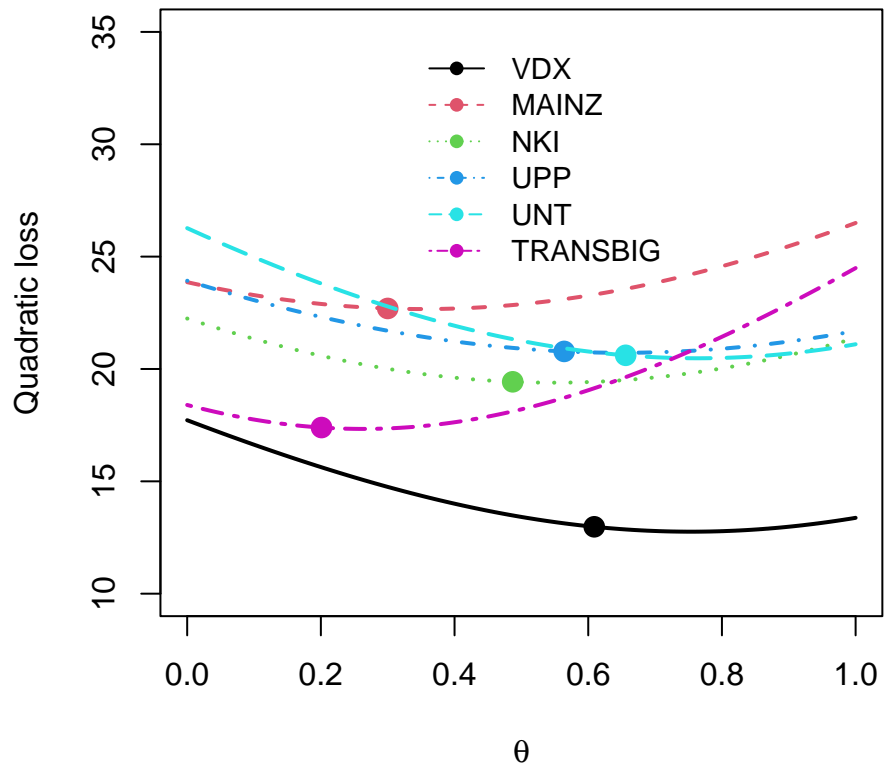

Supplement: Supplementary file 1 — Supporting File: bimj70115‐sup‐0001‐Datacode.zip. [file BIMJ-68-e70115-s001.zip › code and data/plot/losses_6.2.pdf]

**uniform, topology**

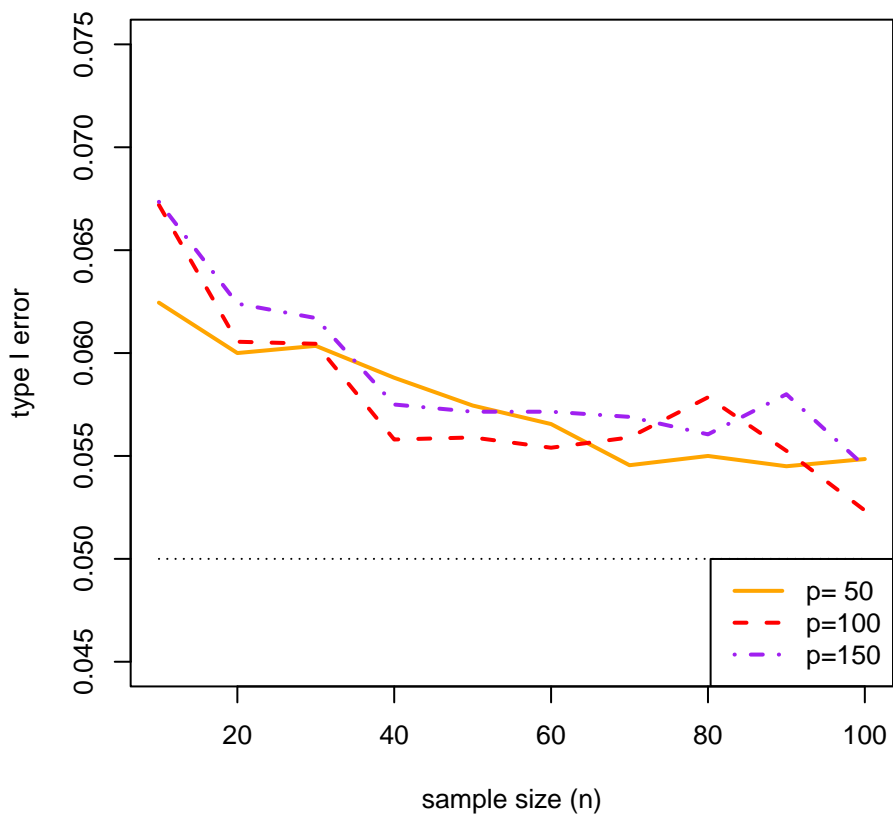

**bandedInv, topology**

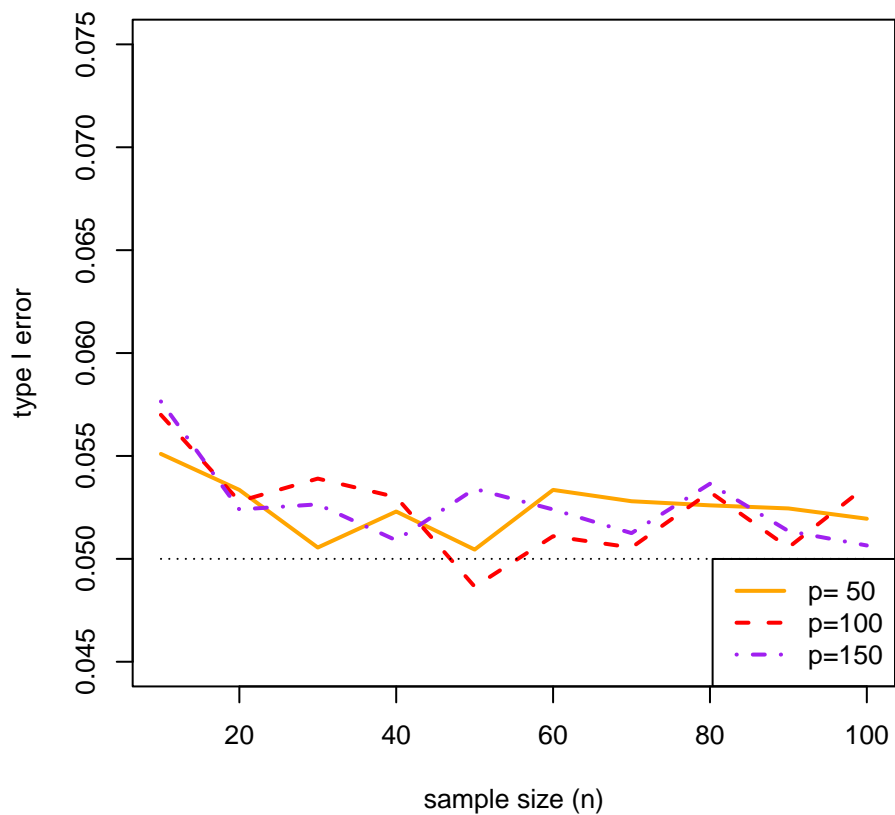

**stripInv, topology**

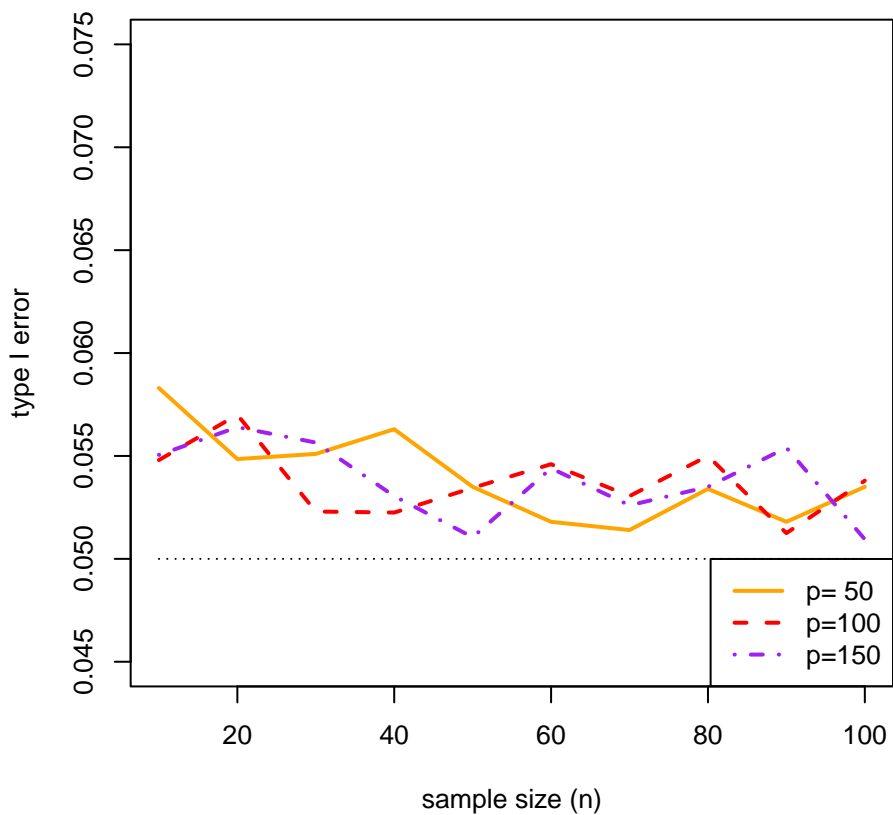

**ScaleFreeInv, topology**

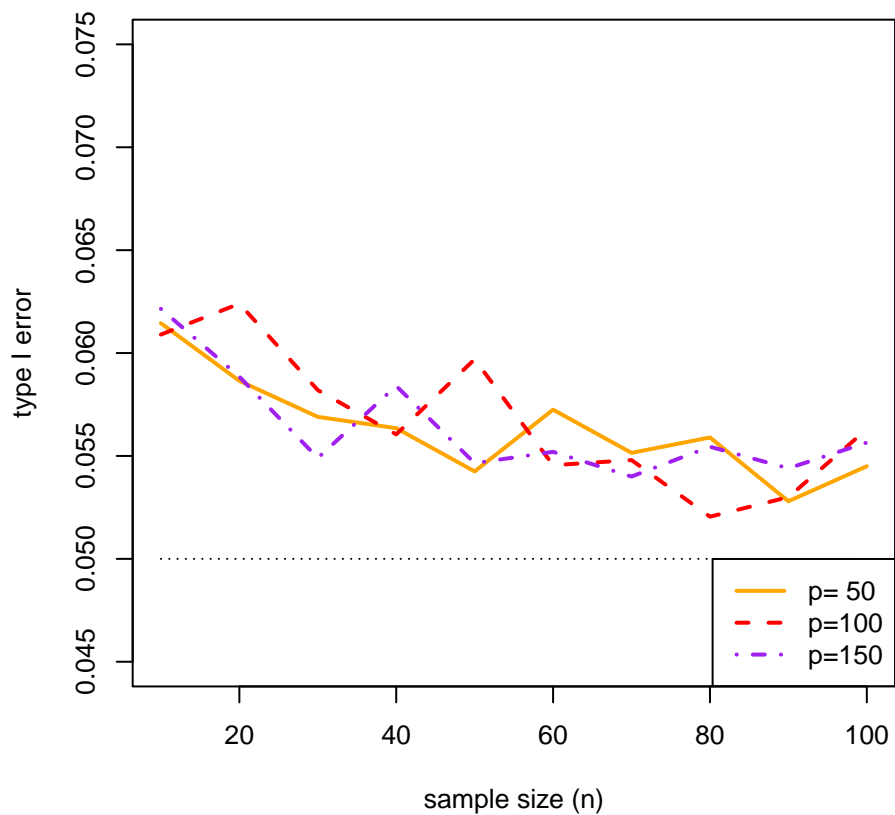

Supplement: Supplementary file 1 — Supporting File: bimj70115‐sup‐0001‐Datacode.zip. [file BIMJ-68-e70115-s001.zip › code and data/plot/lrt_typeIerror.pdf]

$T_a$  = banded inverse,  $p = 150$

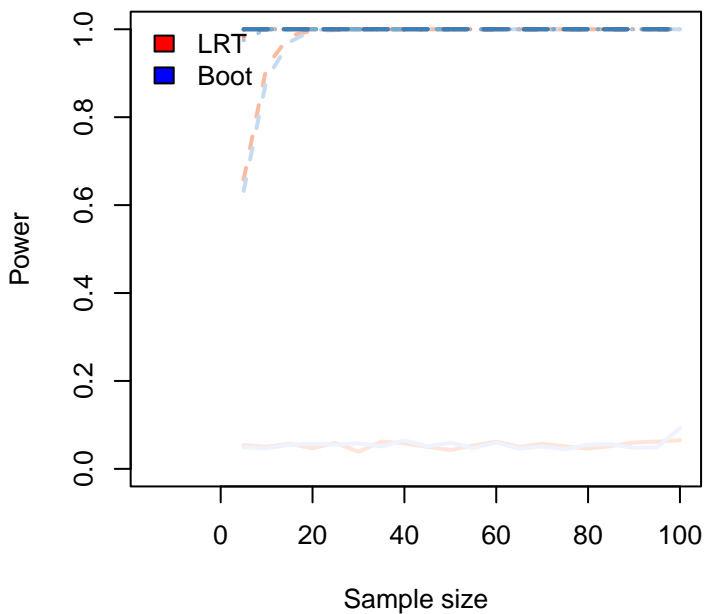

$T_a$  = scale-free inverse,  $p = 150$

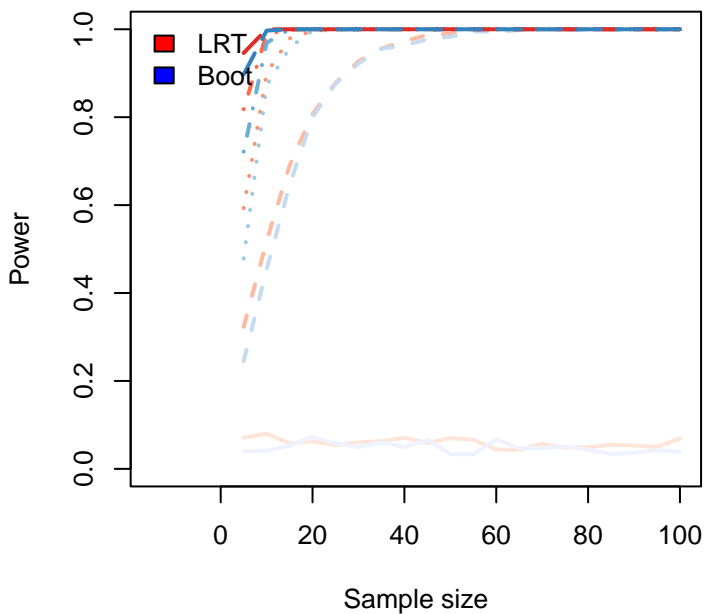

$T_a$  = stripe inverse,  $p = 150$

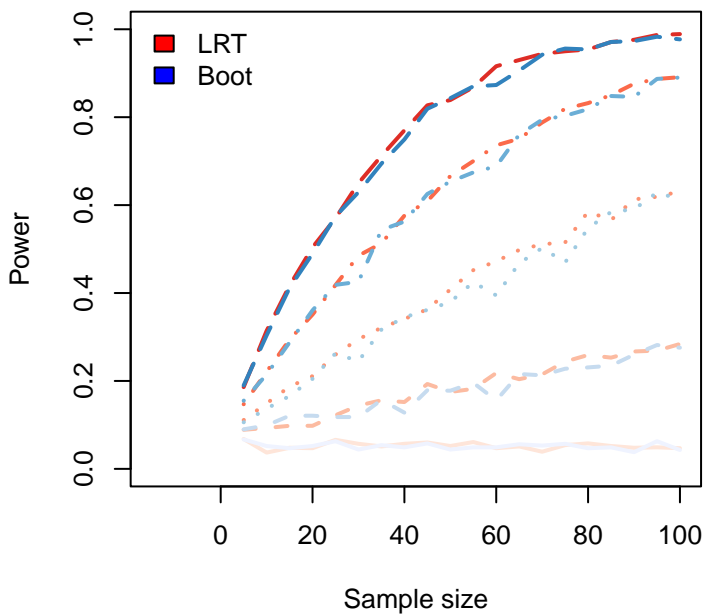

$T_a$  = uniform,  $p = 150$

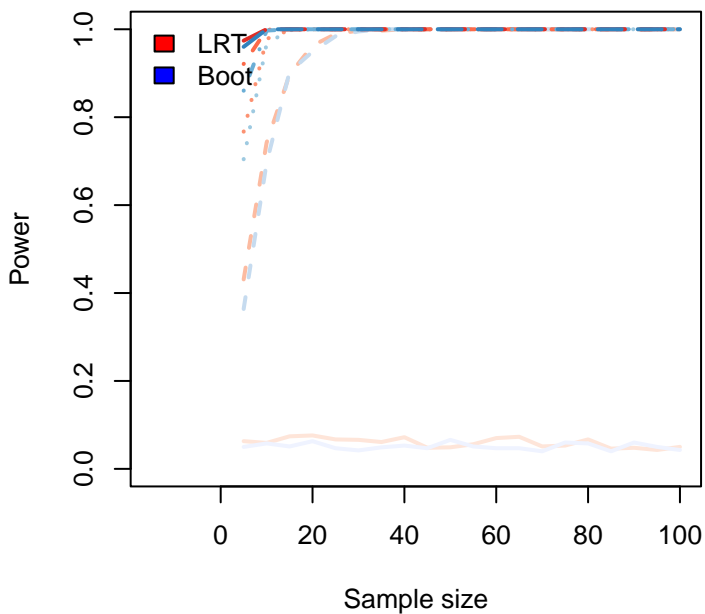

Supplement: Supplementary file 1 — Supporting File: bimj70115‐sup‐0001‐Datacode.zip. [file BIMJ-68-e70115-s001.zip › code and data/plot/lrtVSBoot_powerplot.pdf]

scale-free inverse,  $p = 50$

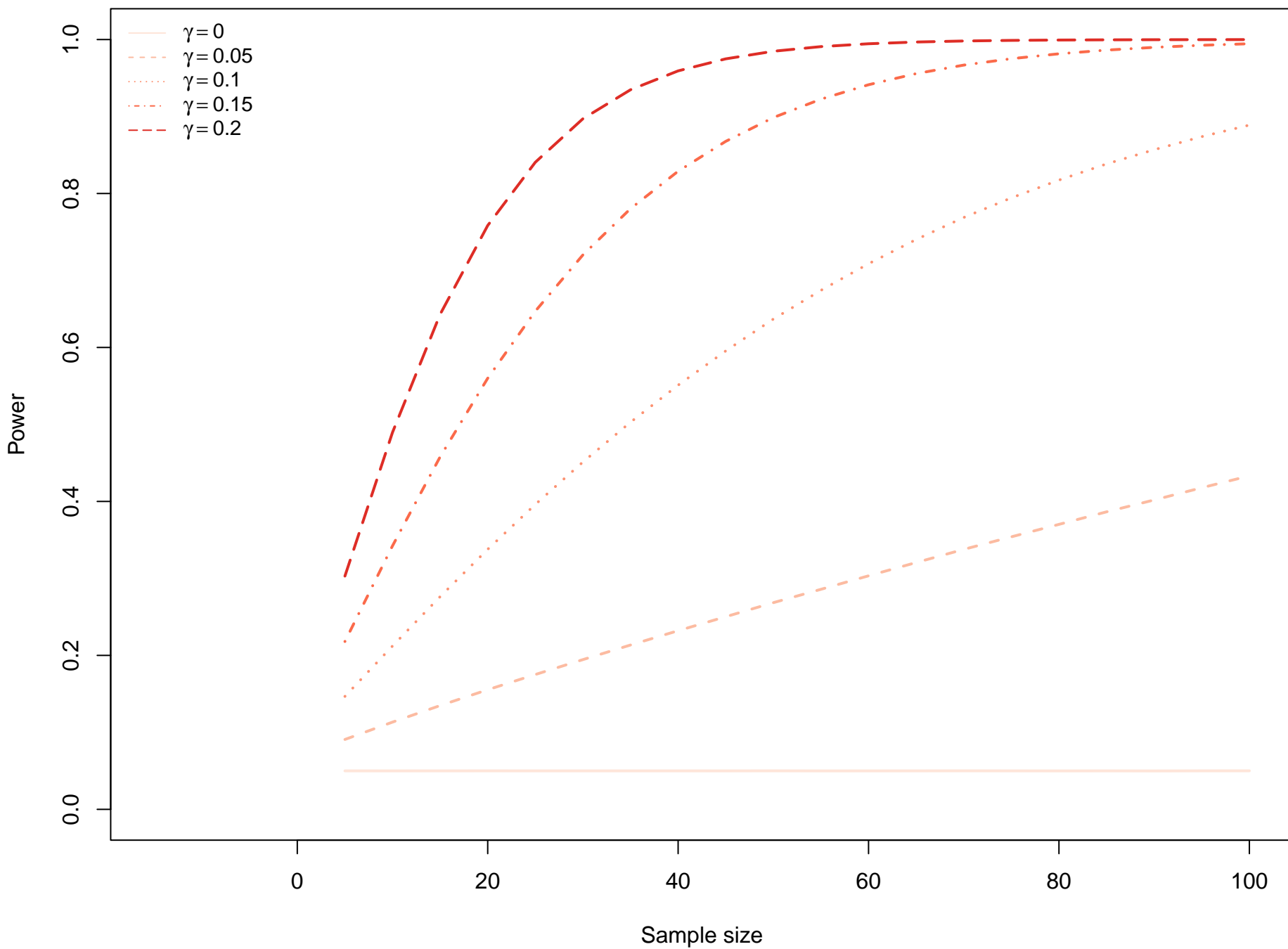

Supplement: Supplementary file 1 — Supporting File: bimj70115‐sup‐0001‐Datacode.zip. [file BIMJ-68-e70115-s001.zip › code and data/plot/powerplot_SFinv_approx_p50.pdf]

scale-free inverse,  $p = 50$

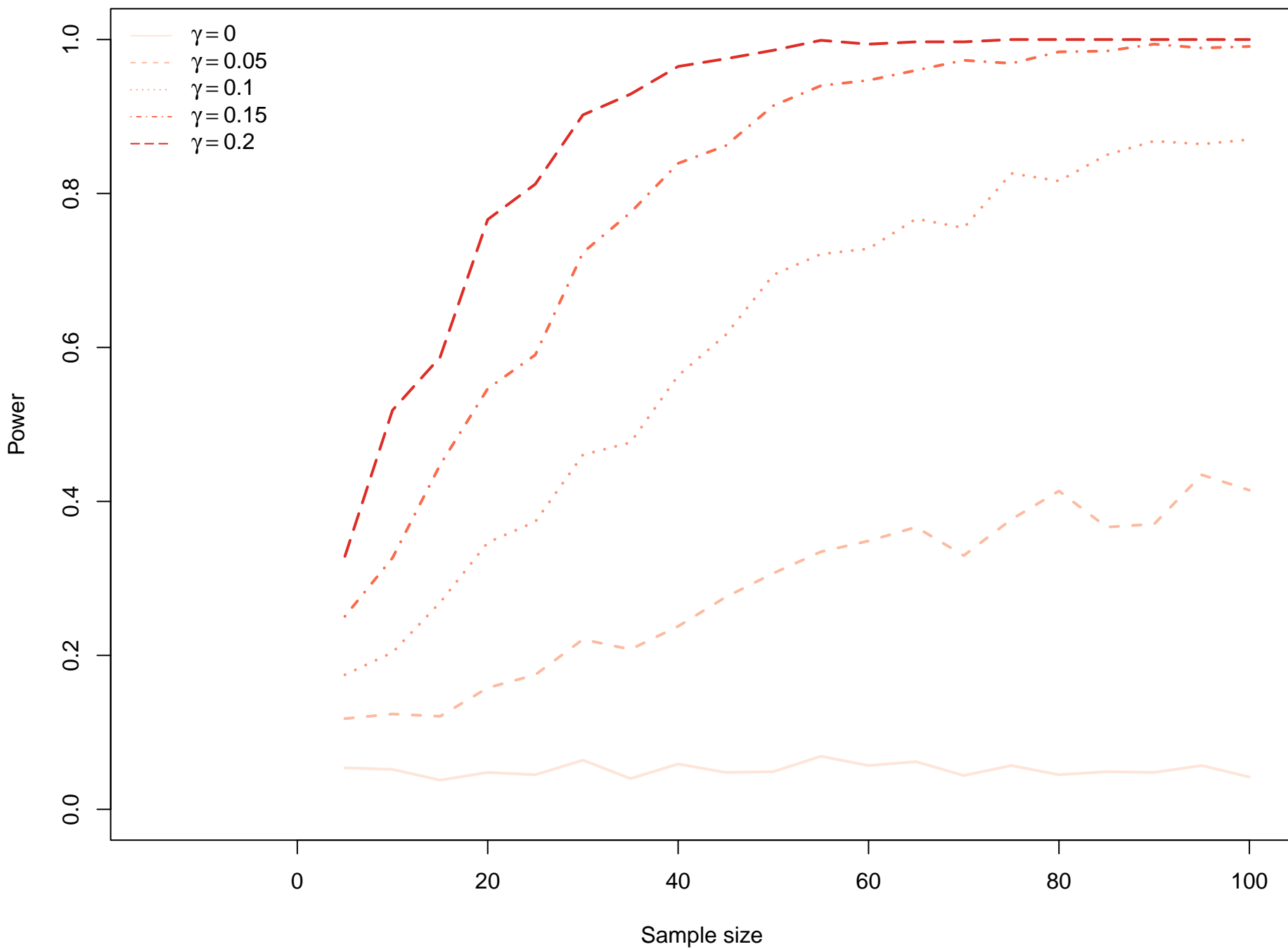

Supplement: Supplementary file 1 — Supporting File: bimj70115‐sup‐0001‐Datacode.zip. [file BIMJ-68-e70115-s001.zip › code and data/plot/powerplot_SFinv_p50.pdf]

Quadratic loss

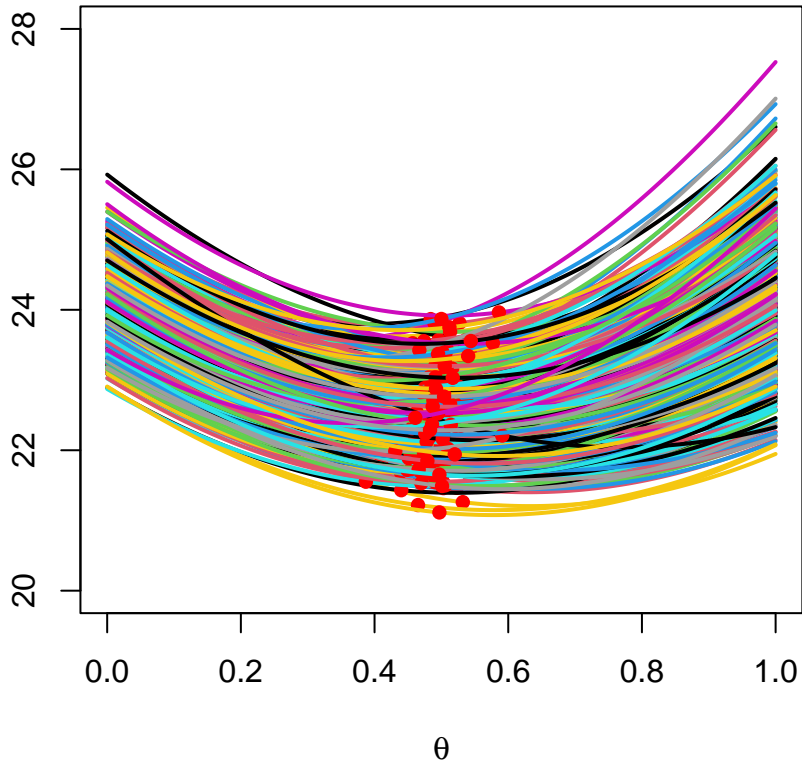

Supplement: Supplementary file 1 — Supporting File: bimj70115‐sup‐0001‐Datacode.zip. [file BIMJ-68-e70115-s001.zip › code and data/plot/QLossVStheta_n50p150_gamma05.pdf]

Quadratic loss

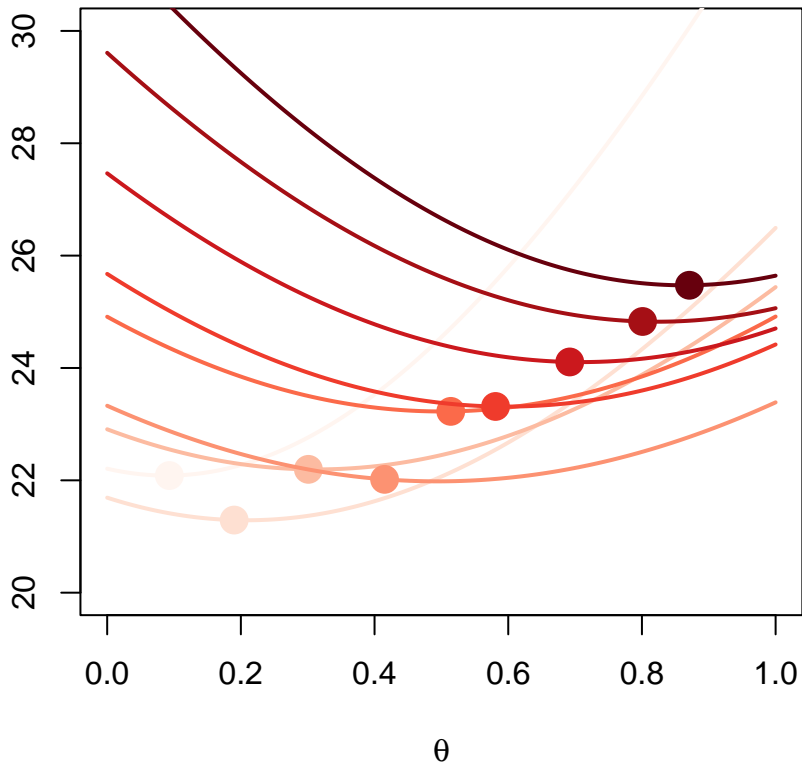

Supplement: Supplementary file 1 — Supporting File: bimj70115‐sup‐0001‐Datacode.zip. [file BIMJ-68-e70115-s001.zip › code and data/plot/QLossVStheta_n50p150_gammavary.pdf]

$\hat{\theta}$  vs  $\tilde{\theta}$  Topology = scale – free inverse

$n = 50, p = 50$

$n = 50, p = 150$

$n = 50, p = 300$

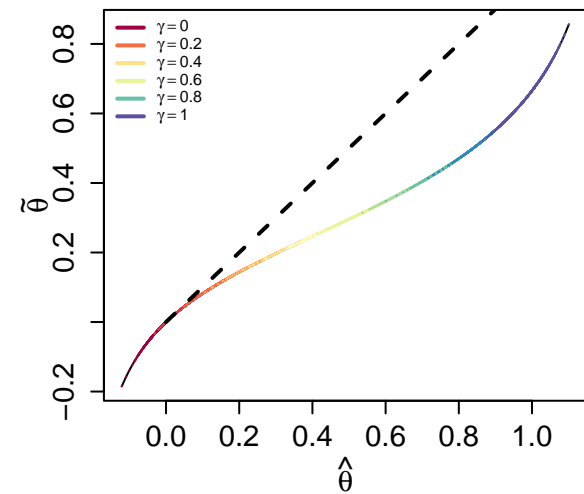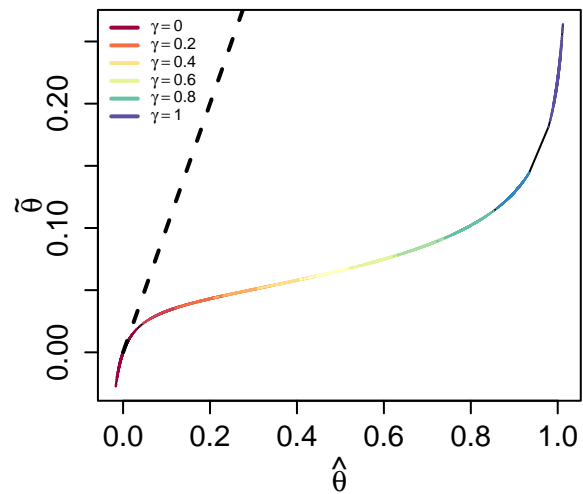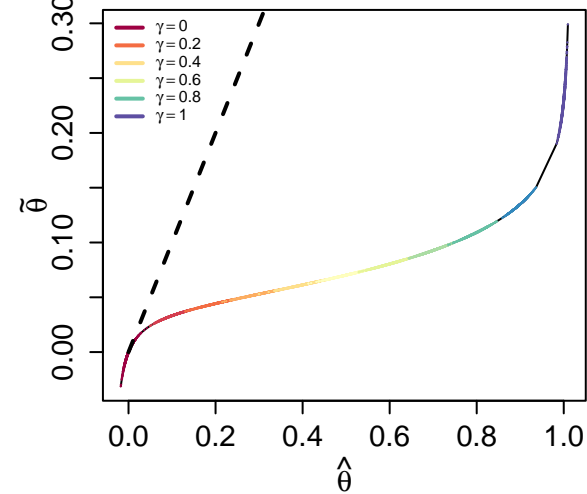

$n = 100, p = 50$

$n = 100, p = 150$

$n = 100, p = 300$

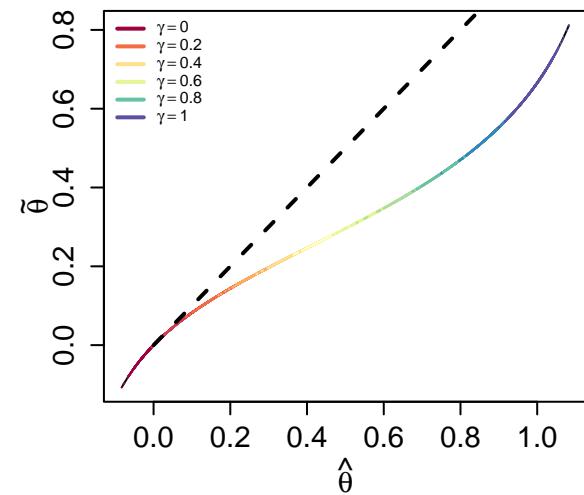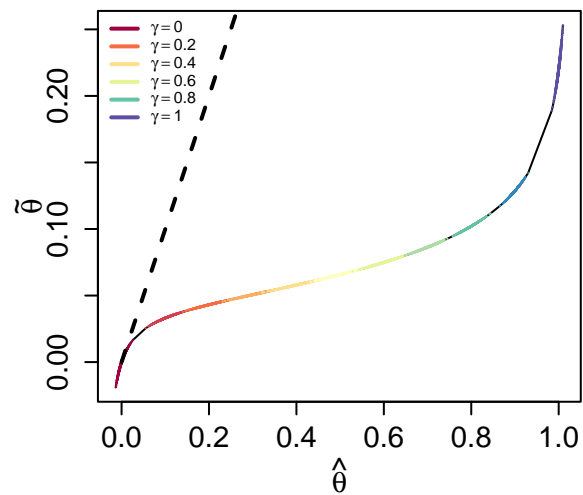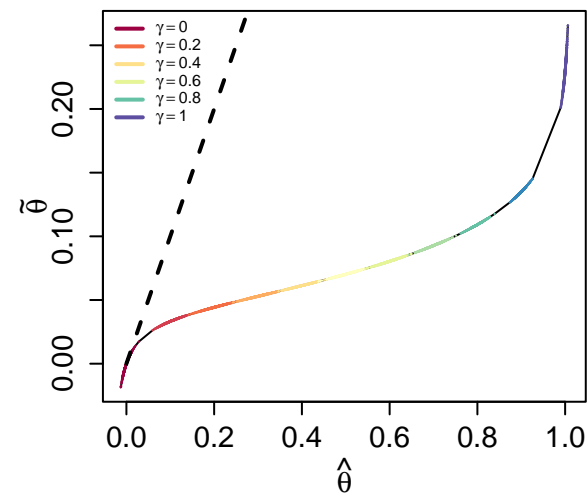

Supplement: Supplementary file 1 — Supporting File: bimj70115‐sup‐0001‐Datacode.zip. [file BIMJ-68-e70115-s001.zip › code and data/plot/qqplot_Test_VS_Approximated.pdf]

Regularization path, topology = scale – free inverse, n = 50, p = 50

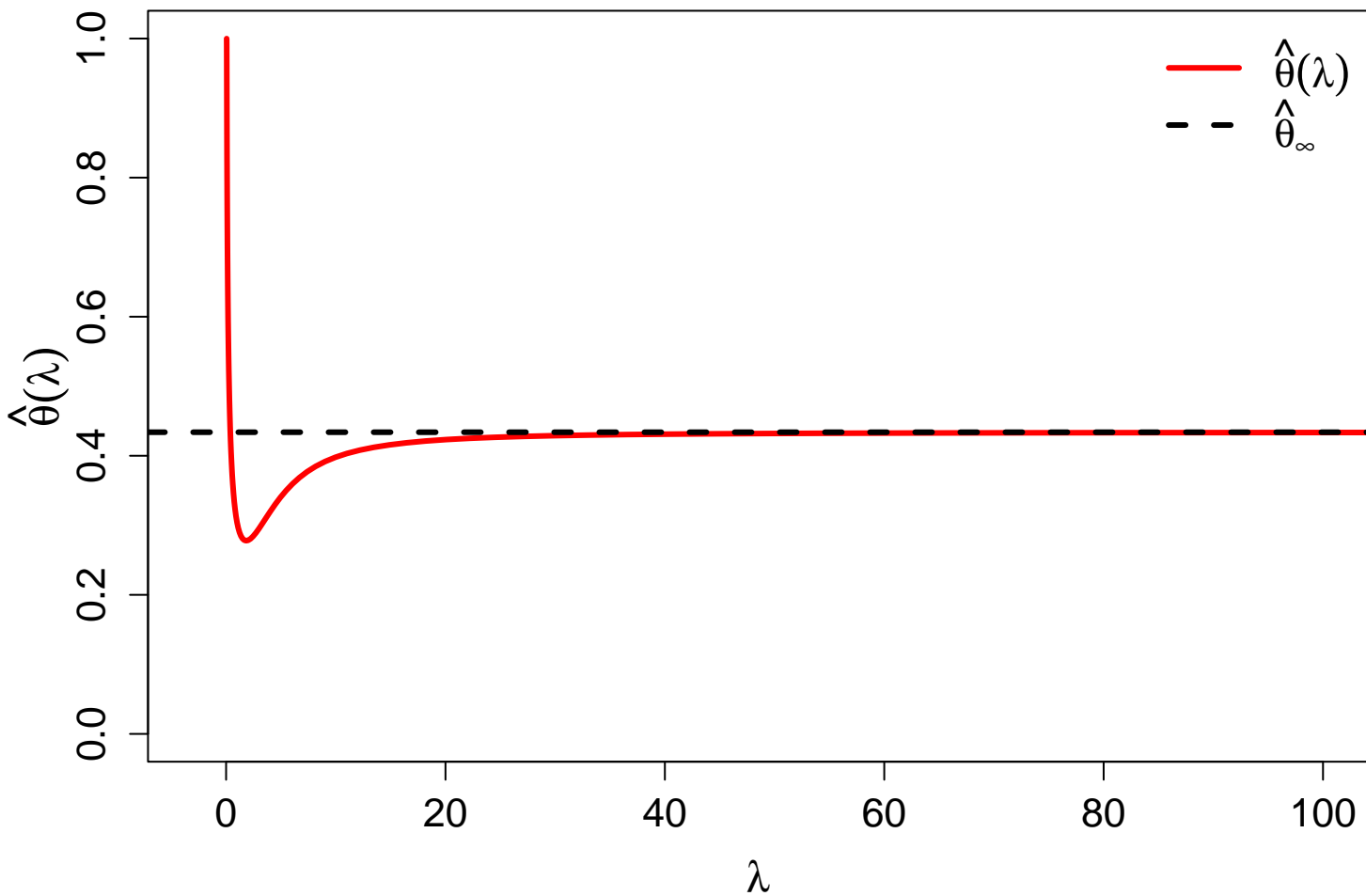

Supplement: Supplementary file 1 — Supporting File: bimj70115‐sup‐0001‐Datacode.zip. [file BIMJ-68-e70115-s001.zip › code and data/plot/regularization_path_SFinv_n=50_p=50.pdf]

Loss,  $T_a$ : Generalized inverse

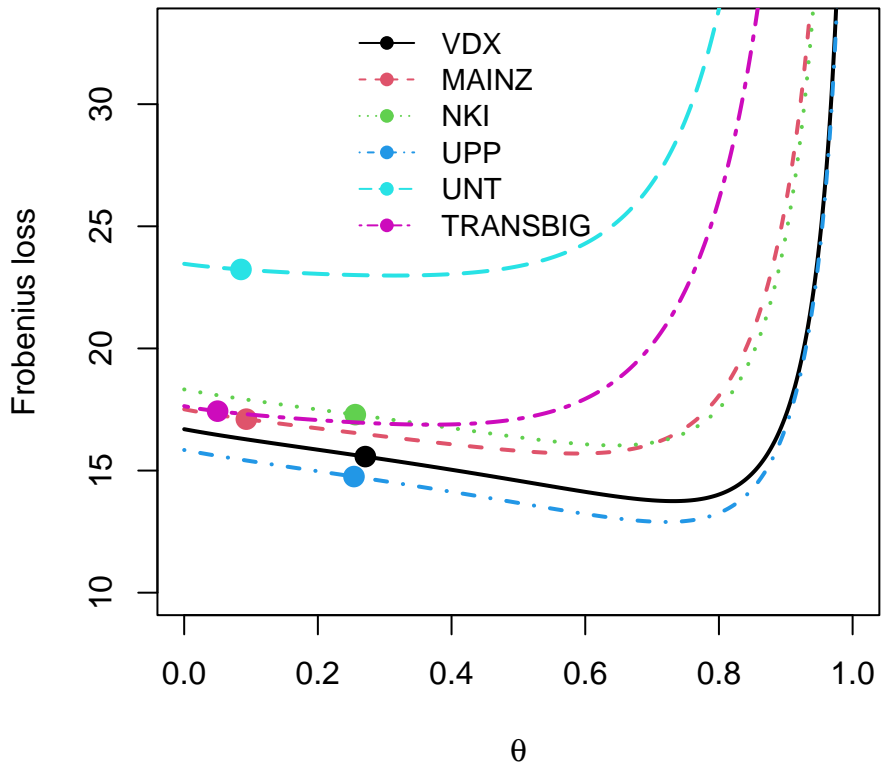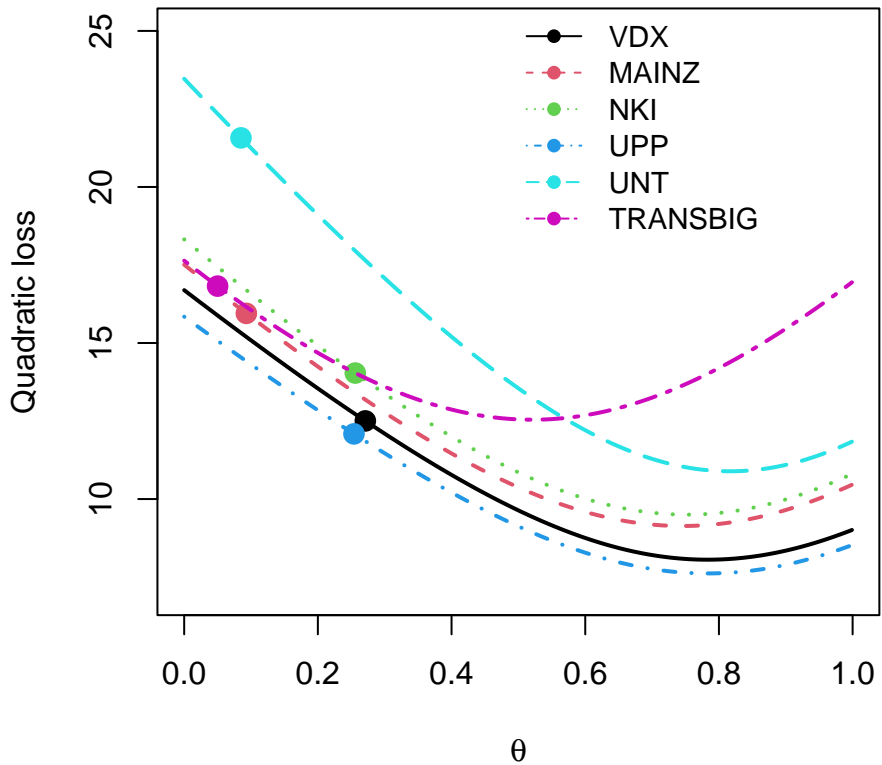

Supplement: Supplementary file 1 — Supporting File: bimj70115‐sup‐0001‐Datacode.zip. [file BIMJ-68-e70115-s001.zip › code and data/plot/TargetChoice_loss_ginv.pdf]

Loss,  $T_a$ : Gaussian LASSO

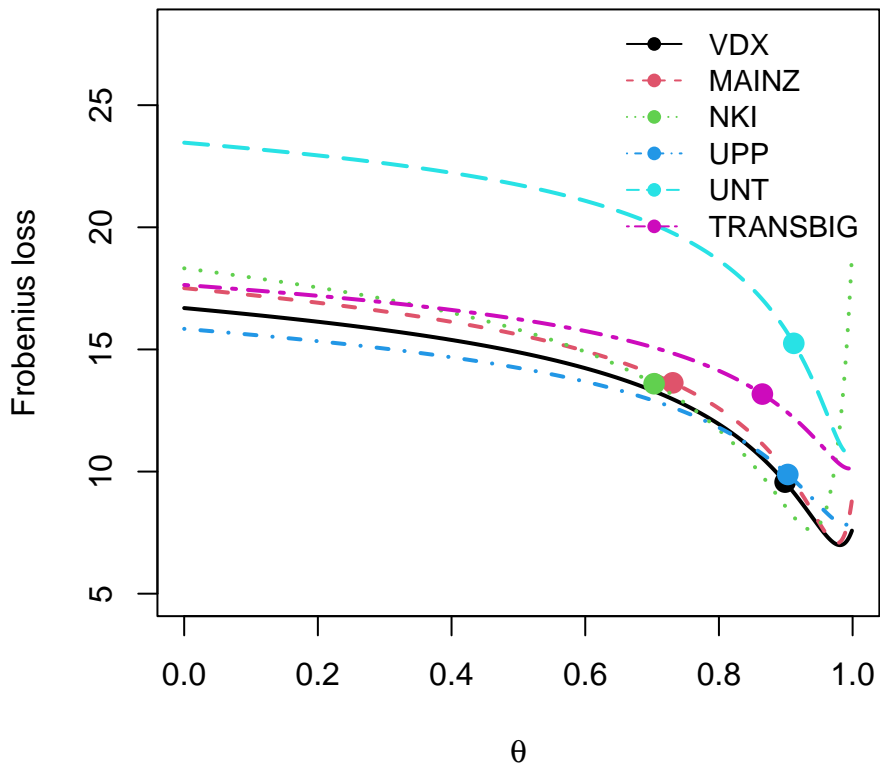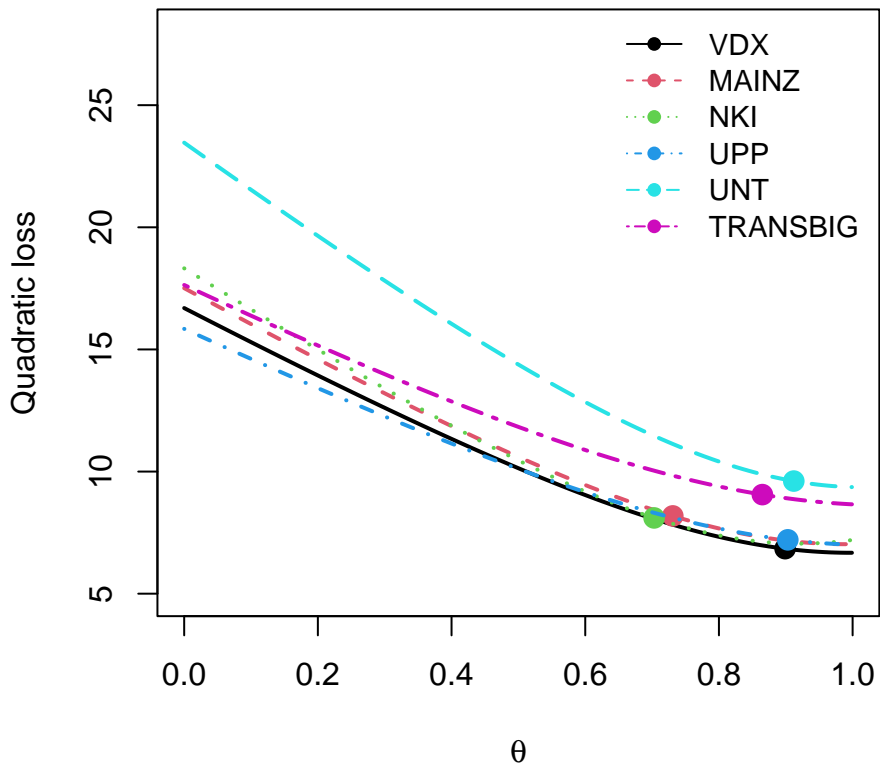

Supplement: Supplementary file 1 — Supporting File: bimj70115‐sup‐0001‐Datacode.zip. [file BIMJ-68-e70115-s001.zip › code and data/plot/TargetChoice_loss_glasso.pdf]

scale-free inverse,  $n = 50$ ,  $p = 150$ ,  $\lambda = 100$

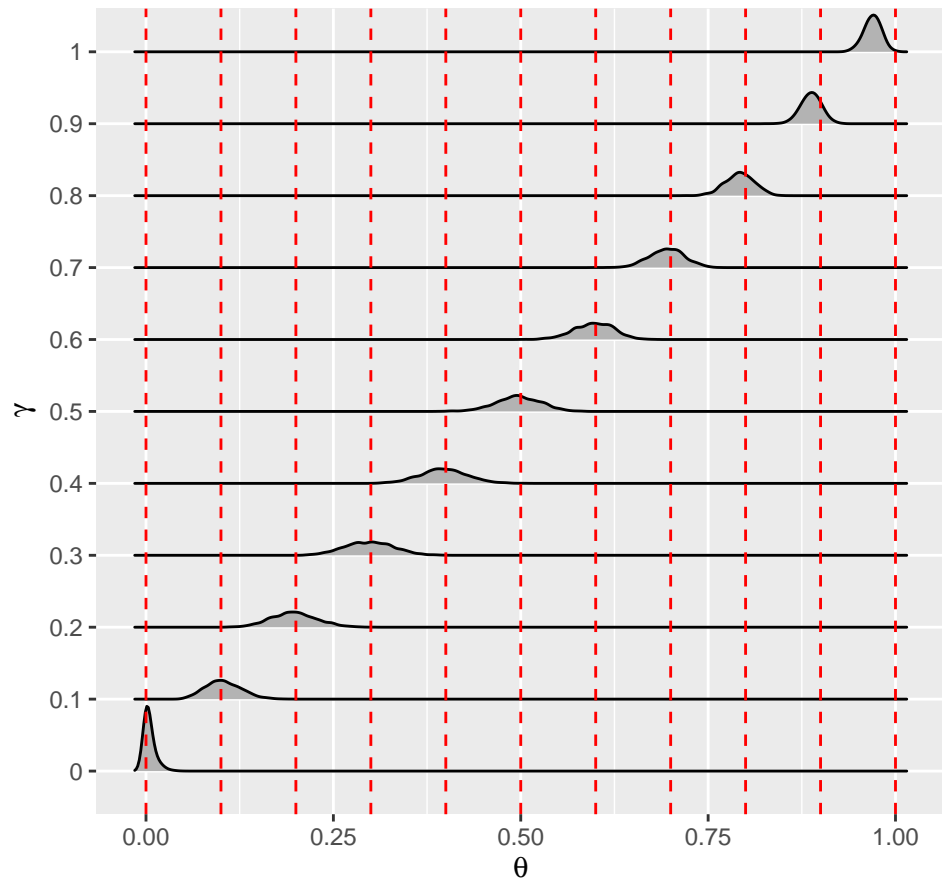

Supplement: Supplementary file 1 — Supporting File: bimj70115‐sup‐0001‐Datacode.zip. [file BIMJ-68-e70115-s001.zip › code and data/plot/unbias_finitelambda_n50_p150.pdf]

scale-free inverse,  $n = 50$ ,  $p = 300$ ,  $\lambda = 100$

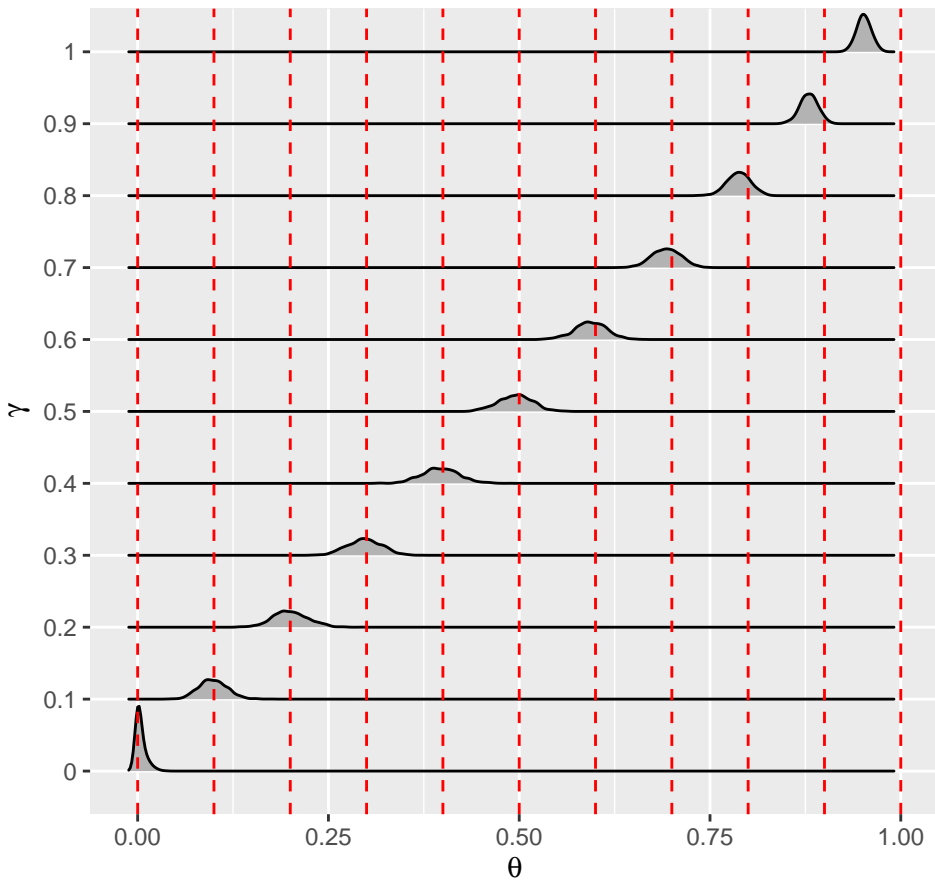

Supplement: Supplementary file 1 — Supporting File: bimj70115‐sup‐0001‐Datacode.zip. [file BIMJ-68-e70115-s001.zip › code and data/plot/unbias_finitelambda_n50_p300.pdf]

scale-free inverse,  $n = 50$ ,  $p = 50$ ,  $\lambda = 100$

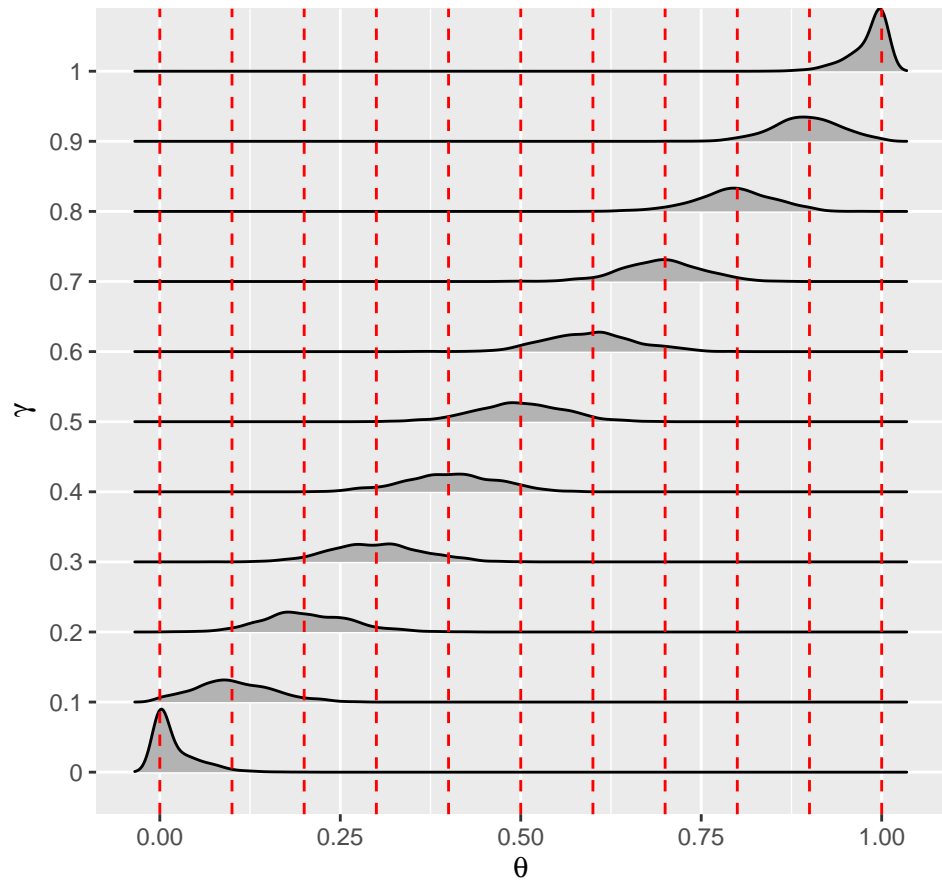

Supplement: Supplementary file 1 — Supporting File: bimj70115‐sup‐0001‐Datacode.zip. [file BIMJ-68-e70115-s001.zip › code and data/plot/unbias_finitelambda_n50_p50.pdf]

Scale-free inverse,  $n = 50$ ,  $p = 150$

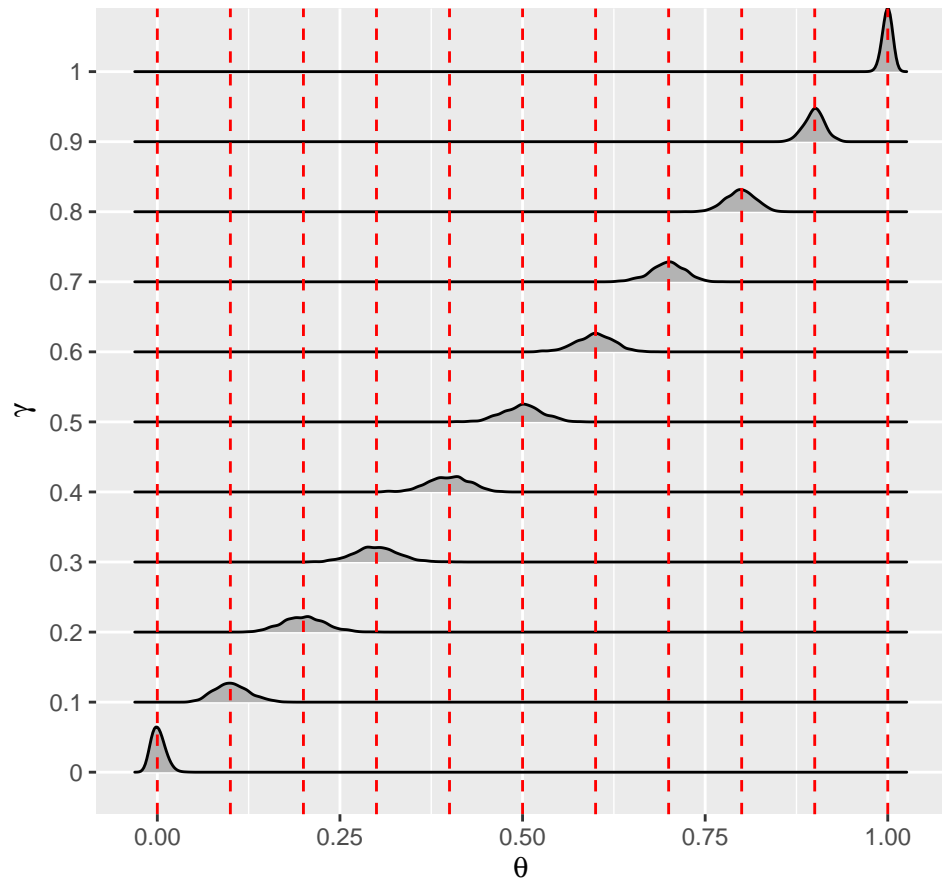

Supplement: Supplementary file 1 — Supporting File: bimj70115‐sup‐0001‐Datacode.zip. [file BIMJ-68-e70115-s001.zip › code and data/plot/unbias_infinitelambda_n50_p150.pdf]

Scale-free inverse,  $n = 50$ ,  $p = 300$

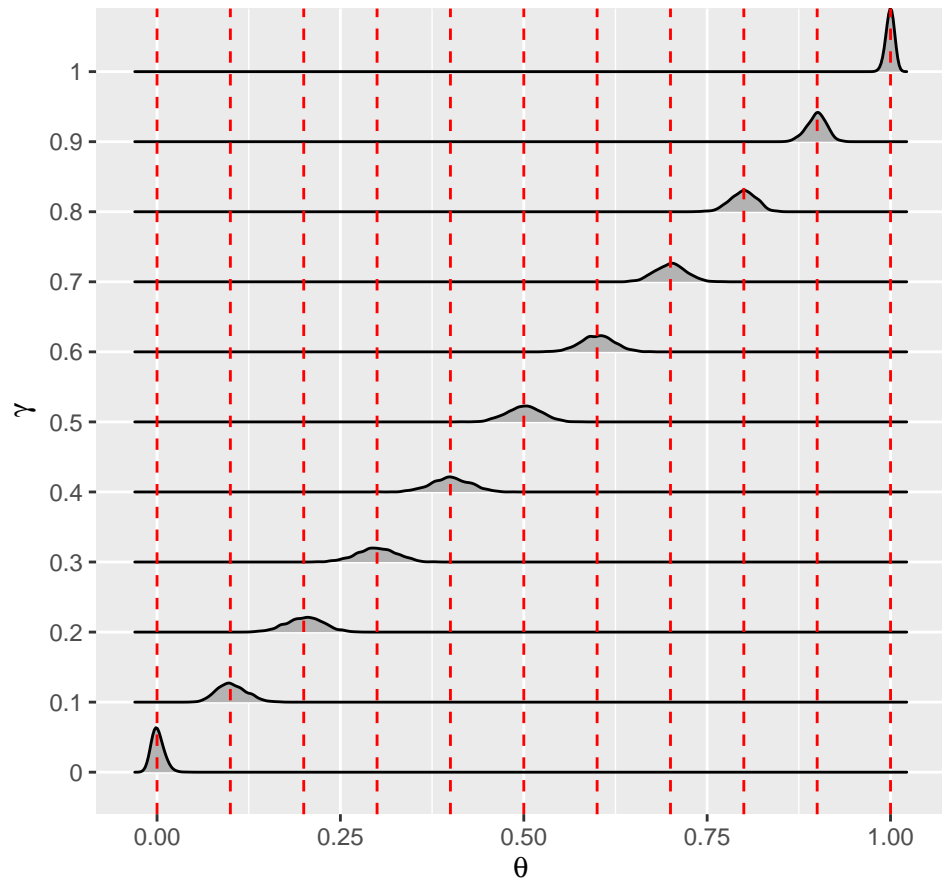

Supplement: Supplementary file 1 — Supporting File: bimj70115‐sup‐0001‐Datacode.zip. [file BIMJ-68-e70115-s001.zip › code and data/plot/unbias_infinitelambda_n50_p300.pdf]

Scale-free inverse,  $n = 50$ ,  $p = 50$

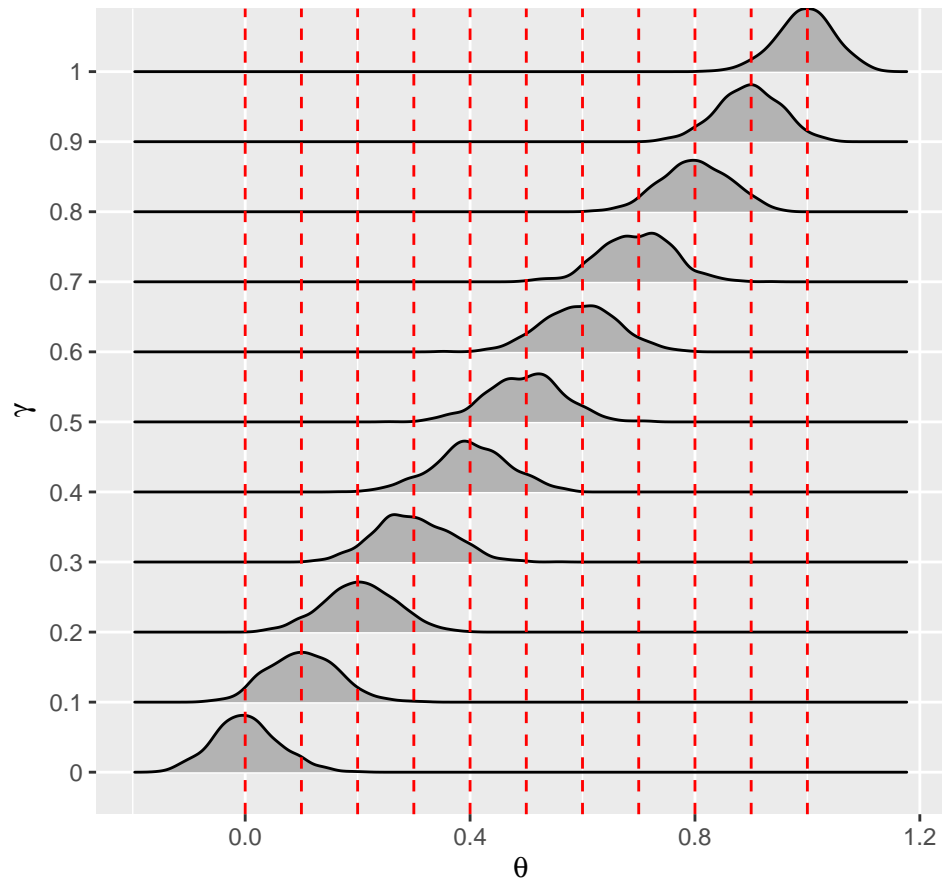

Supplement: Supplementary file 1 — Supporting File: bimj70115‐sup‐0001‐Datacode.zip. [file BIMJ-68-e70115-s001.zip › code and data/plot/unbias_infinitelambda_n50_p50.pdf]
